# Supplementary material for: Who gets referred for knee or hip replacement? A theoretical model of the potential impact of evidence-based referral thresholds using data from a retrospective review of clinic records from an English musculoskeletal referral hub
Source: BMJ Open. 2020 Jul 2;10(8):e028915. doi: 10.1136/bmjopen-2019-028915 (PMC7335627; doi:10.1136/bmjopen-2019-028915)
Supplement: Supplementary data [file bmjopen-2019-028915supp001.pdf]

Online supplementary file

Methods for stepwise regression models

We selected variables for inclusion in the logistic regression model in a pre-specified sequence. We first estimated a model only including the constant term and evaluated whether adding OKS/OHS to the model reduced Akaike information criterion (AIC); if AIC was reduced, the model used in the next step controlled for OKS/OHS and otherwise remained with just a constant term. Similarly, the next step assessed whether adding age into the model reduced AIC and in the final step we assessed whether adding sex into the model reduced AIC. We specified the order in which variables were considered for the model (OKS/OHS, then age, then sex) in advance of data analysis: it was hypothesised that OKS/OHS would be the most important factor determining referrals, followed by age (since this is a proxy for comorbidities) and finally sex (since we had no prior hypotheses about how sex would affect referrals). Age and OKS/OHS were assumed to have linear effects on the log-odds of being referred for surgical assessment as there were insufficient data to evaluate nonlinear functions.

Methods for estimating patient numbers

Hub data were extrapolated to estimate patient numbers across England by applying the probability of referral estimated on the hub data to national data on the characteristics of patients undergoing arthroplasty and the total number of operations.

We considered 490 groups of patients, defined by gender, age (using age bands <55, 55-64, 65-74, 75-84 and ≥85) and individual preoperative OKS/OHS between 0 and 48. An extract of individual patient data from the national patient reported outcome measures (PROMs) dataset linked to Hospital Episode Statistics (HES) covering operations conducted between 2009 and 2015 was used to estimate the proportion of arthroplasty patients in different groups defined by age and gender. The same dataset was used to obtain the proportion of patients with each preoperative OKS/OHS.

The number of operations in each of the 490 patients groups was calculated by multiplying the proportion of patients in different age and sex groups by the proportion with different OKS/OHS and by the number of primary knee/hip replacements conducted solely for osteoarthritis in England in 2014-15. The total number of primary knee/hip replacements conducted for osteoarthritis (76,617 knee replacements and 69,313 hip replacements) was calculated by multiplying the total number of finished consultant episodes in HES[1] with the OPCS codes beginning with O or W that the National joint Registry (NJR) used to identify primary hip/knee replacement[2] (79,726 knee replacements and 77,880 hip replacements) by the proportion of primary arthroplasty procedures that are conducted solely for osteoarthritis (96% for knees and 89% for hips).[3]

Dakin HA, *et al. BMJ Open* 2020; 10:e028915. doi: 10.1136/bmjopen-2019-028915

We then calculated the number of patients in each group who are likely to have been referred by GPs with knee/hip symptoms by dividing the number of operations in each group by the probability that each group of patients will undergo surgery.

Unit costs

The unit costs used in the current analysis are shown in Table A1. The costs and QALYs accrued in the 10-year period after arthroplasty (or a decision not to perform arthroplasty) have been reported previously.[4]

Table A1 Unit costs

Healthcare resource	Cost per consultation	Reference and details
Attendance at musculoskeletal hub	£58	Based on 40 minutes' of patient-related activity for a grade 8a physiotherapist. Based on activity at the NOC hub, each hub attendance lasts 30 minutes, plus an additional 10 minutes' administration per patient and will, on average, be conducted by a grade 8a physiotherapist who spends around 80% of their time on patient-related activity. The salary for a band 8a, point 36 physiotherapist was based on Agenda for Change salaries[5] and converted to an hourly rate by applying the same methodology as was applied by PSSRU for a hospital physiotherapist,[6] assuming that working hours and the percentages for salary on costs, qualifications and overheads would be the same for all hospital physiotherapists.
Outpatient consultation with an orthopaedic surgeon	£132	National average unit cost for first consultant-led non-admitted face-to-face attendance, WF01B, CL sheet of the National Schedule of Reference Costs Year 2013-14 NHS trusts and NHS foundation trusts[7]

Additional results

Table A2 Conditions other than osteoarthritis for which patients aged ≥50 years were excluded from the analysis

	Number of patients	
	**Knee audit**	**Hip audit**
Total patients aged ≥50 excluded due to conditions other than osteoarthritis	18	19
Rheumatoid arthritis	5	9
Bone or joint infections	4	2
Requiring limb reconstruction	1	2
Injury	2	1
No mechanical symptoms	1	1
Fracture	0	3
Arthritis secondary to fracture	2	0
Symptoms caused by previous spinal surgery	0	1
Gout	1	0
Chondroid lesion	1	0
Quadriceps rupture	1	0

Table A3 Characteristics of the patients included in the hub audit

	**Number of patients**	**Mean age (range)**	**% Female (n/N)**	**Mean OKS/OHS (range; N)**
Knee patients				
All osteoarthritis patients	315	66 (50, 93)	52% (165/315)	-
All patients referred directly to surgical assessment	71	68 (51, 86)	61% (43/71)	15 (1, 30; N=27)
Patients not referred for arthroplasty following direct referral to surgical assessment	*30*	*63 (51, 86)*	*53% (16/30)*	*21 (10, 30; N=5)*
Patients referred for arthroplasty following direct referral to surgical assessment	*38*	*72 (55, 86)*	*63% (24/38)*	*14 (1, 28; N=22)*
Patients attending neither hub nor surgical assessment	114	66 (50, 86)	49% (56/114)	-
All patients attending the hub	130	65 (50, 93)	51% (66/130)	19 (0, 48; N=130)
Patients not referred to surgical assessment following hub attendance	*61*	*64 (50, 88)*	*44% (27/61)*	*23 (1, 48; N=61)*
Patients referred to surgical assessment following hub attendance	*49*	*65 (51, 88)*	*49% (24/49)*	*18 (3, 41; N=49)*

Dakin HA, *et al. BMJ Open* 2020; 10:e028915. doi: 10.1136/bmjopen-2019-028915

	Number of patients	Mean age (range)	% Female (n/N)	Mean OKS/OHS (range; N)
Patients referred for arthroplasty following hub attendance and surgical assessment	*12*	*66 (55, 88)*	*33% (4/12)*	*17 (3, 32; N=12)*
Patients not referred for arthroplasty following hub attendance and surgical assessment	*28*	*65 (51, 82)*	*57% (16/28)*	*18 (4, 29; N=28)*
Hip patients				
All osteoarthritis patients	607	70 (50, 95)	67% (407/607)	-
All patients referred directly to surgical assessment	236	72 (50, 95)	67% (158/236)	-
Patients not referred for arthroplasty following direct referral to surgical assessment	*74*	*71 (51, 95)*	*80% (59/74)*	-
Patients referred for arthroplasty following direct referral to surgical assessment	*161*	*72 (50, 92)*	*61% (98/161)*	-
Patients attending neither hub nor surgical assessment	265	70 (50, 93)	64% (170/265)	-
All patients attending the hub	106	67 (50, 89)	75% (79/106)	23 (0, 48; N=106)
Patients not referred to surgical assessment following hub attendance	*65*	*67 (51, 89)*	*80% (52/65)*	*25 (5, 46; N=65)*
Patients referred to surgical assessment following hub attendance	*36*	*67 (50, 85)*	*67% (24/36)*	*21 (5, 44; N=36)*
Patients referred for arthroplasty following hub attendance and surgical assessment	*10*	*72 (56, 85)*	*50% (5/10)*	*19 (8, 35; N=10)*
Patients not referred for arthroplasty following hub attendance and surgical assessment	*17*	*67 (51, 84)*	*65% (11/17)*	*24 (11, 44; N=17)*

Dakin HA, *et al. BMJ Open* 2020; 10:e028915. doi: 10.1136/bmjopen-2019-028915

FIGURE A1 Distribution of (**A**) OKS and (**B**) OHS for patients attending the hub with knee/hip pain compared with the distribution of patients undergoing knee arthroplasty in England in PROMs/HES (2009-2015)

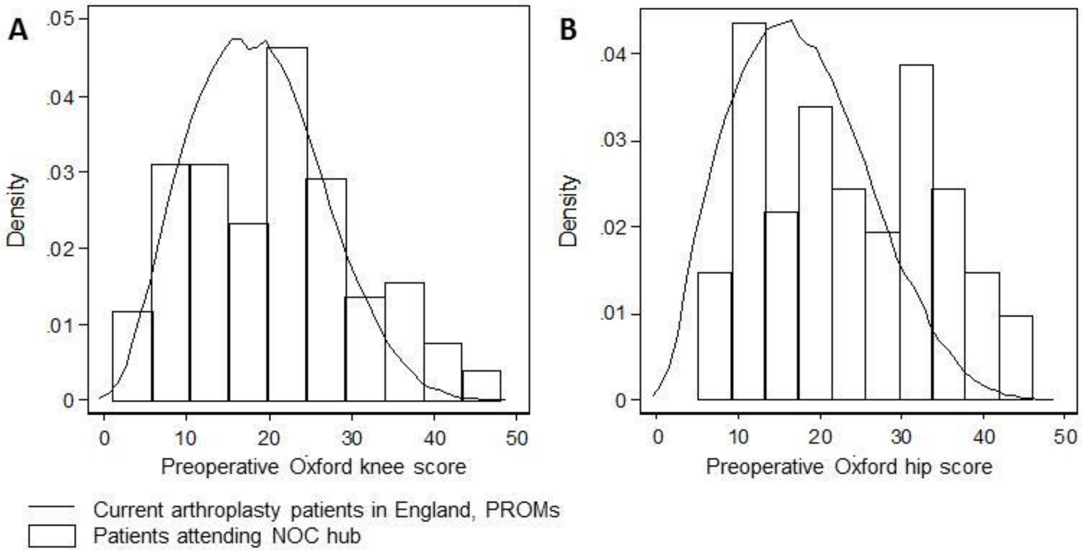

Additional regression results

For patients attending the hub with knee symptoms, adding age and sex into the model predicting the odds of hub attendees being referred for surgical assessment increased AIC, so the final model included only OKS (Table A4). Similarly, in the model of hip patients, adding age into the model worsened AIC, so the final model included only OHS and gender. Omitting gender from the model had negligible effect on the coefficient for OHS, which was 0.0397 (SE: 0.0211) when OHS was the only explanatory variable.

TABLE A4 Results of logistic regression predicting the odds of patients attending the hub being referred for surgical assessment

**Variable**	**Mean coefficient (SE)**	
	**Knee patients (N=110)**	**Hip patients (N=101)**
OKS/OHS	-0.048 (0.021)*	-0.0397 (0.0213)
Male	-	0.6965 (0.4804)
Constant	0.768 (0.459)	0.1428 (0.5376)
Pseudo-R^2	0.0389	0.0441

* p<0.05

In addition to the logistic regression model predicting the odds of patients being referred from the hub to surgical assessment, we also conducted a secondary analysis predicting the odds of undergoing arthroplasty in patients who had already been referred to surgical assessment by the hub, using the same methods. This analysis suggested that OKS/OHS, age and sex had no significant effect on the odds of referral from surgical assessment to surgery

Dakin HA, *et al. BMJ Open* 2020; 10:e028915. doi: 10.1136/bmjopen-2019-028915

(p>0.05) and that including any of these variables in the regression increased AIC compared with a model with only a constant term. This validates the assumption that the probability of patients referred directly to surgical assessment subsequently being referred for surgery is independent of OKS/OHS, age and sex and the assumption that introducing OKS/OHS thresholds at the hub would have no effect on the probability of a patient with particular preoperative characteristics being referred for arthroplasty following a surgical assessment visit. However, this analysis is likely to be underpowered, since only 12 knee patients and 10 hip patients underwent surgery after face-to-face consultations at the hub and surgical assessment.

References

1. Hospital Episode Statistics. Admitted patient care England 2014-15: Procedures and interventions. 2015. http://www.hscic.gov.uk/catalogue/PUB19124/hosp-epis-stat-admi-proc-2014-15-tab.xlsx (accessed 6 April 2016).
2. National Joint Registry for England and Wales. OPCS codes relevant procedures recorded in the NJR. 2013. http://www.njrcentre.org.uk (accessed 29 September 2016).
3. National Joint Registry for England and Wales. 13th Annual Report. 2016. www.njrcentre.org.uk (accessed 10 October 2016).
4. Dakin H, Eibich P, Beard D, Gray A, Price A, on behalf of the ACHE Study team. The use of patient-reported outcome measures to guide referral for hip and knee replacement: Part 2 – a cost-effectiveness analysis. Submitted to Bone and Joint Journal 2019
5. NHS Employers. Agenda for change pay bands and points from 1 April 2014. 2014. http://www.nhsemployers.org/~/media/Employers/Documents/Pay%20and%20reward/AfC%20pay%20bands%20and%20points%20poster%202014.pdf (accessed 19 October 2016).
6. Curtis L. Unit Costs of Health and Social Care 2014. 2014. http://www.pssru.ac.uk/project-pages/unit-costs/2014/ (accessed 22 June 2015).
7. Department of Health. NHS reference costs 2013 to 2014 - National schedule of reference costs: the main schedule. 2014. https://www.gov.uk/government/publications/nhs-reference-costs-2013-to-2014 (accessed 22 June 2015).

Dakin HA, *et al. BMJ Open* 2020; 10:e028915. doi: 10.1136/bmjopen-2019-028915
