# Supplementary material for: Who gets referred for knee or hip replacement? A theoretical model of the potential impact of evidence-based referral thresholds using data from a retrospective review of clinic records from an English musculoskeletal referral hub
Source: BMJ Open. 2020 Jul 2;10(8):e028915. doi: 10.1136/bmjopen-2019-028915 (PMC7335627; doi:10.1136/bmjopen-2019-028915)
Supplement: Author's manuscript [file bmjopen-2019-028915.draft_revisions.pdf]

BMJ Open is committed to open peer review. As part of this commitment we make the peer review history of every article we publish publicly available.

When an article is published we post the peer reviewers' comments and the authors' responses online. We also post the versions of the paper that were used during peer review. These are the versions that the peer review comments apply to.

The versions of the paper that follow are the versions that were submitted during the peer review process. They are not the versions of record or the final published versions. They should not be cited or distributed as the published version of this manuscript.

BMJ Open is an open access journal and the full, final, typeset and author-corrected version of record of the manuscript is available on our site with no access controls, subscription charges or pay-per-view fees (http://bmjopen.bmj.com).

If you have any questions on BMJ Open's open peer review process please email info.bmjopen@bmj.com

BMJ Open

Who gets referred for knee or hip replacement? A theoretical model of the potential impact of evidence-based referral thresholds using data from a retrospective review of clinic records from an English musculoskeletal referral hub

Journal:	*BMJ Open*
Manuscript ID	bmjopen-2019-028915
Article Type:	Research
Date Submitted by the Author:	02-Jan-2019
Complete List of Authors:	Dakin, Helen; University of Oxford, Health Economics Research Centre, Nuffield Department of Population Health<br>Eibich, Peter; Max-Planck-Institut fur Demografische Forschung, ; University of Oxford, Health Economics Research Centre, Nuffield Department of Population Health<br>Gray, Alastair; University of Oxford, Nuffield Department of Population Health<br>Smith, James; University of Oxford, Nuffield Department of Orthopaedics, Rheumatology and Musculoskeletal Sciences<br>Barker, Karen L.; University of Oxford, Nuffield Department of Orthopaedics, Rheumatology and Musculoskeletal Sciences<br>Beard, David; University of Oxford, Nuffield Dept of Orthopaedics, Rheumatology and Musculoskeletal Sciences<br>Price, Andrew; University of Oxford, Nuffield Department of Orthopaedics, Rheumatology and Musculoskeletal Sciences
Keywords:	Arthroplasty, hip replacement, knee replacement, prioritisation, osteoarthritis

SCHOLARONE™
Manuscripts

Who gets referred for knee or hip replacement? A theoretical model of the potential impact of evidence-based referral thresholds using data from a retrospective review of clinic records from an English musculoskeletal referral hub

Helen Dakin,[1] Peter Eibich,[1,2] Alastair Gray,[1] James Smith,[3] Karen Barker,[3] David Beard,[3] Andrew Price[3] on behalf of the ACHE Study team

[1] Health Economics Research Centre, Nuffield Department of Population Health, University of Oxford, UK
[2] Max Planck Institute for Demographic Research, Rostock, Germany
[3] Nuffield Department of Orthopaedics, Rheumatology, and Musculoskeletal Science, NIHR Biomedical Research Unit, University of Oxford, Oxford, UK

Contributors: Sujin Kang, Jonathan Cook, Ray Fitzpatrick, David Murray, Nigel Arden, Andy Carr, Stephanie Smith, Kristina Harris, Rob Middleton, Elizabeth Gibbons, Elena Benedetto, Jill Dawson, Adrian Sayers, Laura Miller, Elsa Marques, Rachael Gooberman-Hill, Ashley Blom, Andrew Judge, and Sion Glyn-Jones.

Short title for running header: Who gets referred for joint replacement?

Contact details for authors:

Helen Dakin [corresponding author and guarantor]: Senior Researcher, Health Economics Research Centre, Nuffield Department of Population Health, University of Oxford, Old Road Campus, Headington, Oxford, OX3 7LF, UK. Email: helen.dakin@ndph.ox.ac.uk; Tel: 01865 289422.

Peter Eibich: Senior Researcher, Health Economics Research Centre, Nuffield Department of Population Health, University of Oxford, Old Road Campus, Headington, Oxford, OX3 7LF, UK. Research Scientist, Max Planck Institute for Demographic Research, Konrad-Zuse-Str. 1, 18057 Rostock, Germany. Email: eibich@demogr.mpg.de.

Alastair Gray: Professor of Health Economics, Health Economics Research Centre, Nuffield Department of Population Health, University of Oxford, Old Road Campus, Headington, Oxford, OX3 7LF, United Kingdom. Email: alastair.gray@ndph.ox.ac.uk.

James Smith: Research Fellow, Nuffield Department of Orthopaedics, Rheumatology and Musculoskeletal Sciences, University of Oxford, Botnar Research Centre, Windmill Road, Headington, Oxford, OX3 7LD, UK. Email: James.smith@ndorms.ox.ac.uk.

Karen Barker: Clinical Director, Nuffield Orthopaedic Centre, Oxford University Hospitals Trust, Oxford, OX3 7HE, UK; Professor of Physiotherapy, Nuffield Department of Orthopaedics, Rheumatology and Musculoskeletal Sciences, University of Oxford, Botnar

For peer review only

Research Centre, Windmill Road, Headington, Oxford, OX3 7LD, UK. Email: Karen.Barker@ouh.nhs.uk.

David Beard: Professor of Musculoskeletal Sciences, Nuffield Department of Orthopaedics, Rheumatology and Musculoskeletal Sciences, University of Oxford, Botnar Research Centre, Windmill Road, Headington, Oxford, OX3 7LD, UK. Email: david.beard@ndorms.ox.ac.uk.

Andrew Price: Professor of Orthopaedic Surgery, Nuffield Department of Orthopaedics, Rheumatology and Musculoskeletal Sciences, University of Oxford, Botnar Research Centre, Windmill Road, Headington, Oxford, OX3 7LD, UK. Email: andrew.price@ndorms.ox.ac.uk.

Institution where the work was carried out: Health Economics Research Centre and Nuffield Department of Orthopaedics, Rheumatology, and Musculoskeletal Science, University of Oxford

Word Count: 3989 (excluding Declarations)

Tables: 2

Figures: 3

For peer review only

Abstract

Objectives: To estimate the relationship between patient characteristics and referral decisions made by musculoskeletal hubs, and assess the possible impact of an evidence-based referral tool.

Design: Audit of clinical practice and decision-tree model evaluating policy changes using audit results and national data.

Setting: One musculoskeletal interface clinic (hub) in England.

Participants: 922 adults aged ≥50 years referred by GPs with symptoms of knee or hip osteoarthritis.

Interventions: We assessed the current frequency and determinants of referrals from one hub and the change in referrals that would occur at this centre and nationally if thresholds for referral (Oxford knee and hip scores, OKS/OHS) were introduced.

Main outcome measure: OKS/OHS, referrals for surgical assessment, referrals for arthroplasty, costs and quality-adjusted life-years (QALYs).

Results: Of 110 patients with knee symptoms attending face-to-face hub consultations, 49 (45%) were referred for surgical assessment; mean OKS for these 49 patients was 18 (range: 1-41). Among hip patients, 36% (36/101) were referred for surgical assessment (mean OHS: 21, range: 5-44). No patients referred for surgical assessment were above previously-reported economic thresholds for OKS (43) or OHS (45). Setting thresholds of OKS≤31 and OHS≤35 might have resulted in an additional 22 knee referrals and 26 hip referrals in our cohort. Extrapolating hub results across England suggests a possible increase in referrals nationally, leading to around 13,000 additional knee replacements and 4,500 additional hip replacements each year.

Conclusions: Musculoskeletal hubs currently consider OKS/OHS and other factors when making decisions about referral to secondary care for joint replacement. Those referred for surgery typically have low OHS/OKS scores and introducing evidence-based OKS/OHS thresholds would prevent few inappropriate (high-functioning, low-pain) referrals. However, our findings suggest that some patients not currently referred could benefit from arthroplasty based on OKS/OHS. More research is required to explore other important patient characteristics currently influencing hub decisions.

Keywords

Arthroplasty; hip replacement; knee replacement; prioritisation; osteoarthritis.

For peer review only

Strengths and limitations of this study

- This is the first study reporting data on the characteristics of patients being referred to a UK hub with symptoms of hip or knee osteoarthritis; such data are needed to assess the impact of changing referral criteria on the number of knee/hip replacements and on costs and quality of life.
- We retrospectively reviewed clinical records for 922 men and women referred to one musculoskeletal hub.
- We evaluated how the probability of referral varies with preoperative characteristics in current clinical practice.
- We extrapolated our data nationally using a decision-tree and Markov model and estimated what impact that different policies might have based on several assumptions.
- A pilot study would be required to quantify the real-world impact of any policy change, while data from multiple hubs and information on other patient characteristics (such as BMI) are required for a comprehensive understanding of current clinical practice.

For peer review only

Introduction

Recently, many clinical commissioning groups (CCGs) in the UK have set referral criteria for joint replacement that exclude patients with Oxford knee and hip scores (OKS and OHS) above certain thresholds, which are often as low as 19[1] or 24[1-3] out of 48. Recent papers have shown that these low thresholds are inappropriate: arthroplasty has a ≥80% chance of producing a meaningful improvement in patients with OKS/OHS of 19 or 24[4] and would cost <£10,000 per quality-adjusted life-year (QALY).[5] Analysis of >400,000 medical records in the recent ACHE study suggested that thresholds between 31 and 41 may be justified on clinical and cost-effectiveness grounds.[4 5]

Commissioners' decisions concerning thresholds may be influenced by the likely number of operations and their cost. In the UK, >94% of patients currently undergoing arthroplasty have OKS/OHS ≤31, demonstrating that nearly all operations currently conducted were cost-effective and had ≥70% chance of meaningful improvement.[4 5] However, there are very little data on how patient characteristics influence the assessment process in practice, or on patients who do not currently receive surgery, making it difficult to assess the impact that different referral criteria may have on patient numbers or costs. As in many other clinical areas, hubs (also known as triage clinics, musculoskeletal interface services or intermediate musculoskeletal assessment centres) are increasingly used as gatekeepers determining access to consultations with orthopaedic surgeons, assessing all patients being considered for surgery.[6-8]

This study aimed to describe the characteristics of patients attending hub consultations, identify how patients within a hub are currently managed and estimate the relationship between preoperative characteristics and referral decisions. To illustrate how such data could be used to inform policy, we estimated the potential impact of a policy change by which patients are referred from a hub based on evidence-based OKS/OHS thresholds.

Methods

Outline

We collected data from one UK referral hub and used it to estimate a logistic regression model predicting the probability that patients will be referred from the hub to surgical assessment based on their OKS/OHS, age and sex. We then applied this regression model to nationally-collected preoperative outcomes data[10-15] to estimate how many patients are referred for surgical assessment nationally. We predicted how many of these patients would be referred through to secondary care if different OKS/OHS thresholds were introduced and potentially how many extra operations would be performed.

For peer review only - http://bmjopen.bmj.com/site/about/guidelines.xhtml

Hub data collection

Anonymised data were extracted retrospectively from medical records of patients with knee or hip symptoms who had been referred by GPs between July 2015 and July 2016 to the musculoskeletal hub at the Nuffield Orthopaedic Centre (NOC) in Oxford. This audit of practice was conducted under guidance from the Hub Lead and Clinical Director of Oxford University Hospitals (OUH R&D Ref 11603). The primary statistical analysis used a prognostic model to estimate how the probability of patients attending face-to-face consultations at the hub being referred to surgical assessments in secondary care varied with OKS/OHS, age and sex. We therefore chose the sample size to provide data on ≥30 knee referrals and ≥30 hip referrals from the hub to secondary care, thereby providing ≥10 events per explanatory variable.[9]

Two medically-qualified surgical research fellows extracted the following: age; sex; OKS/OHS at hub attendance; attendance date; whether the patient was referred to secondary care; date of any subsequent surgical assessment visit; and date of any subsequent arthroplasty surgery. Additional information on imaging, referrals to other clinics, and other surgeries or diagnoses was recorded in free text fields. Data on OKS at the surgical assessment visit were also extracted, when available, from the secondary care records of patients with knee pain. Body mass index (BMI) was not extracted as the analysis focused on OKS/OHS and available evidence on how capacity to benefit and cost-effectiveness vary with OKS/OHS does not consider BMI.[4 5] Patients who were on the waiting list for arthroplasty surgery at the time of data extraction (August 2016) and those for whom surgery was delayed due to comorbidities or high BMI after their attendance at the surgical assessment consultation were counted as having been referred for surgery.

Exclusion criteria comprised:

- Aged <50 years (for whom knee/hip pain is unlikely to be caused by osteoarthritis).
- Evidence from medical records that symptoms were due to a condition other than osteoarthritis.
- Previous arthroplasty on the same joint.
- Medical records inaccessible for research.
- Any attendance at the hub or surgical assessment unit before July 2015.

However, patients who were referred for x-rays, magnetic resonance imaging (MRI) or physiotherapy but did not attend face-to-face consultations at the hub or surgical assessment unit were included in the descriptive analysis.

Statistical modelling

Logistic regression was used to estimate a prognostic model of the local hub data predicting how OKS/OHS, age and sex affected the odds of being referred for surgical assessment following a face-to-face attendance at the hub. Age and OKS/OHS at hub attendance were

For peer review only

analysed as continuous variables. Explanatory variables were selected based on the Akaike information criterion using forward stepwise regression, manually testing variables in a pre-specified sequence (online supplementary appendix). Analyses were conducted in Stata version 14 (StataCorp, College Station TX) on a complete case basis, excluding patients with missing data. We analysed binary variables using two-sample tests of proportions and continuous variables using unpaired t-tests preceded by F-tests for equal variance. OKS/OHS in the hub sample was compared against population means[10-15] using one-sample t-tests.

Estimating the number of referrals and effect of introducing thresholds

A decision-tree model of the treatment pathway was developed based on hub data and consultations with hub staff. Using the model, hub results were extrapolated to estimate the number of patients in England who are referred for hip or knee replacement (online supplementary appendix). The overall proportion of patients referred directly to surgical assessment and the proportion attending face-to-face consultations at the hub were calculated from the hub sample. We also estimated the proportion of patients who underwent arthroplasty after being referred from the hub to surgical assessment. Logistic regression results were used to predict the probability of being referred from the hub to surgical assessment for men and women aged 50, 60, 70, 80 and 90 with different OKS/OHS. For each patient group defined by age, sex and OKS/OHS, we then estimated the number of patients referred to hubs nationally by dividing national data on the number of joint replacements currently conducted for osteoarthritis in England[10-15] by the probability that patients in each group would undergo surgery.

We then estimated how the number of referrals to surgical assessment and the number of arthroplasty procedures might change if different OKS/OHS thresholds between 18 and 45 were used to determine referral decisions during face-to-face hub consultations.

This analysis made the following assumptions:

1) Introducing OKS/OHS thresholds at the hub was assumed to have no effect on GP referrals, the hub triage or the probability of a patient with particular preoperative characteristics being referred for arthroplasty following a surgical assessment visit.
2) Since the hub audit provided no data on OKS/OHS for patients who did not attend the hub, we assumed that the probability of direct referral to surgical assessment, the probability of attending a face-to-face visit at the hub, and the probability of patients referred to surgical assessment subsequently being referred for surgery were independent of OKS/OHS, age and sex. Although symptom severity is one of the main factors considered in the hub triage, this assumption is unlikely to affect estimates of the impact of changing referral criteria applied to those patients attending the hub.
3) We assumed that the referral probabilities estimated from the NOC hub sample are broadly representative of clinical practice across the UK.

For peer review only - http://bmjopen.bmj.com/site/about/guidelines.xhtml

4) We assumed that 50% of patients who are currently not referred for surgical assessment after a face-to-face hub visit would still not be considered candidates for arthroplasty (and therefore not referred) regardless of whether thresholds were introduced. This group of patients includes patients who choose not to be referred, those who have not had a complete trial of non-operative treatment and others who are be unsuitable for surgery due to comorbidities or other factors. The remaining 50% of patients were assumed to be referred only if their OKS/OHS was below the threshold.
5) Each 40-minute hub attendance was estimated to cost £58, and surgical assessment £132 (online supplementary appendix).
6) The analyses took the perspective of the NHS, focusing on costs related to hip/knee arthroplasty. Costs of GP consultations, hub triage, x-rays, imaging, physiotherapy, injections, weight loss programs, missed appointments and referrals to other clinics were excluded as there is no reason to expect the proportion of patients requiring these services to change following the introduction of OKS/OHS thresholds.
7) The reference year for costs was 2014.

The patient numbers calculated within the decision-tree were used to estimate the cost of the referral pathway. The total 10-year costs and total QALYs estimated in a related study[5] for different patient characteristics with and without arthroplasty were applied to the number of patients expected to undergo arthroplasty or have no arthroplasty in each scenario. These figures were used to estimate the net health benefit for each scenario[16 17] (assuming a £20,000/QALY ceiling ratio) and the incremental cost-effectiveness of different pairs of scenarios (online supplementary appendix).

We also estimated the number of additional referrals that might have been observed within the population of individuals attending the NOC hub if a fixed OKS/OHS threshold were introduced. Based on assumption (4), this was equal to half the number of patients who had OKS/OHS at or below the proposed threshold but were not referred, minus the number who were referred with OKS/OHS above the proposed threshold.

Public and Patient Involvement

Patient representatives were involved in the grant application and design of the wider ACHE study.[4] Results of this part of the study were discussed with a user group including clinicians and patient representatives; this discussion helped inform the interpretation and presentation of results.

For peer review only - http://bmjopen.bmj.com/site/about/guidelines.xhtml

Results

Current referral pathway

From the 1,638 records reviewed in the audit, we identified 315 patients with knee osteoarthritis (Figure 1) and 607 with hip osteoarthritis (Figure 2): 922 in total.

At the time of this audit, the musculoskeletal hub service was the only route for patients in Oxfordshire to access NHS elective orthopaedic surgery. When GPs refer patients to secondary care with knee/hip symptoms, the referral letter and x-rays are first reviewed by senior hub staff, who decide whether to (a) refer the patient directly to surgical assessment, (b) invite them to attend the hub clinic for an individual triage appointment or (c) manage them in primary care (Figure 3). Triage is based on patient symptoms as described in the GP's referral letter (which will include BMI and may include OKS/OHS) and conservative treatments already given (such as anti-inflammatory drugs, physiotherapy and weight loss strategies).

Of the osteoarthritis patients included in the analysis, 23% (71/315) of knee patients and 39% (236/607) of hip patients were referred directly to surgical assessment based on the hub triage. Anecdotally, those patients referred directly to surgical assessment tend to be those with severe symptoms, those who had already exhausted all conservative measures and those who had previously been referred for surgical assessments, but had chosen not to have surgery at that time. Among 27 patients referred directly to surgical assessment for whom OKS data were available, the mean OKS was 15 (range: 1-30); no such data were available for hip patients.

Those patients who are referred for surgical assessment discuss the risks and benefits of surgery with an orthopaedic surgeon and make an informed decision about whether or not to undergo arthroplasty or other surgery, taking into account their comorbidities, symptom severity and other factors. Among the patients who were referred directly for surgical assessment and had outcomes data available, 56% (38/68) of knee patients and 69% (161/235) hip patients underwent or were awaiting knee/hip replacements at the time of data extraction.

Following triage, 36% (114/315) of knee patients and 44% (265/607) hip patients did not attend face-to-face consultations at either the hub or the surgical assessment. Typically such patients comprise those who can be managed in primary care: for example those with mild symptoms and those who have not yet exhausted conservative treatment options, such as advice and information, activity and exercise and weight loss.[18-20] Across the 379 hip and knee patients without face-to-face consultations, 270 (71%) had x-rays, MRI or ultrasound to identify the most appropriate care pathway. Eleven (3%) were referred for physiotherapy, while seven patients (2%), all with hip osteoarthritis, had injections.

For peer review only - http://bmjopen.bmj.com/site/about/guidelines.xhtml

Anecdotally, other patients may not be seen face-to-face as they are unfit for surgery or have recent injuries likely to heal without further intervention. Eighteen patients opted for non-NHS care, missed/failed to book hub appointments, died before the hub attendance or were still awaiting a hub appointment at the time of data extraction (Figures 1 and 2).

The remaining 44% (130/315) of knee patients and 17% (106/607) of hip patients attended face-to-face assessments at the musculoskeletal hub, where extended-scope specialist physiotherapists or orthopaedic fellows assess patients to confirm diagnosis. Patients routinely complete OKS/OHS questionnaires to assess whether the level of symptoms warrants surgery and to guide discussions about the symptom profile. Among the patients attending the hub who had complete data, the mean OKS was 21 (range: 1-48), while mean OHS was 24 (range: 5-46); both values were significantly higher than the average for patients undergoing arthroplasty nationally (mean: 18; $p \leq 0.015$), although only OHS was significantly different from the average for patients undergoing arthroplasty in Oxfordshire (mean OKS: 20, $p=0.134$; mean OHS: 19 $p<0.0001$).[10-15]

Diagnostic imaging, landmark injections and injections for trochanteric bursitis may be done during the hub consultation. Patients with BMI ≥40 are referred for monitored weight loss programs that must be followed for 12 months before considering surgery. Staff and patients also discuss the risks and benefits of joint replacement (including recovery times, the need for support at home after hospital discharge and the potential need for revision surgery) and how these may be affected by patients' living arrangements and comorbidities. Following the hub visit, 45% (49/130) of knee patients and 36% (36/101) of hip patients were referred for surgical assessment. This includes patients who are considered candidates for arthroplasty or other procedures (e.g. arthroscopy, anterior cruciate ligament repair or interventional radiology).

Patients with higher OKS/OHS were significantly less likely to be referred for surgical assessment: each one-point increase in OKS reduced the odds of referral by 4.7% ($p=0.019$), while a one-point increase in OHS reduced the odds by 3.9% ($p=0.062$). Those patients who are not referred to surgical assessment may be referred for physiotherapy, other non-surgical management or other outpatient clinics (e.g. rheumatology or sports injury clinics), or may choose not to be referred as they prefer not to undergo surgery at the current time.

Across the hub attendees who were referred for surgical assessment and had data on clinical outcomes, 30% (12/40) of knee patients and 37% (10/27) of hip patients underwent or were awaiting arthroplasty surgery.

Applying estimates of the probability of referral and subsequent arthroplasty from the hub audit to national data on the distribution of patients undergoing knee/hip arthroplasty suggests that GPs in England refer around 417,000 patients with knee osteoarthritis and

For peer review only - http://bmjopen.bmj.com/site/about/guidelines.xhtml

around 235,000 patients with hip osteoarthritis to secondary care each year (Figure 3). Of these, around 172,000 knee patients and 41,000 hip patients might attend a hub if the care pathway in Oxfordshire were followed nationally; such hub attendances would cost a total of £12 million.

Effect of introducing referral thresholds

We used the hub data to estimate the impact that different policies for using OKS/OHS to guide referral decisions might have on patient numbers, costs and health outcomes. The ACHE study reported that people with OKS ≤31 have a ≥70% chance of achieving a seven-point increase in OKS following knee arthroplasty.[4] Of the 110 knee patients attending the NOC hub, 94 (85%) had OKS ≤31, of whom 47 (50%) were referred for surgical assessment. Two patients were referred with OKS of 32 or 41. Assuming that 50% of the NOC patients with OKS ≤31 who are not currently referred might still choose not to be referred or might be considered clinically inappropriate for other reasons, a fixed referral threshold OKS of 31 would have resulted in an additional 22 referrals to surgical assessment: a 49% increase.

Applying the results of logistic regression to the whole population undergoing surgery nationally suggested that an OKS threshold of 31 would result in 43,000 additional surgical assessment visits and 13,000 additional knee replacement procedures in England each year (Table 1). Based on the costs and outcomes for patients with and without knee arthroplasty that were estimated in a recent economic evaluation,[5] introducing this policy would cost the NHS an additional £36 million for each annual cohort of patients (of which £5.8 million would be due to additional surgical assessment visits), but would gain 30,000 QALYs. This policy would be highly cost-effective compared with current practice, costing just £1,195 per QALY gained, and the net health benefit would be 28,000 QALYs even after subtracting the health benefits that would be foregone by spending £36 million on knee arthroplasty candidates, rather than other conditions. Introducing higher OKS thresholds would produce still greater health benefits and would continue to be cost-effective at a £20,000/QALY ceiling ratio right up to the economic threshold of 43.[5]

Similarly, ACHE predicts that people with preoperative OHS ≤35 have a ≥70% chance of achieving an eight-point increase in OHS following hip arthroplasty.[4] Within the NOC hub, 87% (88/101) of patients had OHS ≤35, of whom 35 were referred. One patient was referred with OHS of 44. Introducing a threshold of 35 might therefore result in 26 additional referrals: a 30% increase. Extrapolating across England, we might expect 14,000 additional surgical assessments and 5,000 additional hip replacements each year. The impact of this policy is lower than for knee osteoarthritis as 90% of hip replacements are done in patients who were referred directly, and did not attend the hub. The policy would cost an additional £25 million (of which additional surgical assessments account for £2 million) and gain 16,000 QALYs, costing £1,560/QALY gained and generating a net health benefit of 15,000 QALYs.

For peer review only - http://bmjopen.bmj.com/site/about/guidelines.xhtml

Introducing higher thresholds (up to the economic threshold of 45) would produce greater health benefits and continue to cost <£20,000/QALY gained.

Discussion

Although referral hubs are being used increasingly as gatekeepers determining access to consultations in different clinical areas of the NHS, there has been little research on them. This study has characterised the patients being referred to and from a musculoskeletal hub, and showed that OKS/OHS and other factors were considered when deciding which patients to refer for surgical assessment. Since most patients attending hubs have relatively severe osteoarthritis symptoms and those with high OKS/OHS are less likely to be referred for surgery, introducing evidence-based OKS/OHS thresholds for arthroplasty is unlikely to prevent large numbers of inappropriate referrals. However, since recent evidence demonstrates that arthroplasty is both cost-effective and highly likely to benefit people with OKS/OHS well above the thresholds currently used by some CCGs,[4 5] such policies are also likely to identify patients who are not currently referred, but for whom arthroplasty could be expected to be beneficial and cost-effective. These numbers are uncertain, but extrapolations from the hub examined here suggest that, across England, setting thresholds of OKS ≤31 and OHS ≤35 would result in many more patients being referred for surgical assessment, and could result in an additional 13,000 knee replacements and 4,500 hip replacements each year. More evidence is therefore urgently needed on the other factors currently influencing both the decision to refer from the hub to surgical assessment and the decision on whether arthroplasty is the appropriate treatment option.

To our knowledge our study is the first to report the characteristics of patients attending a musculoskeletal hub. However, the analysis was based on a small sample and included only eight hub attendees with OKS/OHS ≥40. Data were analysed one month after the end of the period studied; some patients may therefore have gone on to have arthroplasty after physiotherapy or weight loss treatment. Furthermore, the retrospective review of medical records may not have identified all patients meeting exclusion criteria – particularly for patients who did not attend either the hub or surgical assessment. Furthermore, no data were collected on BMI.

Estimates of the impact of different policies rely on additional assumptions and represent an approximate indication of potential patient numbers; pilot studies would be required to assess the true impact in practice. In particular, we focused on the impact of changing referral guidelines at hubs, as no data were available on patients visiting GPs with osteoarthritis symptoms. In practice, any decision aid or referral guideline is also likely to affect GPs' referral decisions, the hub triage process and (potentially) consultations with orthopaedic surgeons. The impact of such policies could be much greater in these settings – particularly for hip replacement, where only 10% of patients undergoing arthroplasty attend

For peer review only - http://bmjopen.bmj.com/site/about/guidelines.xhtml

the hub. We also assumed, arbitrarily, that 50% of people with OKS/OHS below the threshold who are not currently referred would choose not to be referred for surgery even if this were offered.

Although access to joint replacement may be restricted if funding for interventions is reduced, results suggest that increased numbers of knee/hip replacements could be justified on cost-effectiveness grounds. Our results suggest that the NHS is currently willing to pay no more £2,000 per QALY gained from arthroplasty, while NICE routinely approves treatments for patients with other conditions (e.g. rheumatoid arthritis) that cost £20,000[23] or even £40,000[24]/QALY. This suggests that it may be more efficient and more equitable to spend limited NHS funds on conducting more joint replacements, increasing the operation rate in the UK to a level similar to that of Austria or Germany,[25] rather than directing these resources to other conditions. However, the number of procedures is also limited by availability of surgeons, operating theatres and hospital beds, which may mean that it is not currently feasible to conduct all of the operations that could be justified on cost-effectiveness grounds. And, as noted earlier, OKS/OHS is clearly not the only factor taken into account in decision-making by hubs and surgeons. In particular, patient choice plays a substantial role and comorbidities and other factors are also taken into account.

Referral hubs are an effective mechanism for ensuring that patients receive cost-effective treatment in the most appropriate healthcare setting and patients are generally satisfied with this model of care.[8] One aim of introducing hubs was to improve efficiency in the referral pathway by increasing the proportion of referrals to secondary care that lead to surgery. The hub identifies patients who do not want surgery or who have mild symptoms and directs patients to other effective interventions when surgery is not appropriate. If the service works well, only patients who are suitable for surgery are referred. However, the process and criteria for arthroplasty referrals still vary greatly between CCGs.[1] Explicit guidelines and decision aids could help reduce geographical inequity and ensure that all patients likely to benefit have fair access to cost-effective treatments. Although our study is based on retrospective data from only one hub, it provides initial estimates of what potential patient numbers and costs might be if a more explicit threshold-based approach was adopted nationally. While arthroplasty rates and patient characteristics in Oxfordshire are similar to the national average,[26 27] referral guidelines and pathways vary substantially between CCGs, suggesting that a broader audit covering multiple hubs is necessary to provide a more comprehensive picture of current clinical practice. Ideally this would also collect more detailed patient information (e.g. BMI).

In conclusion, our paper has estimated the effect of introducing evidence-based OKS/OHS thresholds in England. While our results can be used to estimate the impact of other policies, including those where thresholds vary by age or other patient characteristics, a

For peer review only

pilot study would be required to evaluate the actual impact of a policy change on routine clinical practice.

For peer review only

Declarations

Competing interest statement

All authors have completed the ICMJE uniform disclosure form at www.icmje.org/coi_disclosure.pdf and declare: a grant from NIHR for the submitted work; HD reports personal fees from Halyard Health, outside the submitted work; DB reports grants from Zimmer Biomet, outside the submitted work; AP reports personal fees from Zimmer Biomet and DePuy outside the submitted work; all declare that there are no other relationships or activities that could appear to have influenced the submitted work. The other authors declare that there are no relevant conflicts of interests besides the NIHR grant that supports the current work.

Details of contributors

HD is the guarantor for this study. AP, DB, HD, AG and KB conceived, designed and conducted the ACHE study. HD, AP and JS conceived and designed the audit, which was conducted by HD and JS with assistance from Reza Mafi, and Sarah Dorman. HD, AG and PE conceived and designed the economic evaluation. HD conducted the statistical and economic analysis under the supervision of AG. AP and KB helped interpret the results. HD drafted the manuscript. All authors edited the manuscript for important intellectual content and approved the final version.

The following members of the ACHE study group all contributed to the developing the research question, planning the protocol of work, gaining funding and/or delivery of this research project: Ray Fitzpatrick, David Murray, Nigel Arden, Andy Carr, Karen Barker, James Smith, Stephanie Smith, Kristina Harris, Rob Middleton, Elizabeth Gibbons, Elena Benedetto, Jill Dawson, Adrian Sayers, Laura Miller, Elsa Marques, Rachael Gooberman-Hill, Ashley Blom, Andrew Judge, and Sion Glyn-Jones.

Copyright/licence

The Corresponding Author has the right to grant on behalf of all authors and does grant on behalf of all authors, an exclusive licence (or non-exclusive for government employees) on a worldwide basis to the BMJ Publishing Group Ltd and its Licensees to permit this article (if accepted) to be published in BMJ Open and any other BMJPGL products to exploit all subsidiary rights, as set out in our licence http://authors.bmj.com/submitting-your-paper/copyright-and-authors-rights/ and the Corresponding Author accepts and understands that any supply made under these terms is made by BMJPGL to the Corresponding Author. Where the Corresponding Author wishes to make the article available on an open access basis (and intends to pay the relevant open access fee), the terms of such open access shall be governed by a Creative Commons licence – details of these licences and which Creative Commons licence will apply to this article are set out in our licence referred to above.

For peer review only

Ethics statement

The audit of practice was conducted under guidance of the Hub Lead and Clinical Director of OUH (OUH R&D Ref 11603) and did not require ethical approval. The audit was conducted retrospectively on patients who had received normal clinical management. All data were anonymised by clinical staff.

Funding and role of study sponsors

The ACHE study and economic evaluation was funded by the NIHR Health Technology Assessment Programme (project number 11/63/01) and will be published in full in *Health Technology Assessment*. Further information available at: https://www.journalslibrary.nihr.ac.uk/programmes/hta/116301. This report presents independent research commissioned by the National Institute for Health Research (NIHR). The views and opinions expressed by authors in this publication are those of the authors and do not necessarily reflect those of the NHS, the NIHR, MRC, CCF, NETSCC, the Health Technology Assessment Programme, NHS, NHS Digital or the Department of Health. The funder had no involvement in: the collection, analysis, and interpretation of data; in the writing of the report; and in the decision to submit the article for publication. The authors had full access to the data (including statistical reports and tables), can take responsibility for the integrity of the data and the accuracy of the data analysis and are responsible for submitting this paper.

Transparency declaration

The manuscript's guarantor (HD) affirms that the manuscript is an honest, accurate, and transparent account of the study being reported; that no important aspects of the study have been omitted; and that any discrepancies from the study as planned (and, if relevant, registered) have been explained.

Data sharing

The decision tree and Markov models used in this paper are available from the corresponding author on request. PROMs/HES data are available from NHS Digital (http://content.digital.nhs.uk/dars).

Acknowledgements

We would like to thank the ACHE patient and public representatives, user group and steering group for their role in the project: Anne Clarkson Webb, Anthony Johnson, Chad Lion Cachet, Fiona Watt, Fraser Old, Gill Dean, Gillian Kempster, Jannie Kramer, Jennifer Bostock, Jiyang Li, Jo Hewanicka, John Nolan, Kate Jackson, Laura Ingle, Mary Snow, Matthew Cheetham, Patricia Mary Rubery, Sharon Barrington, Sujin Kang, Tim Wilton, Vida Field, Sunny Deo, Malvin Drakely, Jon Campion, George Peat, Peter Kay, Jon Waite and Sue Woollacott. We would like to extend thanks to Reza Mafi, and Sarah Dorman who helped

For peer review only

with data cleaning. Data on NHS PROMs linked to HES APC data were reused with the permission of NHS Digital, copyright 2015, with all rights reserved.

Patient involvement

The research question and study design (prior to funding) was informed by patient interest groups. The study was based on pre-existing data from medical records so no patients were directly recruited in the course of the work. Throughout the study, a User Group, including patients and other stakeholders, informed progress and development of the work. Results of the audit were discussed with user group including clinicians and patient representatives, to inform the presentation of results. The results will not be directly disseminated to patients whose records were included in the audit, but the publication will be available for the target population.

References

1. The Royal College of Surgeons of England. Is access to surgery a postcode lottery? Secondary Is access to surgery a postcode lottery? 2014. https://www.rcseng.ac.uk/news-and-events/media-centre/press-releases/many-ccgs-are-ignoring-clinical-evidence-in-their-surgical-commissioning-policies/.
2. Harrogate and Rural District Clinical Commissioning Group. Clinical thresholds: Hip and knee arthroplasty for osteoarthritis (only). Secondary Clinical thresholds: Hip and knee arthroplasty for osteoarthritis (only). 2014. http://www.harrogateandruraldistrictccg.nhs.uk/data/uploads/rss2/hip-and-knee-arthroplasty.pdf.
3. Scarborough and Ryedale Clinical Commissioning Group. Hip replacement pathway. Secondary Hip replacement pathway 2015. http://www.scarboroughryedaleccg.nhs.uk/data/uploads/rss2/orthopaedics/hip-replacement-march-2015.pdf.
4. Price A, Kang S, Cook J, et al. The use of patient-reported outcome measures to guide referral for hip and knee replacement: Part 1 – the development of evidence based thresholds. Submitted to BMJ 2018
5. Dakin H, Eibich P, Beard D, Gray A, Price A, on behalf of the ACHE Study team. The use of patient-reported outcome measures to guide referral for hip and knee replacement: Part 2 – a cost-effectiveness analysis. Submitted to BMJ 2018
6. Podmore B, Hutchings A, Durand MA, et al. Comorbidities and the referral pathway to access joint replacement surgery: an exploratory qualitative study. BMC health services research 2018;**18**(1):754 doi: 10.1186/s12913-018-3565-0[published Online First: Epub Date]|.
7. Button K, Morgan F, Hodgson H, Weightman AL, Jones S. Musculoskeletal care pathways for adults with hip and knee pain at the interface between primary and secondary

For peer review only - http://bmjopen.bmj.com/site/about/guidelines.xhtml

care: protocol for a systematic review. Syst Rev 2016;**5**(1):128 doi: 10.1186/s13643-016-0301-x[published Online First: Epub Date]|.

8. Candy E, Haworth-Booth S, Knight-Davis M. Review of the Effectiveness of a Consultant Physiotherapy-Led Musculoskeletal Interface Team: A Welsh Experience. Musculoskeletal Care 2016;**14**(3):185-91 doi: 10.1002/msc.1122[published Online First: Epub Date]|.

9. Moons KG, Royston P, Vergouwe Y, Grobbee DE, Altman DG. Prognosis and prognostic research: what, why, and how? BMJ 2009;**338**:b375 doi: 10.1136/bmj.b375[published Online First: Epub Date]|.

10. Health and Social Care Information Centre. Finalised Patient Reported Outcome Measures (PROMs) in England, April 2014 to March 2015 (annual report). Secondary Finalised Patient Reported Outcome Measures (PROMs) in England, April 2014 to March 2015 (annual report) 2016. http://content.digital.nhs.uk/catalogue/PUB21189/final-proms-eng-apr14-mar15-fin-report.pdf.

11. Health and Social Care Information Centre. Finalised Patient Reported Outcome Measures (PROMs) in England, April 2013 to March 2014. Secondary Finalised Patient Reported Outcome Measures (PROMs) in England, April 2013 to March 2014 2015. http://www.hscic.gov.uk/catalogue/PUB17876/final-proms-eng-apr13-mar14-fin-report-v1.pdf.

12. Health and Social Care Information Centre. Finalised Patient Reported Outcome Measures (PROMs) in England, April 2012 to March 2013 (annual report). Secondary Finalised Patient Reported Outcome Measures (PROMs) in England, April 2012 to March 2013 (annual report) 2014. http://content.digital.nhs.uk/catalogue/PUB14574/final-proms-eng-apr12-mar13-fin-report-v1.pdf.

13. Health and Social Care Information Centre. Finalised Patient Reported Outcome Measures (PROMs) in England, April 2011 to March 2012 (annual report). Secondary Finalised Patient Reported Outcome Measures (PROMs) in England, April 2011 to March 2012 (annual report) 2013. http://content.digital.nhs.uk/catalogue/PUB11359/final-proms-eng-apr11-mar12-fin-report-v2.pdf.

14. Health and Social Care Information Centre. Finalised Patient Reported Outcome Measures (PROMs) in England, April 2010 to March 2011 (annual report). Secondary Finalised Patient Reported Outcome Measures (PROMs) in England, April 2010 to March 2011 (annual report) 2012. http://content.digital.nhs.uk/catalogue/PUB07049/fina-prom-eng-apr-10-mar-11-pre-post-rep1.pdf.

15. Health and Social Care Information Centre. Finalised Patient Reported Outcome Measures (PROMs) in England, April 2009 to March 2010 (annual report). Secondary Finalised Patient Reported Outcome Measures (PROMs) in England, April 2009 to

For peer review only

March 2010 (annual report) 2011. http://content.digital.nhs.uk/catalogue/PUB02429/proms-eng-apr-09-mar-10-oper-data-expe-meth.pdf.

16. Coyle D, Buxton MJ, O'Brien BJ. Stratified cost-effectiveness analysis: a framework for establishing efficient limited use criteria. Health Econ 2003;**12**(5):421-7 doi: 10.1002/hec.788[published Online First: Epub Date]|.

17. Espinoza MA, Manca A, Claxton K, Sculpher MJ. The value of heterogeneity for cost-effectiveness subgroup analysis: conceptual framework and application. Med Decis Making 2014;**34**(8):951-64 doi: 10.1177/0272989X14538705[published Online First: Epub Date]|.

18. National Institute for Health and Care Excellence. Osteoarthritis: NICE quality standard 87. Secondary Osteoarthritis: NICE quality standard 87 June 2015. https://www.nice.org.uk/guidance/qs87.

19. National Institute for Health and Care Excellence. Osteoarthritis: the care and management of osteoarthritis in adults: NICE clinical guideline 177. Secondary Osteoarthritis: the care and management of osteoarthritis in adults: NICE clinical guideline 177 February 2014. https://www.nice.org.uk/guidance/cg177.

20. Royal College of Surgeons. Commissioning guide: Painful osteoarthritis of the knee, Version 1.1. Secondary Commissioning guide: Painful osteoarthritis of the knee, Version 1.1 July 2014. https://www.boa.ac.uk/wp-content/uploads/2015/01/Enc-8d_Painful-osteoarthritis-of-the-knee_Revised-final.pdf.

21. Thames Valley Priorities Committee. Thames Valley Priorities Committee Commissioning Policy Statement. Policy No. 187b (TVPC30) Primary hip joint replacement for patients with osteoarthritis of the hip. Secondary Thames Valley Priorities Committee Commissioning Policy Statement. Policy No. 187b (TVPC30) Primary hip joint replacement for patients with osteoarthritis of the hip November 2015. http://www.oxfordshireccg.nhs.uk.

22. Thames Valley Priorities Committee. Thames Valley Priorities Committee Commissioning Policy Statement. Policy No. 188a (TVPC7) Primary total knee replacement surgery for patients with osteoarthritis of the knee. Secondary Thames Valley Priorities Committee Commissioning Policy Statement. Policy No. 188a (TVPC7) Primary total knee replacement surgery for patients with osteoarthritis of the knee October 2016. http://www.oxfordshireccg.nhs.uk/professional-resources/documents/commissioning-statements/188a-Total-Knee-Joint-Replacement.pdf.

23. National Institute for Health and Clinical Excellence. Social value judgements: Principles for the development of NICE guidance. Second Edition. Secondary Social value judgements: Principles for the development of NICE guidance. Second Edition 2008. http://www.nice.org.uk/media/C18/30/SVJ2PUBLICATION2008.pdf.

For peer review only - http://bmjopen.bmj.com/site/about/guidelines.xhtml

24. Dakin H, Devlin N, Feng Y, Rice N, O'Neill P, Parkin D. The Influence of Cost-Effectiveness and Other Factors on Nice Decisions. Health Econ 2015;**24**(10):1256-71 doi: 10.1002/hec.3086[published Online First: Epub Date]|.

25. OECD. Hip and knee replacement. Health at a Glance 2015: OECD, 2015:112–13.

26. Right Care. The NHS Atlas of variation in health care: reducing unwarranted variation to increase value and improve quality. Secondary The NHS Atlas of variation in health care: reducing unwarranted variation to increase value and improve quality 2010. http://fingertips.phe.org.uk/documents/Atlas_2011_MSK.pdf.

27. Health and Social Care Information Centre. Finalised Patient Reported Outcome Measures in England - April 2014 to March 2015: Score Comparison Tool. Secondary Finalised Patient Reported Outcome Measures in England - April 2014 to March 2015: Score Comparison Tool 2016. http://content.digital.nhs.uk/catalogue/PUB21189.

28. Hospital Episode Statistics. Admitted patient care England 2014-15: Procedures and interventions. Secondary Admitted patient care England 2014-15: Procedures and interventions 2015. http://www.hscic.gov.uk/catalogue/PUB19124/hosp-epis-stat-admi-proc-2014-15-tab.xlsx.

29. National Joint Registry for England and Wales. OPCS codes relevant procedure is recorded in the NJR. Secondary OPCS codes relevant procedure is recorded in the NJR 2013. http://www.njrcentre.org.uk/njrcentre/Portals/0/Documents/England/Data%20collection%20forms/OPCS%20Procedure%20codes%20relevant%20to%20NJR.pdf.

30. National Joint Registry for England and Wales. 13th Annual Report. Secondary 13th Annual Report 2016. www.njrcentre.org.uk.

31. NHS Employers. Agenda for change pay bands and points from 1 April 2014. Secondary Agenda for change pay bands and points from 1 April 2014 2014. http://www.nhsemployers.org/~/media/Employers/Documents/Pay%20and%20reward/AfC%20pay%20bands%20and%20points%20poster%202014.pdf.

32. Curtis L. Unit Costs of Health and Social Care 2014. Secondary Unit Costs of Health and Social Care 2014 2014. http://www.pssru.ac.uk/project-pages/unit-costs/2014/.

33. Department of Health. NHS reference costs 2013 to 2014 - National schedule of reference costs: the main schedule. Secondary NHS reference costs 2013 to 2014 - National schedule of reference costs: the main schedule 2014. https://www.gov.uk/government/publications/nhs-reference-costs-2013-to-2014.

For peer review only

Table 1 Impact of different OKS thresholds on patient numbers, costs and QALYs among the 172,192 patients with knee osteoarthritis attending the hub in England each year

	Current practice	Maximum OKS at which patients can be referred for surgical assessment					
		**18**	**24**	**31**	**35**	**40**	**43**
Number of attendances at the surgical outpatient visit	80,211	65,286	99,982	123,635	129,256	131,633	131,983
Number of arthroplasty procedures conducted (% change*)	24,063	19,586 (-6%)	29,995 (+8%)	37,090 (+17%)	38,777 (+19%)	39,490 (+20%)	39,595 (+20%)
Total cost over 10 years (thousands)	£1,098,213	£1,081,313	£1,108,836	£1,134,134	£1,142,073	£1,146,019	£1,146,678
Total QALYs over 10 years	564,744	566,658	585,674	594,802	596,910	597,778	597,856
Net health benefit (QALYs)	509,834	512,593	530,232	538,096	539,806	540,477	540,522

* Percentage change in the total number of arthroplasty procedures following a change to referral patterns at the hub. Equal to the difference in the number of procedures between the scenario in question and 'current practice', divided by the 76,617 knee replacements conducted in England each year.[28]

Table 2 Impact of different OHS thresholds on patient numbers, costs and QALYs among the 41,121 patients with hip osteoarthritis attending the hub in England each year

	Current practice	Maximum OHS at which patients can be referred for surgical assessment					
		**18**	**24**	**30**	**35**	**40**	**45**
Number of attendances at the surgical outpatient visit	17,793	18,036	25,420	29,871	31,561	32,108	32,216
Number of arthroplasty procedures conducted (% change*)	6,590	6,680 (+0%)	9,415 (+4%)	11,063 (+6%)	11,689 (+7%)	11,892 (+8%)	11,932 (+8%)
Total cost over 10 years (thousands)	£168,402	£168,202	£182,080	£190,519	£193,753	£194,816	£195,030
Total QALYs over 10 years	123,674	128,990	135,863	138,888	139,929	140,248	140,288
Net health benefit (QALYs)	115,254	120,580	126,759	129,362	130,242	130,508	130,537

* Percentage change in the total number of arthroplasty procedures following a change to referral patterns at the hub. Equal to the difference in the number of procedures between the scenario in question and 'current practice', divided by the 69,313 hip replacements conducted in England each year.[28]

For peer review only - http://bmjopen.bmj.com/site/about/guidelines.xhtml

Figure 1 Patient flow diagram for patients referred with knee symptoms

* See Table A2 in online supplementary appendix for a list of the conditions other than osteoarthritis for which patients aged ≥50 years were excluded from the analysis.

For peer review only

For peer review only - http://bmjopen.bmj.com/site/about/guidelines.xhtml

Figure 2 Patient flow diagram for patients referred with hip symptoms

* See Table A2 in online supplementary appendix for a list of the conditions other than osteoarthritis for which patients aged ≥50 years were excluded from the analysis.

For peer review only

For peer review only - http://bmjopen.bmj.com/site/about/guidelines.xhtml

Figure 3 Number of patients predicted to be referred with (A) knee and (B) hip osteoarthritis symptoms in England

For peer review only

For peer review only - http://bmjopen.bmj.com/site/about/guidelines.xhtml

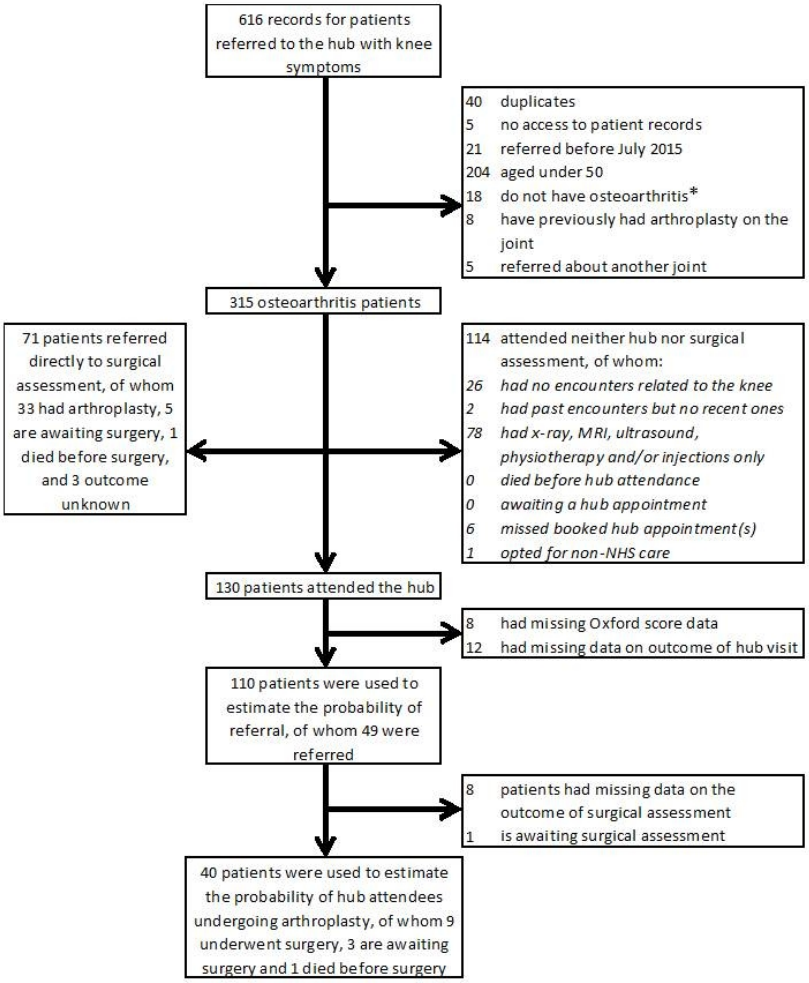

Figure 1 Patient flow diagram for patients referred with knee symptoms.

* See Table A2 in online supplementary appendix for a list of the conditions other than osteoarthritis for which patients aged ≥50 years were excluded from the analysis.

190x275mm (96 x 96 DPI)

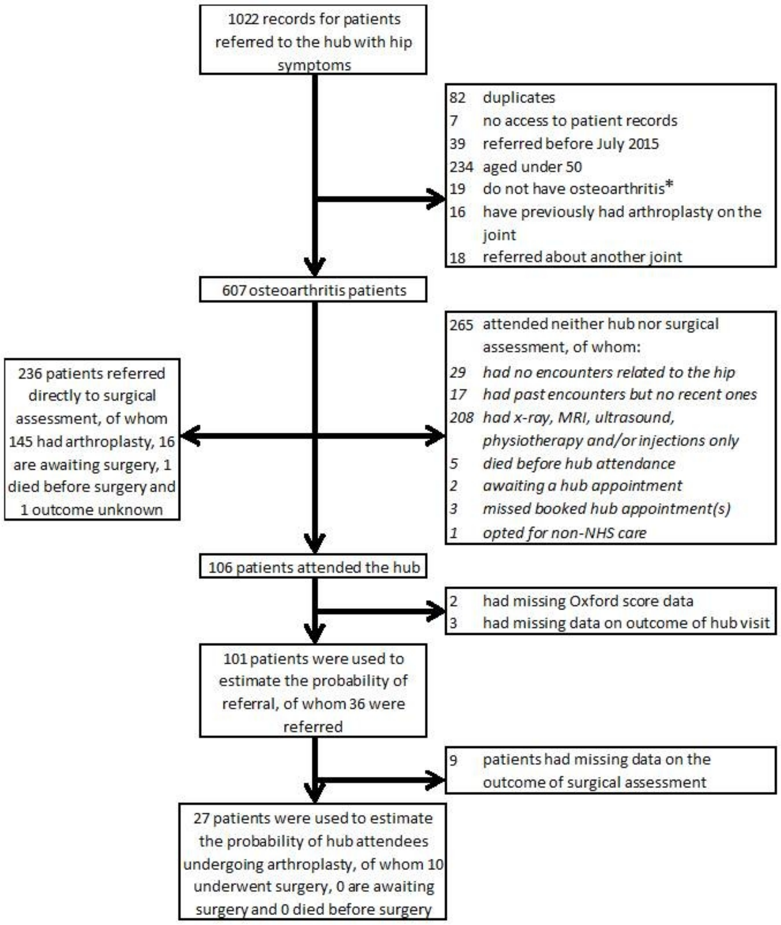

Figure 2 Patient flow diagram for patients referred with hip symptoms.

* See Table A2 in online supplementary appendix for a list of the conditions other than osteoarthritis for which patients aged ≥50 years were excluded from the analysis.

190x275mm (96 x 96 DPI)

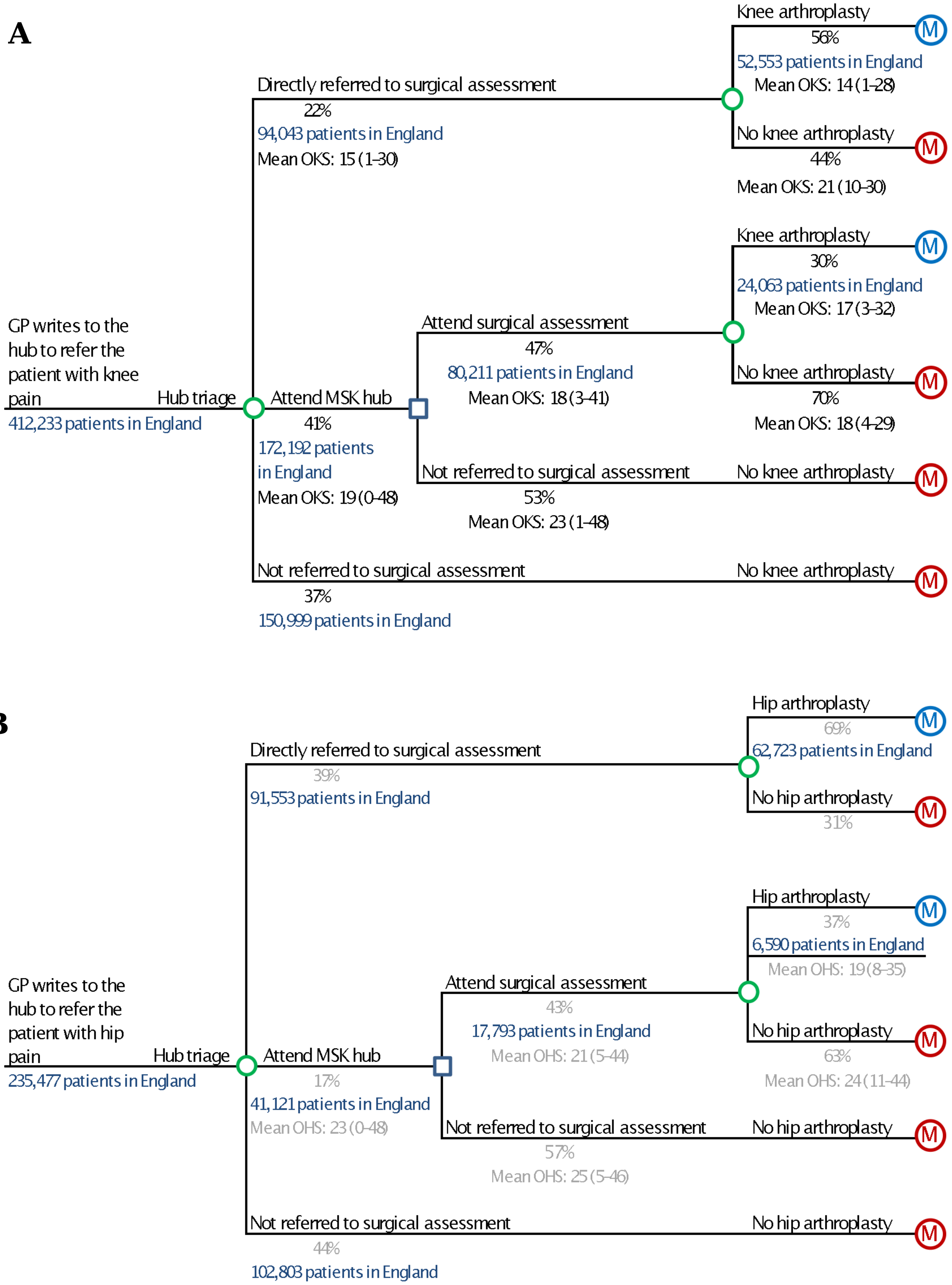
A
Knee arthroplasty
56%
52,553 patients in England
Mean OKS: 14 (1-28)
Directly referred to surgical assessment
22%
94,043 patients in England
Mean OKS: 15 (1-30)
No knee arthroplasty
44%
Mean OKS: 21 (10-30)
Knee arthroplasty
30%
24,063 patients in England
Mean OKS: 17 (3-32)
GP writes to the
hub to refer the
patient with knee
pain
Hub triage
412,233 patients in England
Attend surgical assessment
47%
80,211 patients in England
Mean OKS: 18 (3-41)
No knee arthroplasty
70%
Mean OKS: 18 (4-29)
Attend MSK hub
41%
172,192 patients
in England
Mean OKS: 19 (0-48)
Not referred to surgical assessment
53%
Mean OKS: 23 (1-48)
No knee arthroplasty
Not referred to surgical assessment
37%
150,999 patients in England
No knee arthroplasty
M
B
Hip arthroplasty
69%
62,723 patients in England
Directly referred to surgical assessment
39%
91,553 patients in England
No hip arthroplasty
31%
Hip arthroplasty
37%
6,590 patients in England
Mean OHS: 19 (8-35)
Attend surgical assessment
43%
17,793 patients in England
Mean OHS: 21 (5-44)
GP writes to the
hub to refer the
patient with hip
pain
Hub triage
235,477 patients in England
Attend MSK hub
17%
41,121 patients in England
Mean OHS: 23 (0-48)
No hip arthroplasty
63%
Mean OHS: 24 (11-44)
Not referred to surgical assessment
57%
Mean OHS: 25 (5-46)
No hip arthroplasty
Not referred to surgical assessment
44%
102,803 patients in England
No hip arthroplasty

Online supplementary file

Methods for stepwise regression models

We selected variables for inclusion in the logistic regression model in a pre-specified sequence. We first estimated a model only including the constant term and evaluated whether adding OKS/OHS to the model reduced Akaike information criterion (AIC); if AIC was reduced, the model used in the next step controlled for OKS/OHS and otherwise remained with just a constant term. Similarly, the next step assessed whether adding age into the model reduced AIC and in the final step we assessed whether adding sex into the model reduced AIC. We specified the order in which variables were considered for the model (OKS/OHS, then age, then sex) in advance of data analysis: it was hypothesised that OKS/OHS would be the most important factor determining referrals, followed by age (since this is a proxy for comorbidities) and finally sex (since we had no prior hypotheses about how sex would affect referrals). Age and OKS/OHS were assumed to have linear effects on the log-odds of being referred for surgical assessment as there were insufficient data to evaluate nonlinear functions.

Methods for estimating patient numbers

Hub data were extrapolated to estimate patient numbers across England by applying the probability of referral estimated on the hub data to national data on the characteristics of patients undergoing arthroplasty and the total number of operations.

We considered 490 groups of patients, defined by gender, age (using age bands <55, 55-64, 65-74, 75-84 and ≥85) and individual preoperative OKS/OHS between 0 and 48. An extract of individual patient data from the national patient reported outcome measures (PROMs) dataset linked to Hospital Episode Statistics (HES) covering operations conducted between 2009 and 2015 was used to estimate the proportion of arthroplasty patients in different groups defined by age and gender. The same dataset was used to obtain the proportion of patients with each preoperative OKS/OHS.

The number of operations in each of the 490 patients groups was calculated by multiplying the proportion of patients in different age and sex groups by the proportion with different OKS/OHS and by the number of primary knee/hip replacements conducted solely for osteoarthritis in England in 2014-15. The total number of primary knee/hip replacements conducted for osteoarthritis (76,617 knee replacements and 69,313 hip replacements) was calculated by multiplying the total number of finished consultant episodes in HES[28] with the OPCS codes beginning with O or W that the National joint Registry (NJ) used to identify primary hip/knee replacement[29] (79,726 knee replacements and 77,880 hip replacements) by the proportion of primary arthroplasty procedures that are conducted solely for osteoarthritis (96% for knees and 89% for hips).[30]

For peer review only - http://bmjopen.bmj.com/site/about/guidelines.xhtml

We then calculated the number of patients in each group who are likely to have been referred by GPs with knee/hip symptoms by dividing the number of operations in each group by the probability that each group of patients will undergo surgery.

Unit costs

The unit costs used in the current analysis are shown in Table A1. The costs and QALYs accrued in the 10-year period after arthroplasty (or a decision not to perform arthroplasty) have been reported previously.[5]

Table A1 Unit costs

Healthcare resource	Cost per consultation	Reference and details
Attendance at musculoskeletal hub	£58	Based on 40 minutes' of patient-related activity for a grade 8a physiotherapist. Based on activity at the NOC hub, each hub attendance lasts 30 minutes, plus an additional 10 minutes' administration per patient and will, on average, be conducted by a grade 8a physiotherapist who spends around 80% of their time on patient-related activity. The salary for a band 8a, point 36 physiotherapist was based on Agenda for Change salaries[31] and converted to an hourly rate by applying the same methodology as was applied by PSSRU for a hospital physiotherapist,[32] assuming that working hours and the percentages for salary on costs, qualifications and overheads would be the same for all hospital physiotherapists.
Outpatient consultation with an orthopaedic surgeon	£132	National average unit cost for first consultant-led non-admitted face-to-face attendance, WF01B, CL sheet of the National Schedule of Reference Costs Year 2013-14 NHS trusts and NHS foundation trusts[33]

For peer review only

Additional results

Table A2 Conditions other than osteoarthritis for which patients aged ≥50 years were excluded from the analysis

	Knee audit	Hip audit
Total patients aged ≥50 excluded due to conditions other than osteoarthritis	18	19
Rheumatoid arthritis	5	9
Bone or joint infections	4	2
Requiring limb reconstruction	1	2
Injury	2	1
No mechanical symptoms	1	1
Fracture	0	3
Arthritis secondary to fracture	2	0
Symptoms caused by previous spinal surgery	0	1
Gout	1	0
Chondroid lesion	1	0
Quadriceps rupture	1	0

Table A3 Characteristics of the patients included in the hub audit

	Number of patients	Mean age (range)	% Female (n/N)	Mean OKS/OHS (range; N)
Knee patients				
All osteoarthritis patients	315	66 (50, 93)	52% (165/315)	-
All patients referred directly to surgical assessment	71	68 (51, 86)	61% (43/71)	15 (1, 30; N=27)
Patients not referred for arthroplasty following direct referral to surgical assessment	*30*	*63 (51, 86)*	*53% (16/30)*	*21 (10, 30; N=5)*
Patients referred for arthroplasty following direct referral to surgical assessment	*38*	*72 (55, 86)*	*63% (24/38)*	*14 (1, 28; N=22)*
Patients attending neither hub nor surgical assessment	114	66 (50, 86)	49% (56/114)	-
All patients attending the hub	130	65 (50, 93)	51% (66/130)	19 (0, 48; N=130)
Patients not referred to surgical assessment following hub attendance	*61*	*64 (50, 88)*	*44% (27/61)*	*23 (1, 48; N=61)*
Patients referred to surgical assessment following hub attendance	*49*	*65 (51, 88)*	*49% (24/49)*	*18 (3, 41; N=49)*
Patients referred for arthroplasty	*12*	*66 (55, 88)*	*33% (4/12)*	*17 (3, 32;*

For peer review only

	Number of patients	Mean age (range)	% Female (n/N)	Mean OKS/OHS (range; N)
following hub attendance and surgical assessment				*N=12)*
Patients not referred for arthroplasty following hub attendance and surgical assessment	*28*	*65 (51, 82)*	*57% (16/28)*	*18 (4, 29; N=28)*
Hip patients				
All osteoarthritis patients	607	70 (50, 95)	67% (407/607)	-
All patients referred directly to surgical assessment	236	72 (50, 95)	67% (158/236)	-
Patients not referred for arthroplasty following direct referral to surgical assessment	*74*	*71 (51, 95)*	*80% (59/74)*	-
Patients referred for arthroplasty following direct referral to surgical assessment	*161*	*72 (50, 92)*	*61% (98/161)*	-
Patients attending neither hub nor surgical assessment	265	70 (50, 93)	64% (170/265)	-
All patients attending the hub	106	67 (50, 89)	75% (79/106)	23 (0, 48; N=106)
Patients not referred to surgical assessment following hub attendance	*65*	*67 (51, 89)*	*80% (52/65)*	*25 (5, 46; N=65)*
Patients referred to surgical assessment following hub attendance	*36*	*67 (50, 85)*	*67% (24/36)*	*21 (5, 44; N=36)*
Patients referred for arthroplasty following hub attendance and surgical assessment	*10*	*72 (56, 85)*	*50% (5/10)*	*19 (8, 35; N=10)*
Patients not referred for arthroplasty following hub attendance and surgical assessment	*17*	*67 (51, 84)*	*65% (11/17)*	*24 (11, 44; N=17)*

For peer review only

FIGURE A1 Distribution of (**A**) OKS and (**B**) OHS for patients attending the hub with knee/hip pain compared with the distribution of patients undergoing knee arthroplasty in England in PROMs/HES (2009-2015)

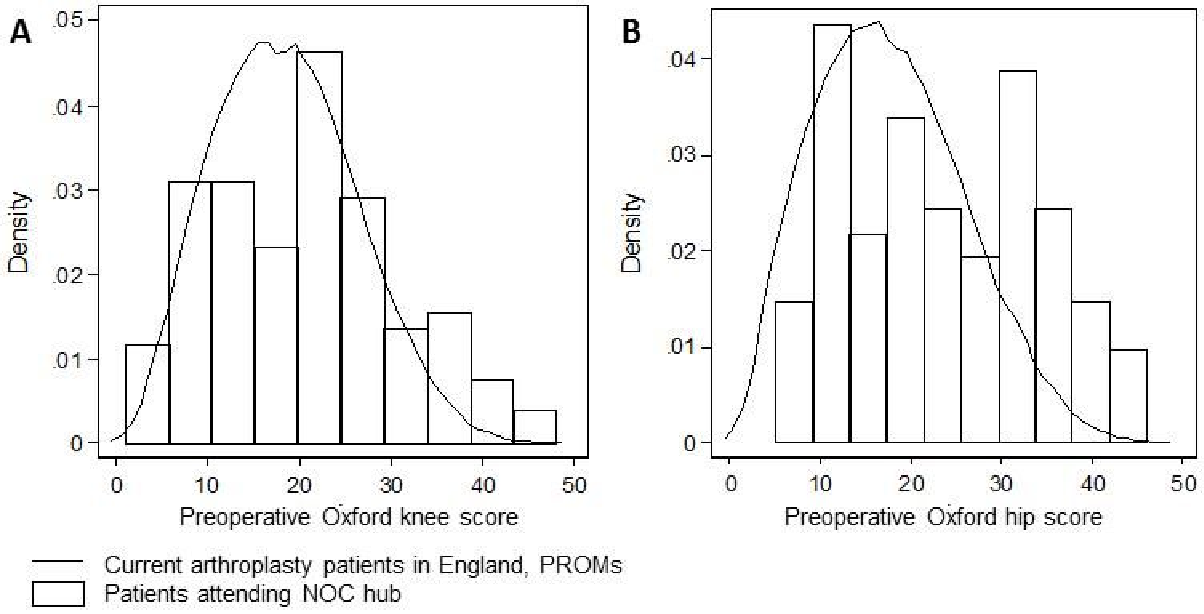

Additional regression results

For patients attending the hub with knee symptoms, adding age and sex into the model predicting the odds of hub attendees being referred for surgical assessment increased AIC, so the final model included only OKS (Table A4). Similarly, in the model of hip patients, adding age into the model worsened AIC, so the final model included only OHS and gender. Omitting gender from the model had negligible effect on the coefficient for OHS, which was 0.0397 (SE: 0.0211) when OHS was the only explanatory variable.

TABLE A4 Results of logistic regression predicting the odds of patients attending the hub being referred for surgical assessment

Variable	Mean coefficient (SE)	
	Knee patients (N=110)	Hip patients (N=101)
OKS	-0.048 (0.021)*	-0.0397 (0.0213)
Male	-	0.6965 (0.4804)
Constant	0.768 (0.459)	0.1428 (0.5376)
Pseudo-R^2	0.0389	0.0441

* p<0.05

In addition to the logistic regression model predicting the odds of patients being referred from the hub to surgical assessment, we also conducted a secondary analysis predicting the odds of undergoing arthroplasty in patients who had already been referred to surgical assessment by the hub, using the same methods. This analysis suggested that OKS/OHS, age and sex had no significant effect on the odds of referral from surgical assessment to surgery

(p>0.05) and that including any of these variables in the regression increased AIC compared with a model with only a constant term. This validates the assumption that the probability of patients referred directly to surgical assessment subsequently being referred for surgery is independent of OKS/OHS, age and sex and the assumption that introducing OKS/OHS thresholds at the hub would have no effect on the probability of a patient with particular preoperative characteristics being referred for arthroplasty following a surgical assessment visit.

For peer review only

STROBE 2007 (v4) Statement—Checklist of items that should be included in reports of *cross-sectional studies*

Section/Topic	Item #	Recommendation	Section where reported
**Title and abstract**	1	(*a*) Indicate the study's design with a commonly used term in the title or the abstract	Title
		(*b*) Provide in the abstract an informative and balanced summary of what was done and what was found	Title
**Introduction**			
Background/rationale	2	Explain the scientific background and rationale for the investigation being reported	Introduction
Objectives	3	State specific objectives, including any prespecified hypotheses	Introduction
**Methods**			
Study design	4	Present key elements of study design early in the paper	Strengths and limitations
Setting	5	Describe the setting, locations, and relevant dates, including periods of recruitment, exposure, follow-up, and data collection	Methods page 6
Participants	6	(*a*) Give the eligibility criteria, and the sources and methods of selection of participants	Methods page 6
Variables	7	Clearly define all outcomes, exposures, predictors, potential confounders, and effect modifiers. Give diagnostic criteria, if applicable	Methods page 6
Data sources/ measurement	8*	For each variable of interest, give sources of data and details of methods of assessment (measurement). Describe comparability of assessment methods if there is more than one group	Methods page 6
Bias	9	Describe any efforts to address potential sources of bias	*N/A*
Study size	10	Explain how the study size was arrived at	Methods page 6
Quantitative variables	11	Explain how quantitative variables were handled in the analyses. If applicable, describe which groupings were chosen and why	Methods page 6-7
Statistical methods	12	(*a*) Describe all statistical methods, including those used to control for confounding	Methods pages 6-7 and online supplementary appendix
		(*b*) Describe any methods used to examine subgroups and interactions	N/A
		(*c*) Explain how missing data were addressed	Methods page7
		(*d*) If applicable, describe analytical methods taking account of sampling strategy	N/A

For peer review only

		(*e*) Describe any sensitivity analyses	N/A
**Results**			
Participants	13*	(a) Report numbers of individuals at each stage of study—eg numbers potentially eligible, examined for eligibility, confirmed eligible, included in the study, completing follow-up, and analysed	Figures 1-2
		(b) Give reasons for non-participation at each stage	Figures 1-2 and Table A2
		(c) Consider use of a flow diagram	Figures 1-2
Descriptive data	14*	(a) Give characteristics of study participants (eg demographic, clinical, social) and information on exposures and potential confounders	Table A3, Figure A1
		(b) Indicate number of participants with missing data for each variable of interest	Figures 1-2 and Table A3
Outcome data	15*	Report numbers of outcome events or summary measures	Figures 1-3
Main results	16	(*a*) Give unadjusted estimates and, if applicable, confounder-adjusted estimates and their precision (eg, 95% confidence interval). Make clear which confounders were adjusted for and why they were included	Online supplementary appendix
		(*b*) Report category boundaries when continuous variables were categorized	N/A
		(*c*) If relevant, consider translating estimates of relative risk into absolute risk for a meaningful time period	N/A
Other analyses	17	Report other analyses done—eg analyses of subgroups and interactions, and sensitivity analyses	Methods pages 6-8 and online supplementary file
**Discussion**			
Key results	18	Summarise key results with reference to study objectives	Discussion, paragraph 1
Limitations	19	Discuss limitations of the study, taking into account sources of potential bias or imprecision. Discuss both direction and magnitude of any potential bias	Discussion, paragraphs 2-3
Interpretation	20	Give a cautious overall interpretation of results considering objectives, limitations, multiplicity of analyses, results from similar studies, and other relevant evidence	Discussion
Generalisability	21	Discuss the generalisability (external validity) of the study results	Discussion, paragraph 5
**Other information**			
Funding	22	Give the source of funding and the role of the funders for the present study and, if applicable, for the original study on which the present article is based	Funding and role of study sponsors

*Give information separately for cases and controls in case-control studies and, if applicable, for exposed and unexposed groups in cohort and cross-sectional studies.

Note: An Explanation and Elaboration article discusses each checklist item and gives methodological background and published examples of transparent reporting. The STROBE checklist is best used in conjunction with this article (freely available on the Web sites of PLoS Medicine at http://www.plosmedicine.org/, Annals of Internal Medicine at http://www.annals.org/, and Epidemiology at http://www.epidem.com/). Information on the STROBE Initiative is available at www.strobe-statement.org.

For peer review only

BMJ Open

Who gets referred for knee or hip replacement? A theoretical model of the potential impact of evidence-based referral thresholds using data from a retrospective review of clinic records from an English musculoskeletal referral hub

Journal:	*BMJ Open*
Manuscript ID	bmjopen-2019-028915.R1
Article Type:	Original research
Date Submitted by the Author:	26-Jun-2019
Complete List of Authors:	Dakin, Helen; Oxford University, Health Economics Research Centre, Nuffield Department of Population Health<br>Eibich, Peter; Max-Planck-Institut fur Demografische Forschung; Oxford University, Health Economics Research Centre, Nuffield Department of Population Health<br>Gray, Alastair; Oxford University, Health Economics Research Centre, Nuffield Department of Population Health<br>Smith, James; Oxford University, Nuffield Department of Orthopaedics, Rheumatology and Musculoskeletal Sciences; Macquarie University, Centre for Healthcare Resilience and Implementation Science, Australian Institute of Health Innovation<br>Barker, Karen L.; Oxford University, Nuffield Department of Orthopaedics, Rheumatology and Musculoskeletal Sciences<br>Beard, David; Oxford University, Nuffield Department of Orthopaedics, Rheumatology and Musculoskeletal Sciences<br>Price, Andrew; Oxford University, Nuffield Department of Orthopaedics, Rheumatology and Musculoskeletal Sciences
**Primary Subject Heading**:	Surgery
Secondary Subject Heading:	General practice / Family practice, Health economics, Health policy, Rheumatology
Keywords:	Arthroplasty, hip replacement, knee replacement, prioritisation, osteoarthritis

SCHOLARONE™
Manuscripts

Who gets referred for knee or hip replacement? A theoretical model of the potential impact of evidence-based referral thresholds using data from a retrospective review of clinic records from an English musculoskeletal referral hub

Helen Dakin,[1] Peter Eibich,[1,2] Alastair Gray,[1] James Smith,[3,4] Karen Barker,[3] David Beard,[3] Andrew Price[3] on behalf of the ACHE Study team

[1] Health Economics Research Centre, Nuffield Department of Population Health, University of Oxford, UK

[2] Max Planck Institute for Demographic Research, Rostock, Germany

[3] Nuffield Department of Orthopaedics, Rheumatology, and Musculoskeletal Science, NIHR Biomedical Research Unit, University of Oxford, Oxford, UK

[4] Centre for Healthcare Resilience and Implementation Science, Macquarie University, Australia

Contributors: Sujin Kang, Jonathan Cook, Ray Fitzpatrick, David Murray, Nigel Arden, Andy Carr, Stephanie Smith, Kristina Harris, Rob Middleton, Elizabeth Gibbons, Elena Benedetto, Jill Dawson, Adrian Sayers, Laura Miller, Elsa Marques, Rachael Gooberman-Hill, Ashley Blom, Andrew Judge, and Sion Glyn-Jones.

Short title for running header: Who gets referred for joint replacement?

Contact details for authors:

Helen Dakin [corresponding author]: Senior Researcher, Health Economics Research Centre, Nuffield Department of Population Health, University of Oxford, Old Road Campus, Headington, Oxford, OX3 7LF, UK. Email: helen.dakin@ndph.ox.ac.uk; Tel: 01865 289422.

Peter Eibich: Senior Researcher, Health Economics Research Centre, Nuffield Department of Population Health, University of Oxford, Old Road Campus, Headington, Oxford, OX3 7LF, UK. Research Scientist, Max Planck Institute for Demographic Research, Konrad-Zuse-Str. 1, 18057 Rostock, Germany. Email: eibich@demogr.mpg.de.

Alastair Gray: Professor of Health Economics, Health Economics Research Centre, Nuffield Department of Population Health, University of Oxford, Old Road Campus, Headington, Oxford, OX3 7LF, United Kingdom. Email: alastair.gray@ndph.ox.ac.uk.

James Smith: Research Fellow, Nuffield Department of Orthopaedics, Rheumatology and Musculoskeletal Sciences, University of Oxford, Botnar Research Centre, Windmill Road, Headington, Oxford, OX3 7LD, UK. Centre for Healthcare Resilience and Implementation Science, Australian Institute of Health Innovation, Macquarie University, NSW 2109, Australia. Email: jim.smith@mq.edu.au.

For peer review only

Karen Barker: Clinical Director, Nuffield Orthopaedic Centre, Oxford University Hospitals Trust, Oxford, OX3 7HE, UK; Professor of Physiotherapy, Nuffield Department of Orthopaedics, Rheumatology and Musculoskeletal Sciences, University of Oxford, Botnar Research Centre, Windmill Road, Headington, Oxford, OX3 7LD, UK. Email: Karen.Barker@ouh.nhs.uk.

David Beard: Professor of Musculoskeletal and Surgical Science, Nuffield Department of Orthopaedics, Rheumatology and Musculoskeletal Sciences, University of Oxford, Botnar Research Centre, Windmill Road, Headington, Oxford, OX3 7LD, UK. Email: david.beard@ndorms.ox.ac.uk.

Andrew Price [guarantor]: Professor of Orthopaedic Surgery, Nuffield Department of Orthopaedics, Rheumatology and Musculoskeletal Sciences, University of Oxford, Botnar Research Centre, Windmill Road, Headington, Oxford, OX3 7LD, UK. Email: andrew.price@ndorms.ox.ac.uk.

Institution where the work was carried out: Health Economics Research Centre and Nuffield Department of Orthopaedics, Rheumatology, and Musculoskeletal Science, University of Oxford

Word Count: 4224

Tables: 2

Figures: 3

For peer review only

Abstract

Objectives: To estimate the relationship between patient characteristics and referral decisions made by musculoskeletal hubs, and assess the possible impact of an evidence-based referral tool.

Design: Retrospective analysis of medical records and decision-tree model evaluating policy changes using local and national data.

Setting: One musculoskeletal interface clinic (hub) in England.

Participants: 922 adults aged ≥50 years referred by GPs with symptoms of knee or hip osteoarthritis.

Interventions: We assessed the current frequency and determinants of referrals from one hub and the change in referrals that would occur at this centre and nationally if thresholds for referral (Oxford knee and hip scores, OKS/OHS) were introduced.

Main outcome measure: OKS/OHS, referrals for surgical assessment, referrals for arthroplasty, costs and quality adjusted life-years (QALYs).

Results: Of 110 patients with knee symptoms attending face-to-face hub consultations, 49 (45%) were referred for surgical assessment; mean OKS for these 49 patients was 18 (range: 1-41). Among hip patients, 36% (36/101) were referred for surgical assessment (mean OHS: 21, range: 5-44). No patients referred for surgical assessment were above previously-reported economic thresholds for OKS (43) or OHS (45). Setting thresholds of OKS≤31 and OHS≤35 might have resulted in an additional 22 knee referrals and 26 hip referrals in our cohort. Extrapolating hub results across England suggests a possible increase in referrals nationally, of around 13,000 additional knee replacements and 4,500 additional hip replacements each year.

Conclusions: Musculoskeletal hubs currently consider OKS/OHS and other factors when making decisions about referral to secondary care for joint replacement. Those referred for surgery typically have low OHS/OKS scores and introducing evidence-based OKS/OHS thresholds would prevent few inappropriate (high-functioning, low-pain) referrals. However, our findings suggest that some patients not currently referred could benefit from arthroplasty based on OKS/OHS. More research is required to explore other important patient characteristics currently influencing hub decisions.

Keywords

Arthroplasty; hip replacement; knee replacement; prioritisation; osteoarthritis.

For peer review only

Strengths and limitations of this study

- This is the first analysis reporting data on the characteristics of patients being referred to a UK hub with symptoms of hip or knee osteoarthritis; such data are needed to assess the impact of changing referral criteria on the number of knee/hip replacements and on costs and quality of life.
- We retrospectively reviewed clinical records for 922 men and women referred to a single musculoskeletal hub, although only 221 patients underwent arthroplasty.
- We used several assumptions to extrapolate our estimates of how the probability of referral varies with preoperative characteristics using a decision-tree and Markov model, which enabled us to estimate the potential impact of different referral policies nationally.
- A prospective pilot study would be required to quantify the real-world impact of any policy change, while data from multiple hubs and information on other patient characteristics (such as BMI) are required for a comprehensive understanding of current clinical practice.

For peer review only

Introduction

Recently, many clinical commissioning groups (CCGs) in the UK have set referral criteria for joint replacement that exclude patients with Oxford knee and hip scores (OKS and OHS) above certain thresholds, which are often as low as 19[1] or 24[1-3] out of 48. Recent papers have shown that these low thresholds are inappropriate: arthroplasty has a ≥80% chance of producing a meaningful improvement in patients with OKS/OHS of 19 or 24[4] and would cost <£10,000 per quality adjusted life-year (QALY).[5] The recent ACHE study analysed >400,000 medical records and concluded that thresholds between 31 and 41 may be justified on clinical and cost-effectiveness grounds.[4 5]

Commissioners' decisions concerning thresholds may be influenced by the likely number of operations and their cost. In the UK, >94% of patients currently undergoing arthroplasty have OKS/OHS ≤31, demonstrating that nearly all operations currently conducted were cost-effective and had ≥70% chance of meaningful improvement.[4 5] However, there are very little data on how patient characteristics influence the assessment process in practice, or on patients who do not currently receive surgery, making it difficult to assess the impact that different referral criteria may have on patient numbers or costs. As in many other clinical areas, hubs (also known as triage clinics, musculoskeletal interface services or intermediate musculoskeletal assessment centres) are increasingly used as gatekeepers determining access to consultations with orthopaedic surgeons, assessing all patients being considered for surgery.[6-8]

We aimed to review the characteristics of patients attending hub consultations, identify how patients within a hub are currently managed and explore how referral decisions vary with preoperative characteristics. To illustrate how such data could be used to inform policy, we estimated the potential impact of a policy change by which patients are referred from a hub based on evidence-based OKS/OHS thresholds.

Methods

Outline

We collected data from one UK referral hub and used it to estimate a logistic regression model predicting the probability that patients will be referred from the hub to surgical assessment based on their OKS/OHS, age and sex. We then applied this regression model to nationally-collected preoperative outcomes data[9-14] to estimate how many patients are referred for surgical assessment nationally. We predicted how many of these patients would be referred through to secondary care if different OKS/OHS thresholds were introduced and potentially how many extra operations would be performed.

For peer review only - http://bmjopen.bmj.com/site/about/guidelines.xhtml

Hub data collection

Anonymised data were extracted retrospectively from medical records of patients with knee or hip symptoms who had been referred by GPs between July 2015 and July 2016 to the musculoskeletal hub at the Nuffield Orthopaedic Centre (NOC) in Oxford. This study discussed and agreed through the Clinical Governance Group for hip and knee replacement as part of a wider review of outcomes in hip and knee arthroplasty (Oxford University Hospitals Research and Development Ref 11603). The primary statistical analysis used a prognostic model to estimate how the probability of patients attending face-to-face consultations at the hub being referred to surgical assessments in secondary care varied with OKS/OHS, age and sex. We therefore chose the sample size to provide data on ≥30 knee referrals and ≥30 hip referrals from the hub to secondary care, thereby providing ≥10 events per explanatory variable.[15]

Two medically-qualified surgical research fellows extracted the following: age; sex; OKS/OHS at hub attendance; attendance date; whether the patient was referred to secondary care; date of any subsequent surgical assessment visit; and date of any subsequent arthroplasty surgery. Additional information on imaging, referrals to other clinics, and other surgeries or diagnoses was recorded in free text fields. Data on OKS at the surgical assessment visit were also extracted, when available, from the secondary care records of patients with knee pain. Body mass index (BMI) was not extracted as the analysis focused on OKS/OHS and the available evidence on how capacity to benefit and cost-effectiveness vary with OKS/OHS does not consider BMI.[4 5] Patients who were on the waiting list for arthroplasty surgery at the time of data extraction (August 2016) and those for whom surgery was delayed due to comorbidities or high BMI after their attendance at the surgical assessment consultation were counted as having been referred for surgery.

Exclusion criteria comprised:

- Aged <50 years (for whom knee/hip pain is unlikely to be caused by osteoarthritis).
- Evidence from medical records that symptoms were due to a condition other than osteoarthritis.
- Previous arthroplasty on the same joint.
- Medical records inaccessible for research.
- Any attendance at the hub or surgical assessment unit before July 2015.

However, patients who were referred for x-rays, magnetic resonance imaging (MRI) or physiotherapy but did not attend face-to-face consultations at the hub or surgical assessment unit were included in the descriptive analysis.

Statistical modelling

Logistic regression was used to estimate a prognostic model of the local hub data predicting how OKS/OHS, age and sex affected the odds of being referred for surgical assessment

For peer review only - http://bmjopen.bmj.com/site/about/guidelines.xhtml

following a face-to-face attendance at the hub. Age and OKS/OHS at hub attendance were analysed as continuous variables. Explanatory variables were selected based on the Akaike information criterion using forward stepwise regression, manually testing variables in a pre-specified sequence (online supplementary appendix). Analyses were conducted in Stata version 14 (StataCorp, College Station TX) on a complete case basis, excluding patients with missing data. We analysed binary variables using two-sample tests of proportions and continuous variables using unpaired t-tests preceded by F-tests for equal variance. OKS/OHS in the hub sample was compared against population means[9-14] using one-sample t-tests.

Estimating the number of referrals and effect of introducing thresholds

A decision-tree model of the treatment pathway was developed based on hub data and the authors' clinical experience of running hubs and/or surgical clinics. Using the model, hub data were extrapolated to estimate the number of patients in England who are referred for hip or knee replacement (online supplementary appendix). The overall proportion of patients referred directly to surgical assessment and the proportion attending face-to-face consultations at the hub were calculated from the hub sample. We also estimated the proportion of patients who underwent arthroplasty after being referred from the hub to surgical assessment. Logistic regression results were used to predict the probability of being referred from the hub to surgical assessment for men and women aged 50, 60, 70, 80 and 90 with different OKS/OHS. For each patient group defined by age, sex and OKS/OHS, we then estimated the number of patients referred to hubs nationally by dividing national data on the number of joint replacements currently conducted for osteoarthritis in England[9-14] by the probability that patients in each group would undergo surgery.

We then estimated how the number of referrals to surgical assessment and the number of arthroplasty procedures might change if different OKS/OHS thresholds between 18 and 45 were used to determine referral decisions during face-to-face hub consultations.

This analysis made the following assumptions:

1) Introducing OKS/OHS thresholds at the hub was assumed to have no effect on GP referrals, the hub triage or the probability of a patient with particular preoperative characteristics being referred for arthroplasty following a surgical assessment visit.
2) Since patient records provided no data on OKS/OHS for patients who did not attend the hub, we assumed that the probability of direct referral to surgical assessment or the probability of attending a face-to-face visit at the hub were independent of OKS/OHS, age and sex. Although symptom severity is one of the main factors considered in the hub triage, this assumption is unlikely to affect estimates of the impact of changing referral criteria applied to those patients attending the hub.
3) We also assumed that the probability of patients referred to surgical assessment subsequently being referred for surgery was independent of OKS/OHS, age and sex,

For peer review only

which was supported by a secondary regression analysis (online supplementary material).

4) We assumed that the referral probabilities estimated from the NOC hub sample are broadly representative of clinical practice across the UK.
5) We assumed, based on the experience of clinical co-authors, that 50% of patients who are currently not referred for surgical assessment after a face-to-face hub visit would still not be considered candidates for arthroplasty (and therefore not referred) regardless of whether thresholds were introduced. This group of patients includes patients who choose not to be referred, those who have not had a complete trial of non-operative treatment and others who are unsuitable for surgery due to comorbidities or other factors. The remaining 50% of patients were assumed to be referred only if their OKS/OHS was below the threshold.
6) Since there are no data on how OKS/OHS changes over time in the absence of arthroplasty, we assumed for simplicity patients who do not undergo arthroplasty following their hub attendance would not be referred back to clinic for 10 years.
7) Each 40-minute hub attendance was estimated to cost £58, and surgical assessment £132 (online supplementary appendix, Table A1).
8) The analyses took the perspective of the NHS, focusing on costs related to hip/knee arthroplasty. Costs of GP consultations, hub triage, x-rays, imaging, physiotherapy, injections, weight loss programs, missed appointments and referrals to other clinics were excluded as there is no reason to expect the proportion of patients requiring these services to change following the introduction of OKS/OHS thresholds.
9) The reference year for costs was 2014.

The patient numbers calculated within the decision-tree were used to estimate the cost of the referral pathway. The total 10-year costs and total QALYs estimated in a related study[5] for different patient characteristics with and without arthroplasty were applied to the number of patients expected to undergo arthroplasty or have no arthroplasty in each scenario. These figures were used to estimate the net health benefit for each scenario[16 17] (assuming a £20,000/QALY ceiling ratio) and the incremental cost-effectiveness of different pairs of scenarios (online supplementary appendix).

We also estimated the number of additional referrals that might have been observed within the population of individuals attending the NOC hub if a fixed OKS/OHS threshold were introduced. Based on assumption (4), this was equal to half the number of patients who had OKS/OHS at or below the proposed threshold but were not referred, minus the number who were referred with OKS/OHS above the proposed threshold.

Public and patient involvement

Patient representatives were involved in the grant application and design of the wider ACHE study.[4] Results of this part of the study were discussed with a user group including

For peer review only - http://bmjopen.bmj.com/site/about/guidelines.xhtml

clinicians and patient representatives; this discussion helped inform the interpretation and presentation of results.

Results

Current referral pathway

From the 1,638 records reviewed, we identified 315 patients with knee osteoarthritis and 607 with hip osteoarthritis: 922 in total (Figures 1, 2 and A1, Tables A2-A3).

During the time period for which data were collected, the musculoskeletal hub service was the only route for patients in Oxfordshire to access NHS elective orthopaedic surgery. When GPs refer patients to secondary care with knee/hip symptoms, the referral letter and x-rays are first reviewed by senior hub staff, who decide whether the patient should (a) be referred directly to surgical assessment, (b) attend the hub clinic (c) be managed in primary care (Figure 3). Triage is based on patient symptoms as described in the GP's referral letter (which will include BMI and may include OKS/OHS) and conservative treatments already given (such as anti-inflammatory drugs, physiotherapy and weight loss strategies).

Of the osteoarthritis patients included in the analysis, 23% (71/315) of knee patients and 39% (236/607) of hip patients were referred directly to surgical assessment based on the hub triage. In the authors' experience, these patients tend to be those with severe symptoms, those who had already exhausted all conservative measures and those who had previously been referred for surgical assessments, but had chosen not to have surgery at that time. Among 27 patients referred directly to surgical assessment for whom OKS data were available, the mean OKS was 15 (range: 1-30); no such data were available for hip patients.

At the surgical assessment visit, patients discuss the risks and benefits of surgery with an orthopaedic surgeon and make an informed decision about whether or not to undergo arthroplasty or other surgery, taking into account their comorbidities, symptom severity and other factors. Among the patients who were referred directly for surgical assessment and had outcomes data available, 56% (38/68) of knee patients and 69% (161/235) hip patients underwent or were awaiting knee/hip replacements at the time of data extraction.

Following triage, 36% (114/315) of knee patients and 44% (265/607) hip patients did not attend face-to-face consultations at either the hub or surgical assessment. Typically such patients comprise those who can be managed in primary care: for example those with mild symptoms and those who have not yet exhausted conservative treatment options, such as advice and information, activity and exercise and weight loss.[18-20] Across the 379 hip and

For peer review only - http://bmjopen.bmj.com/site/about/guidelines.xhtml

knee patients without face-to-face consultations, 270 (71%) had x-rays, MRI or ultrasound to identify the most appropriate care pathway. Eleven (3%) were referred for physiotherapy, while seven patients (2%), all with hip osteoarthritis, had injections. Other patients may not be seen face-to-face as they are unfit for surgery or have recent injuries likely to heal without further intervention. Eighteen patients opted for non-NHS care, missed/failed to book hub appointments, died before the hub attendance or were still awaiting a hub appointment at the time of data extraction (Figures 1 and 2).

The remaining 44% (130/315) of knee patients and 17% (106/607) of hip patients attended face-to-face assessments at the musculoskeletal hub, where extended-scope specialist physiotherapists or orthopaedic fellows assess patients to confirm diagnosis. Patients routinely complete OKS/OHS questionnaires to assess whether the level of symptoms warrants surgery and to guide discussions about the symptom profile. Among the patients attending the hub, the mean OKS was 21 (range: 1-48, Table A3, Figure A1), while mean OHS was 24 (range: 5-46); both values were significantly higher than the average for patients undergoing arthroplasty nationally (mean: 18; $p \leq 0.015$), although only OHS was significantly different from the average for patients undergoing arthroplasty in Oxfordshire (mean OKS: 20, $p=0.134$; mean OHS: 19 $p<0.0001$).[9-14]

Diagnostic imaging, landmark injections and injections for trochanteric bursitis may be done during the hub consultation. Patients with BMI ≥40 are referred for monitored weight loss programs that must be followed for 12 months before considering surgery. Staff and patients also discuss the risks and benefits of joint replacement (including recovery times, the need for support at home after hospital discharge and the potential need for revision surgery) and how these may be affected by patients' living arrangements and comorbidities. Following the hub visit, 45% (49/130) of knee patients and 36% (36/101) of hip patients were referred for surgical assessment. This includes patients who are considered candidates for arthroplasty or other procedures (e.g. arthroscopy, anterior cruciate ligament repair or interventional radiology).

Patients with higher OKS/OHS were significantly less likely to be referred for surgical assessment: each one-point increase in OKS reduced the odds of referral by 4.7% ($p=0.019$), while a one-point increase in OHS reduced the odds by 3.9% ($p=0.062$; Table A4). Those patients who are not referred to surgical assessment may be referred for physiotherapy, other non-surgical management or other outpatient clinics (e.g. rheumatology or sports injury clinics), or may choose not to be referred as they prefer not to undergo surgery at the current time.

Across the hub attendees who were referred for surgical assessment and had data on clinical outcomes, 30% (12/40) of knee patients and 37% (10/27) of hip patients underwent or were awaiting arthroplasty surgery.

For peer review only - http://bmjopen.bmj.com/site/about/guidelines.xhtml

Applying estimates of the probability of referral and subsequent arthroplasty from the hub dataset to national data on the distribution of patients undergoing knee/hip arthroplasty suggests that GPs in England refer around 417,000 patients with knee osteoarthritis and around 235,000 patients with hip osteoarthritis to secondary care each year (Figure 3). Of these, around 172,000 knee patients and 41,000 hip patients might attend a hub if the care pathway in Oxfordshire were followed nationally; such hub attendances would cost a total of £12 million.

Effect of introducing referral thresholds

We used the hub data to estimate the impact that different policies for using OKS/OHS to guide referral decisions might have on patient numbers, costs and health outcomes. The ACHE study reported that people with OKS ≤31 have a ≥70% chance of achieving a seven-point increase in OKS following knee arthroplasty.[4] Of the 110 knee patients attending the NOC hub, 94 (85%) had OKS ≤31, of whom 47 (50%) were referred for surgical assessment. Two patients were referred with OKS of 32 or 41. Assuming that 50% of the NOC patients with OKS ≤31 who are not currently referred might still choose not to be referred or might be considered clinically inappropriate for other reasons, a fixed referral threshold OKS of 31 would have resulted in an additional 22 referrals to surgical assessment: a 49% increase.

Applying the results of logistic regression to the whole population undergoing surgery nationally suggested that an OKS threshold of 31 would result in 43,000 additional surgical assessment visits and 13,000 additional knee replacement procedures in England each year (Table 1). Based on the costs and outcomes for patients with and without knee arthroplasty that were estimated in a recent economic evaluation,[5] introducing this policy would cost the NHS an additional £36 million for each annual cohort of patients (of which £5.8 million would be due to additional surgical assessment visits), but would gain 30,000 QALYs. This policy would be highly cost-effective compared with current practice, costing just £1,195 per QALY gained, and the net health benefit would be 28,000 QALYs even after subtracting the health benefits that would be foregone by spending £36 million on knee arthroplasty candidates, rather than other conditions. Introducing higher OKS thresholds would produce still greater health benefits and would continue to be cost-effective at a £20,000/QALY ceiling ratio right up to the economic threshold of 43.[5]

Similarly, ACHE predicts that people with preoperative OHS ≤35 have a ≥70% chance of achieving an eight-point increase in OHS following hip arthroplasty.[4] Within the NOC hub, 87% (88/101) of patients had OHS ≤35, of whom 35 were referred. One patient was referred with OHS of 44. Introducing a threshold of 35 might therefore result in 26 additional referrals: a 30% increase. Extrapolating across England, we might expect 14,000 additional surgical assessments and 5,000 additional hip replacements each year. The impact of this policy is lower than for knee osteoarthritis as 90% of hip replacements are done in patients

For peer review only - http://bmjopen.bmj.com/site/about/guidelines.xhtml

who were referred directly, and did not attend the hub. The policy would cost an additional £25 million (of which additional surgical assessments account for £2 million) and gain 16,000 QALYs, costing £1,560/QALY gained and generating a net health benefit of 15,000 QALYs. Introducing higher thresholds (up to the economic threshold of 45) would produce greater health benefits and continue to cost <£20,000/QALY gained.

Discussion

Although referral hubs are being used increasingly as gatekeepers determining access to consultations in different clinical areas of the NHS, there has been little research on them. This retrospective analysis characterised the patients being referred to and from a musculoskeletal hub, and showed that OKS/OHS and other factors were considered when deciding which patients to refer for surgical assessment. Since most patients attending hubs have relatively severe osteoarthritis symptoms and those with high OKS/OHS are less likely to be referred for surgery, introducing evidence-based OKS/OHS thresholds for arthroplasty is unlikely to prevent large numbers of inappropriate referrals. However, since recent evidence demonstrates that arthroplasty is both cost-effective and highly likely to benefit people with OKS/OHS well above the thresholds currently used by some CCGs,[4 5] such policies are also likely to identify patients who are not currently referred, but for whom arthroplasty could be expected to be beneficial and cost-effective. These numbers are uncertain, but extrapolations from the hub examined here suggest that, across England, setting thresholds of OKS ≤31 and OHS ≤35 would result in many more patients being referred for surgical assessment, and could result in an additional 13,000 knee replacements and 4,500 hip replacements each year. More evidence is therefore urgently needed on the other factors currently influencing both the decision to refer from the hub to surgical assessment and the decision on whether arthroplasty is the appropriate treatment option.

To our knowledge our study is the first to report the characteristics of patients attending a musculoskeletal hub. However, the analysis was based on a small sample and included only eight hub attendees with OKS/OHS ≥40. Data were analysed one month after the end of the period studied; some patients may therefore have gone on to have arthroplasty after physiotherapy or weight loss treatment. Furthermore, the retrospective review of medical records may not have identified all patients meeting exclusion criteria – particularly for patients who did not attend either the hub or surgical assessment. Furthermore, no data were collected on BMI.

Estimates of the impact of different policies rely on additional assumptions and represent an approximate indication of potential patient numbers; prospective pilot studies would be required to assess the true impact in practice. In particular, we focused on the impact of changing referral guidelines at hubs, as no data were available on patients visiting GPs with osteoarthritis symptoms. In practice, any decision aid or referral guideline is also likely to

For peer review only - http://bmjopen.bmj.com/site/about/guidelines.xhtml

affect GPs' referral decisions and the hub triage process, which could increase the number of additional operations resulting from any policy change. We assumed that decisions made by the patient and surgeon about whether to proceed to surgery after surgical assessment would be unaffected by the use of thresholds by the hub as OKS/OHS would have already been taken into account at the hub; the budget impact for patients referred via the hub could be lower than shown in Tables 1 and 2 if patients referred with high OKS/OHS were less likely to undergo surgery. We also assumed, arbitrarily, that 50% of people with OKS/OHS below the threshold who are not currently referred would choose not to be referred for surgery even if this were offered. The model also assumed that patients who do not undergo arthroplasty following their hub attendance would not have surgery for 10 years, since there are currently no data on how OKS/OHS change over time without arthroplasty.[5] In practice, many patients who would be eligible for surgery if the OKS/OHS threshold was raised would otherwise have had surgery later, after their condition had deteriorated. If the OKS/OHS thresholds were raised, the number of operations and budget impact might decrease over time as these patients would have been treated earlier, before their disease progresses, and would not need primary arthroplasty in the future.

Although access to joint replacement may be restricted if funding for interventions is reduced, results suggest that increased numbers of knee/hip replacements could be justified on cost-effectiveness grounds. Our results suggest that the NHS is currently willing to pay no more £2,000 per QALY gained from arthroplasty, while NICE routinely approves treatments for patients with other conditions (e.g. rheumatoid arthritis) that cost £20,000[21] or even £40,000[22]/QALY. This suggests that it may be more efficient and more equitable to spend limited NHS funds on conducting more joint replacements, increasing the operation rate in the UK to a level similar to that of Austria or Germany,[23] rather than directing these resources to other conditions. However, the number of procedures is also limited by availability of surgeons, operating theatres and hospital beds, which may mean that it is not currently feasible to conduct all of the operations that could be justified on cost-effectiveness grounds. And, as noted earlier, OKS/OHS is clearly not the only factor taken into account in decision-making by hubs and surgeons. In particular, patient choice plays a substantial role and comorbidities and other factors are also taken into account.

Referral hubs are an effective mechanism for referring patients to the most appropriate healthcare interventions/setting and patients are generally satisfied with this model of care.[8] One aim of introducing hubs was to improve efficiency in the referral pathway by increasing the proportion of referrals to secondary care that lead to surgery. The hub identifies patients who do not want surgery or who have mild symptoms and directs patients to other effective interventions when surgery is not appropriate. If the service works well, only patients who are suitable for surgery are referred. However, the process and criteria for arthroplasty referrals still vary greatly between CCGs.[1] Explicit guidelines

For peer review only - http://bmjopen.bmj.com/site/about/guidelines.xhtml

and decision aids could help reduce geographical inequity and ensure that all patients likely to benefit have fair access to cost-effective treatments. Although our analysis is based on retrospective data from only one hub, it provides initial estimates of what potential patient numbers and costs might be if a more explicit threshold-based approach was adopted nationally. While arthroplasty rates and patient characteristics in Oxfordshire are similar to the national average,[24 25] referral guidelines and pathways vary substantially between CCGs, suggesting that a broader study covering multiple hubs is necessary to provide a more comprehensive picture of current clinical practice. Ideally this would also collect more detailed patient information (e.g. BMI or comorbidities).

In conclusion, our paper has estimated the effect of introducing evidence-based OKS/OHS thresholds in England. While our results can be used to estimate the impact of other policies, including those where thresholds vary by age or other patient characteristics, a pilot study would be required to evaluate the actual impact of a policy change on routine clinical practice.

For peer review only

Declarations

Competing interest statement

All authors have completed the ICMJE uniform disclosure form at www.icmje.org/coi_disclosure.pdf and declare: a grant from NIHR for the submitted work; HD reports personal fees from Halyard Health, outside the submitted work; DB reports grants from Zimmer Biomet, outside the submitted work; AP reports personal fees from Zimmer Biomet and DePuy outside the submitted work; all declare that there are no other relationships or activities that could appear to have influenced the submitted work. The other authors declare that there are no relevant conflicts of interests besides the NIHR grant that supports the current work.

Details of contributors

AP is the guarantor for this study. AP, DB, HD, AG and KB conceived, designed and conducted the ACHE study. HD, AP and JS conceived and designed the study, which was conducted by HD and JS with assistance from Reza Mafi, and Sarah Dorman. HD, AG and PE conceived and designed the economic evaluation. HD conducted the statistical and economic analysis under the supervision of AG. AP and KB helped interpret the results. HD drafted the manuscript. All authors edited the manuscript for important intellectual content and approved the final version.

The following members of the ACHE study group all contributed to the developing the research question, planning the protocol of work, gaining funding and/or delivery of this research project: Ray Fitzpatrick, David Murray, Nigel Arden, Andy Carr, Karen Barker, James Smith, Stephanie Smith, Kristina Harris, Rob Middleton, Elizabeth Gibbons, Elena Benedetto, Jill Dawson, Adrian Sayers, Laura Miller, Elsa Marques, Rachael Gooberman-Hill, Ashley Blom, Andrew Judge, and Sion Glyn-Jones.

Copyright/licence

The Corresponding Author has the right to grant on behalf of all authors and does grant on behalf of all authors, an exclusive licence (or non-exclusive for government employees) on a worldwide basis to the BMJ Publishing Group Ltd and its Licensees to permit this article (if accepted) to be published in BMJ Open and any other BMJPGL products to exploit all subsidiary rights, as set out in our licence http://authors.bmj.com/submitting-your-paper/copyright-and-authors-rights/ and the Corresponding Author accepts and understands that any supply made under these terms is made by BMJPGL to the Corresponding Author. Where the Corresponding Author wishes to make the article available on an open access basis (and intends to pay the relevant open access fee), the terms of such open access shall be governed by a Creative Commons licence – details of these licences and which Creative Commons licence will apply to this article are set out in our licence referred to above.

For peer review only

Ethics statement

Trust management approval from the Oxford University Hospitals NHS Trust was obtained for retrospective analysis of anonymised data from medical records concerning outcomes in hip and knee arthroplasty (Oxford University Hospitals Research and Development Reference 11603). The plan to study referral patterns through the musculoskeletal hub, under the terms of the research and development agreement noted above, was discussed and agreed through the Clinical Governance Group for hip and knee replacement. The analysis was conducted to improve service understanding and used retrospective data on patients who had received normal clinical management. All data were anonymised by clinical staff.

Funding and role of study sponsors

The ACHE study and economic evaluation was funded by the NIHR Health Technology Assessment Programme (project number 11/63/01) and will be published in full in *Health Technology Assessment*. Further information available at: https://www.journalslibrary.nihr.ac.uk/programmes/hta/116301. This report presents independent research commissioned by the National Institute for Health Research (NIHR). The views and opinions expressed by authors in this publication are those of the authors and do not necessarily reflect those of the NHS, the NIHR, MRC, CCF, NETSCC, the Health Technology Assessment Programme, NHS, NHS Digital or the Department of Health. The funder had no involvement in: the collection, analysis, and interpretation of data; in the writing of the report; and in the decision to submit the article for publication. The authors had full access to the data (including statistical reports and tables), can take responsibility for the integrity of the data and the accuracy of the data analysis and are responsible for submitting this paper.

Transparency declaration

The manuscript's guarantor (AP) affirms that the manuscript is an honest, accurate, and transparent account of the study being reported; that no important aspects of the study have been omitted; and that any discrepancies from the study as planned (and, if relevant, registered) have been explained.

Data sharing

The decision tree and Markov models used in this paper are available from the corresponding author on request. PROMs/HES data are available from NHS Digital (http://content.digital.nhs.uk/dars).

Acknowledgements

We would like to thank the ACHE patient and public representatives, user group and steering group for their role in the project: Anne Clarkson Webb, Anthony Johnson, Chad

For peer review only - http://bmjopen.bmj.com/site/about/guidelines.xhtml

Lion Cachet, Fiona Watt, Fraser Old, Gill Dean, Gillian Kempster, Jannie Kramer, Jennifer Bostock, Jiyang Li, Jo Hewanicka, John Nolan, Kate Jackson, Laura Ingle, Mary Snow, Matthew Cheetham, Patricia Mary Rubery, Sharon Barrington, Sujin Kang, Tim Wilton, Vida Field, Sunny Deo, Malvin Drakely, Jon Campion, George Peat, Peter Kay, Jon Waite and Sue Woollacott. We would like to extend thanks to Reza Mafi, and Sarah Dorman who helped with data cleaning. We would also like to thank the reviewers for their insightful comments on the manuscript. Data on NHS PROMs linked to HES APC data were reused with the permission of NHS Digital, copyright 2015, with all rights reserved.

Patient involvement

The ACHE research question and study design (prior to funding) was informed by patient interest groups. The current analysis was based on pre-existing data from medical records so no patients were directly recruited in the course of the work. Throughout the ACHE study, a User Group, including patients and other stakeholders, informed progress and development of the work. Results of the analysis were discussed with user group including clinicians and patient representatives, to inform the presentation of results. The results will not be directly disseminated to patients whose records were included in the dataset, but the publication will be available for the target population.

References

1. The Royal College of Surgeons of England. Is access to surgery a postcode lottery? Secondary Is access to surgery a postcode lottery? 2014. https://www.rcseng.ac.uk/news-and-events/media-centre/press-releases/many-ccgs-are-ignoring-clinical-evidence-in-their-surgical-commissioning-policies/.

2. Harrogate and Rural District Clinical Commissioning Group. Clinical thresholds: Hip and knee arthroplasty for osteoarthritis (only). Secondary Clinical thresholds: Hip and knee arthroplasty for osteoarthritis (only). 2014. http://www.harrogateandruraldistrictccg.nhs.uk/data/uploads/rss2/hip-and-knee-arthroplasty.pdf.

3. Scarborough and Ryedale Clinical Commissioning Group. Hip replacement pathway. Secondary Hip replacement pathway 2015. http://www.scarboroughryedaleccg.nhs.uk/data/uploads/rss2/orthopaedics/hip-replacement-march-2015.pdf.

4. Price A, Kang S, Cook J, et al. The use of patient-reported outcome measures to guide referral for hip and knee replacement: Part 1 – the development of an evidence based model linking pre-operative score to the probability of gaining benefit from surgerythresholds. Submitted to Bone and Joint Journal 2019

5. Dakin H, Eibich P, Beard D, Gray A, Price A, on behalf of the ACHE Study team. The use of patient-reported outcome measures to guide referral for hip and knee replacement: Part 2 – a cost-effectiveness analysis. Submitted to Bone and Joint Journal 2019

For peer review only - http://bmjopen.bmj.com/site/about/guidelines.xhtml

6. Podmore B, Hutchings A, Durand MA, et al. Comorbidities and the referral pathway to access joint replacement surgery: an exploratory qualitative study. BMC health services research 2018;**18**(1):754 doi: 10.1186/s12913-018-3565-0[published Online First: Epub Date]|.

7. Button K, Morgan F, Hodgson H, Weightman AL, Jones S. Musculoskeletal care pathways for adults with hip and knee pain at the interface between primary and secondary care: protocol for a systematic review. Syst Rev 2016;**5**(1):128 doi: 10.1186/s13643-016-0301-x[published Online First: Epub Date]|.

8. Candy E, Haworth-Booth S, Knight-Davis M. Review of the Effectiveness of a Consultant Physiotherapy-Led Musculoskeletal Interface Team: A Welsh Experience. Musculoskeletal Care 2016;**14**(3):185-91 doi: 10.1002/msc.1122[published Online First: Epub Date]|.

9. Health and Social Care Information Centre. Finalised Patient Reported Outcome Measures (PROMs) in England, April 2014-March 2015 (annual report). Secondary Finalised Patient Reported Outcome Measures (PROMs) in England, April 2014-March 2015 (annual report) 2016. http://content.digital.nhs.uk/catalogue/PUB21189/final-proms-eng-apr14-mar15-fin-report.pdf.

10. Health and Social Care Information Centre. Finalised Patient Reported Outcome Measures (PROMs) in England, April 2013 to March 2014. Secondary Finalised Patient Reported Outcome Measures (PROMs) in England, April 2013 to March 2014 2015. http://www.hscic.gov.uk/catalogue/PUB17876/final-proms-eng-apr13-mar14-fin-report-v1.pdf.

11. Health and Social Care Information Centre. Finalised Patient Reported Outcome Measures (PROMs) in England, April 2012 to March 2013 (annual report). Secondary Finalised Patient Reported Outcome Measures (PROMs) in England, April 2012 to March 2013 (annual report) 2014. http://content.digital.nhs.uk/catalogue/PUB14574/final-proms-eng-apr12-mar13-fin-report-v1.pdf.

12. Health and Social Care Information Centre. Finalised Patient Reported Outcome Measures (PROMs) in England, April 2011 to March 2012 (annual report). Secondary Finalised Patient Reported Outcome Measures (PROMs) in England, April 2011 to March 2012 (annual report) 2013. http://content.digital.nhs.uk/catalogue/PUB11359/final-proms-eng-apr11-mar12-fin-report-v2.pdf.

13. Health and Social Care Information Centre. Finalised Patient Reported Outcome Measures (PROMs) in England, April 2010 to March 2011 (annual report). Secondary Finalised Patient Reported Outcome Measures (PROMs) in England, April 2010 to March 2011 (annual report) 2012. http://content.digital.nhs.uk/catalogue/PUB07049/fina-prom-eng-apr-10-mar-11-pre-post-rep1.pdf.

For peer review only

14. Health and Social Care Information Centre. Finalised Patient Reported Outcome Measures (PROMs) in England, April 2009 to March 2010 (annual report). Secondary Finalised Patient Reported Outcome Measures (PROMs) in England, April 2009 to March 2010 (annual report) 2011. http://content.digital.nhs.uk/catalogue/PUB02429/proms-eng-apr-09-mar-10-oper-data-expe-meth.pdf.
15. Moons KG, Royston P, Vergouwe Y, Grobbee DE, Altman DG. Prognosis and prognostic research: what, why, and how? BMJ 2009;**338**:b375 doi: 10.1136/bmj.b375[published Online First: Epub Date]|.
16. Coyle D, Buxton MJ, O'Brien BJ. Stratified cost-effectiveness analysis: a framework for establishing efficient limited use criteria. Health Econ 2003;**12**(5):421-7 doi: 10.1002/hec.788[published Online First: Epub Date]|.
17. Espinoza MA, Manca A, Claxton K, Sculpher MJ. The value of heterogeneity for cost-effectiveness subgroup analysis: conceptual framework and application. Med Decis Making 2014;**34**(8):951-64 doi: 10.1177/0272989X14538705[published Online First: Epub Date]|.
18. National Institute for Health and Care Excellence. Osteoarthritis: NICE quality standard 87. Secondary Osteoarthritis: NICE quality standard 87 June 2015. https://www.nice.org.uk/guidance/qs87.
19. National Institute for Health and Care Excellence. Osteoarthritis: the care and management of osteoarthritis in adults: NICE clinical guideline 177. Secondary Osteoarthritis: the care and management of osteoarthritis in adults: NICE clinical guideline 177 February 2014. https://www.nice.org.uk/guidance/cg177.
20. Royal College of Surgeons. Commissioning guide: Painful osteoarthritis of the knee, Version 1.1. Secondary Commissioning guide: Painful osteoarthritis of the knee, Version 1.1 July 2014. https://www.boa.ac.uk/wp-content/uploads/2015/01/Enc-8d_Painful-osteoarthritis-of-the-knee_Revised-final.pdf.
21. National Institute for Health and Clinical Excellence. Social value judgements: Principles for the development of NICE guidance. Second Edition. Secondary Social value judgements: Principles for the development of NICE guidance. Second Edition 2008. https://www.nice.org.uk/Media/Default/About/what-we-do/Research-and-development/Social-Value-Judgements-principles-for-the-development-of-NICE-guidance.pdf.
22. Dakin H, Devlin N, Feng Y, Rice N, O'Neill P, Parkin D. The Influence of Cost-Effectiveness and Other Factors on Nice Decisions. Health Econ 2015;**24**(10):1256-71 doi: 10.1002/hec.3086[published Online First: Epub Date]|.
23. OECD. Hip and knee replacement. Health at a Glance 2015: OECD, 2015:112–13.
24. Right Care. The NHS Atlas of variation in health care: reducing unwarranted variation to increase value and improve quality. Secondary The NHS Atlas of variation in health care: reducing unwarranted variation to increase value and improve quality 2010. http://fingertips.phe.org.uk/documents/Atlas_2011_MSK.pdf.

For peer review only - http://bmjopen.bmj.com/site/about/guidelines.xhtml

25. Health and Social Care Information Centre. Finalised Patient Reported Outcome Measures in England - April 2014 to March 2015: Score Comparison Tool. Secondary Finalised Patient Reported Outcome Measures in England - April 2014 to March 2015: Score Comparison Tool 2016. http://content.digital.nhs.uk/catalogue/PUB21189.
26. Hospital Episode Statistics. Admitted patient care England 2014-15: Procedures and interventions. Secondary Admitted patient care England 2014-15: Procedures and interventions 2015. http://www.hscic.gov.uk/catalogue/PUB19124/hosp-epis-stat-admi-proc-2014-15-tab.xlsx.

For peer review only

For peer review only - http://bmjopen.bmj.com/site/about/guidelines.xhtml

Table 1 Impact of different OKS thresholds on patient numbers, costs and QALYs among the 172,192 patients with knee osteoarthritis attending the hub in England each year. The results presented exclude patients who did not attend face-to-face consultations at the hub; based on our analysis, 31% (24,063/76,617) of knee replacements are conducted on patients who attended the hub.

	Current practice	Maximum OKS at which patients can be referred for surgical assessment					
		18	24	31	35	40	43
Number of attendances at the surgical outpatient visit	80,211	65,286	99,982	123,635	129,256	131,633	131,983
Number of arthroplasty procedures conducted (% change*)	24,063	19,586 (-6%)	29,995 (+8%)	37,090 (+17%)	38,777 (+19%)	39,490 (+20%)	39,595 (+20%)
Total cost over 10 years (thousands)	£1,098,213	£1,081,313	£1,108,836	£1,134,134	£1,142,073	£1,146,019	£1,146,678
Total QALYs over 10 years	564,744	566,658	585,674	594,802	596,910	597,778	597,856
Net health benefit (QALYs)	509,834	512,593	530,232	538,096	539,806	540,477	540,522

* Percentage change in the total number of arthroplasty procedures following a change to referral patterns at the hub. Equal to the difference in the number of procedures between the scenario in question and 'current practice', divided by the 76,617 knee replacements conducted in England each year.[26]

Table 2 Impact of different OHS thresholds on patient numbers, costs and QALYs among the 41,121 patients with hip osteoarthritis attending the hub in England each year. The results presented exclude patients who did not attend face-to-face consultations at the hub; based on our analysis, 9% (6,590/69,313) of hip replacements are conducted on patients who attended the hub.

	Current practice	Maximum OHS at which patients can be referred for surgical assessment					
		18	24	30	35	40	45
Number of attendances at the surgical outpatient visit	17,793	18,036	25,420	29,871	31,561	32,108	32,216
Number of arthroplasty procedures conducted (% change*)	6,590	6,680 (+0%)	9,415 (+4%)	11,063 (+6%)	11,689 (+7%)	11,892 (+8%)	11,932 (+8%)
Total cost over 10 years (thousands)	£168,402	£168,202	£182,080	£190,519	£193,753	£194,816	£195,030
Total QALYs over 10 years	123,674	128,990	135,863	138,888	139,929	140,248	140,288
Net health benefit (QALYs)	115,254	120,580	126,759	129,362	130,242	130,508	130,537

* Percentage change in the total number of arthroplasty procedures following a change to referral patterns at the hub. Equal to the difference in the number of procedures between the scenario in question and 'current practice', divided by the 69,313 hip replacements conducted in England each year.[26]

For peer review only - http://bmjopen.bmj.com/site/about/guidelines.xhtml

Figure 1 Patient flow diagram for patients referred with knee symptoms

* See Table A2 in online supplementary appendix for a list of the conditions other than osteoarthritis for which patients aged ≥50 years were excluded from the analysis.

For peer review only

Figure 2 Patient flow diagram for patients referred with hip symptoms

* See Table A2 in online supplementary appendix for a list of the conditions other than osteoarthritis for which patients aged ≥50 years were excluded from the analysis.

For peer review only

For peer review only - http://bmjopen.bmj.com/site/about/guidelines.xhtml

Figure 3 Number of patients predicted to be referred with (A) knee and (B) hip osteoarthritis symptoms in England

For peer review only

For peer review only - http://bmjopen.bmj.com/site/about/guidelines.xhtml

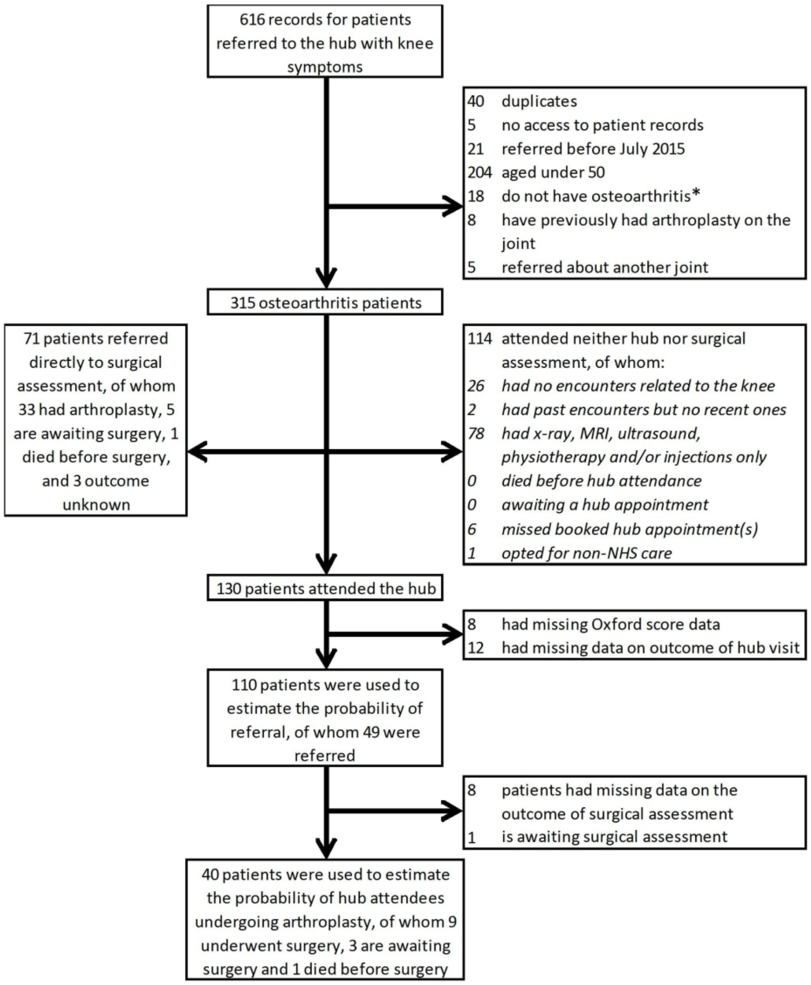

Figure 1 Patient flow diagram for patients referred with knee symptoms

* See Table A2 in online supplementary appendix for a list of the conditions other than osteoarthritis for which patients aged ≥50 years were excluded from the analysis.

190x274mm (284 x 284 DPI)

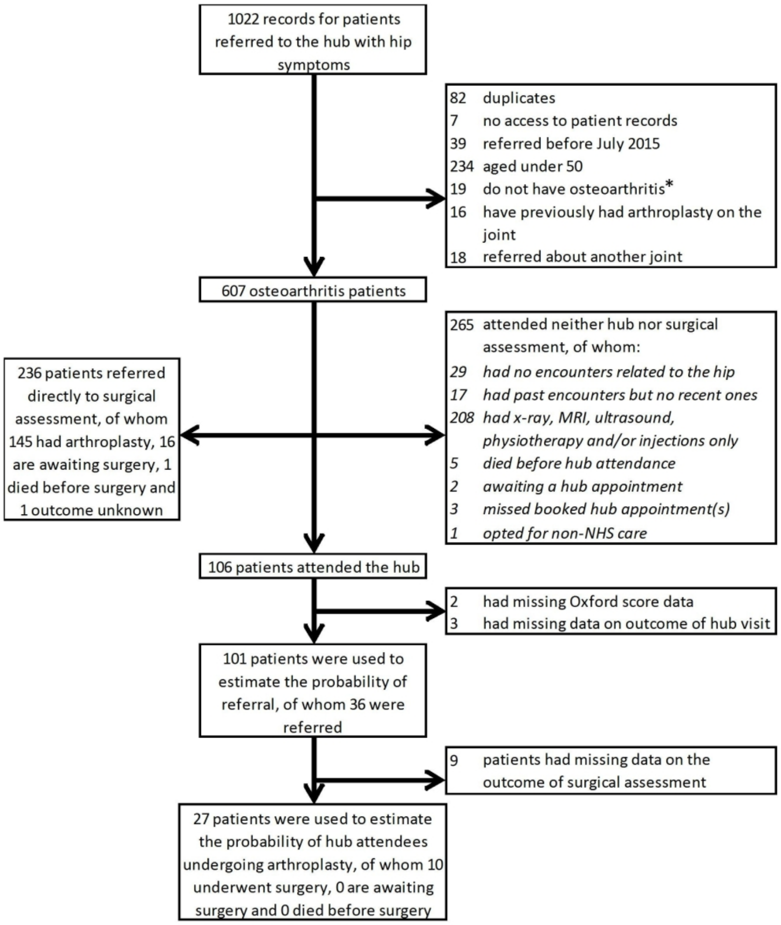

Figure 2 Patient flow diagram for patients referred with hip symptoms

* See Table A2 in online supplementary appendix for a list of the conditions other than osteoarthritis for which patients aged ≥50 years were excluded from the analysis.

190x274mm (284 x 284 DPI)

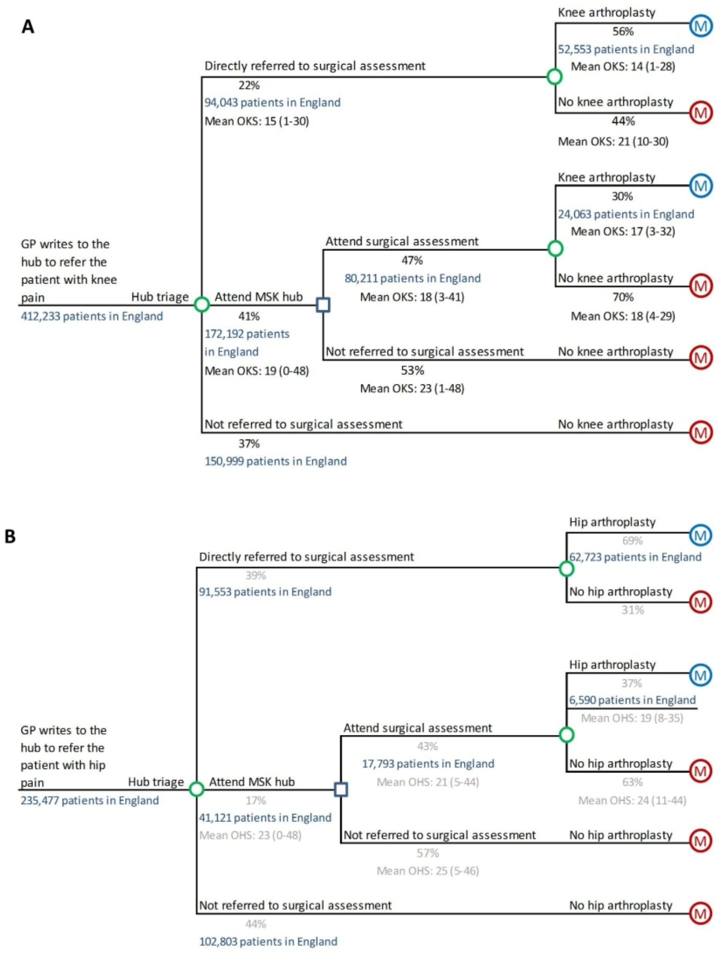

Figure 3 Number of patients predicted to be referred with (A) knee and (B) hip osteoarthritis symptoms in England

190x274mm (284 x 284 DPI)

Online supplementary file

Methods for stepwise regression models

We selected variables for inclusion in the logistic regression model in a pre-specified sequence. We first estimated a model only including the constant term and evaluated whether adding OKS/OHS to the model reduced Akaike information criterion (AIC); if AIC was reduced, the model used in the next step controlled for OKS/OHS and otherwise remained with just a constant term. Similarly, the next step assessed whether adding age into the model reduced AIC and in the final step we assessed whether adding sex into the model reduced AIC. We specified the order in which variables were considered for the model (OKS/OHS, then age, then sex) in advance of data analysis: it was hypothesised that OKS/OHS would be the most important factor determining referrals, followed by age (since this is a proxy for comorbidities) and finally sex (since we had no prior hypotheses about how sex would affect referrals). Age and OKS/OHS were assumed to have linear effects on the log-odds of being referred for surgical assessment as there were insufficient data to evaluate nonlinear functions.

Methods for estimating patient numbers

Hub data were extrapolated to estimate patient numbers across England by applying the probability of referral estimated on the hub data to national data on the characteristics of patients undergoing arthroplasty and the total number of operations.

We considered 490 groups of patients, defined by gender, age (using age bands <55, 55-64, 65-74, 75-84 and ≥85) and individual preoperative OKS/OHS between 0 and 48. An extract of individual patient data from the national patient reported outcome measures (PROMs) dataset linked to Hospital Episode Statistics (HES) covering operations conducted between 2009 and 2015 was used to estimate the proportion of arthroplasty patients in different groups defined by age and gender. The same dataset was used to obtain the proportion of patients with each preoperative OKS/OHS.

The number of operations in each of the 490 patients groups was calculated by multiplying the proportion of patients in different age and sex groups by the proportion with different OKS/OHS and by the number of primary knee/hip replacements conducted solely for osteoarthritis in England in 2014-15. The total number of primary knee/hip replacements conducted for osteoarthritis (76,617 knee replacements and 69,313 hip replacements) was calculated by multiplying the total number of finished consultant episodes in HES[1] with the OPCS codes beginning with O or W that the National joint Registry (NJ) used to identify primary hip/knee replacement[2] (79,726 knee replacements and 77,880 hip replacements) by the proportion of primary arthroplasty procedures that are conducted solely for osteoarthritis (96% for knees and 89% for hips).[3]

For peer review only - http://bmjopen.bmj.com/site/about/guidelines.xhtml

We then calculated the number of patients in each group who are likely to have been referred by GPs with knee/hip symptoms by dividing the number of operations in each group by the probability that each group of patients will undergo surgery.

Unit costs

The unit costs used in the current analysis are shown in Table A1. The costs and QALYs accrued in the 10-year period after arthroplasty (or a decision not to perform arthroplasty) have been reported previously.[4]

Table A1 Unit costs

Healthcare resource	Cost per consultation	Reference and details
Attendance at musculoskeletal hub	£58	Based on 40 minutes' of patient-related activity for a grade 8a physiotherapist. Based on activity at the NOC hub, each hub attendance lasts 30 minutes, plus an additional 10 minutes' administration per patient and will, on average, be conducted by a grade 8a physiotherapist who spends around 80% of their time on patient-related activity. The salary for a band 8a, point 36 physiotherapist was based on Agenda for Change salaries[5] and converted to an hourly rate by applying the same methodology as was applied by PSSRU for a hospital physiotherapist,[6] assuming that working hours and the percentages for salary on costs, qualifications and overheads would be the same for all hospital physiotherapists.
Outpatient consultation with an orthopaedic surgeon	£132	National average unit cost for first consultant-led non-admitted face-to-face attendance, WF01B, CL sheet of the National Schedule of Reference Costs Year 2013-14 NHS trusts and NHS foundation trusts[7]

For peer review only

Additional results

Table A2 Conditions other than osteoarthritis for which patients aged ≥50 years were excluded from the analysis

	Number of patients	
	**Knee audit**	**Hip audit**
Total patients aged ≥50 excluded due to conditions other than osteoarthritis	18	19
Rheumatoid arthritis	5	9
Bone or joint infections	4	2
Requiring limb reconstruction	1	2
Injury	2	1
No mechanical symptoms	1	1
Fracture	0	3
Arthritis secondary to fracture	2	0
Symptoms caused by previous spinal surgery	0	1
Gout	1	0
Chondroid lesion	1	0
Quadriceps rupture	1	0

Table A3 Characteristics of the patients included in the hub audit

	**Number of patients**	**Mean age (range)**	**% Female (n/N)**	**Mean OKS/OHS (range; N)**
Knee patients				
All osteoarthritis patients	315	66 (50, 93)	52% (165/315)	-
All patients referred directly to surgical assessment	71	68 (51, 86)	61% (43/71)	15 (1, 30; N=27)
Patients not referred for arthroplasty following direct referral to surgical assessment	*30*	*63 (51, 86)*	*53% (16/30)*	*21 (10, 30; N=5)*
Patients referred for arthroplasty following direct referral to surgical assessment	*38*	*72 (55, 86)*	*63% (24/38)*	*14 (1, 28; N=22)*
Patients attending neither hub nor surgical assessment	114	66 (50, 86)	49% (56/114)	-
All patients attending the hub	130	65 (50, 93)	51% (66/130)	19 (0, 48; N=130)
Patients not referred to surgical assessment following hub attendance	*61*	*64 (50, 88)*	*44% (27/61)*	*23 (1, 48; N=61)*
Patients referred to surgical assessment following hub attendance	*49*	*65 (51, 88)*	*49% (24/49)*	*18 (3, 41; N=49)*

For peer review only - http://bmjopen.bmj.com/site/about/guidelines.xhtml

	Number of patients	Mean age (range)	% Female (n/N)	Mean OKS/OHS (range; N)
Patients referred for arthroplasty following hub attendance and surgical assessment	*12*	*66 (55, 88)*	*33% (4/12)*	*17 (3, 32; N=12)*
Patients not referred for arthroplasty following hub attendance and surgical assessment	*28*	*65 (51, 82)*	*57% (16/28)*	*18 (4, 29; N=28)*
Hip patients				
All osteoarthritis patients	607	70 (50, 95)	67% (407/607)	-
All patients referred directly to surgical assessment	236	72 (50, 95)	67% (158/236)	-
Patients not referred for arthroplasty following direct referral to surgical assessment	*74*	*71 (51, 95)*	*80% (59/74)*	-
Patients referred for arthroplasty following direct referral to surgical assessment	*161*	*72 (50, 92)*	*61% (98/161)*	-
Patients attending neither hub nor surgical assessment	265	70 (50, 93)	64% (170/265)	-
All patients attending the hub	106	67 (50, 89)	75% (79/106)	23 (0, 48; N=106)
Patients not referred to surgical assessment following hub attendance	*65*	*67 (51, 89)*	*80% (52/65)*	*25 (5, 46; N=65)*
Patients referred to surgical assessment following hub attendance	*36*	*67 (50, 85)*	*67% (24/36)*	*21 (5, 44; N=36)*
Patients referred for arthroplasty following hub attendance and surgical assessment	*10*	*72 (56, 85)*	*50% (5/10)*	*19 (8, 35; N=10)*
Patients not referred for arthroplasty following hub attendance and surgical assessment	*17*	*67 (51, 84)*	*65% (11/17)*	*24 (11, 44; N=17)*

For peer review only

FIGURE A1 Distribution of (**A**) OKS and (**B**) OHS for patients attending the hub with knee/hip pain compared with the distribution of patients undergoing knee arthroplasty in England in PROMs/HES (2009-2015)

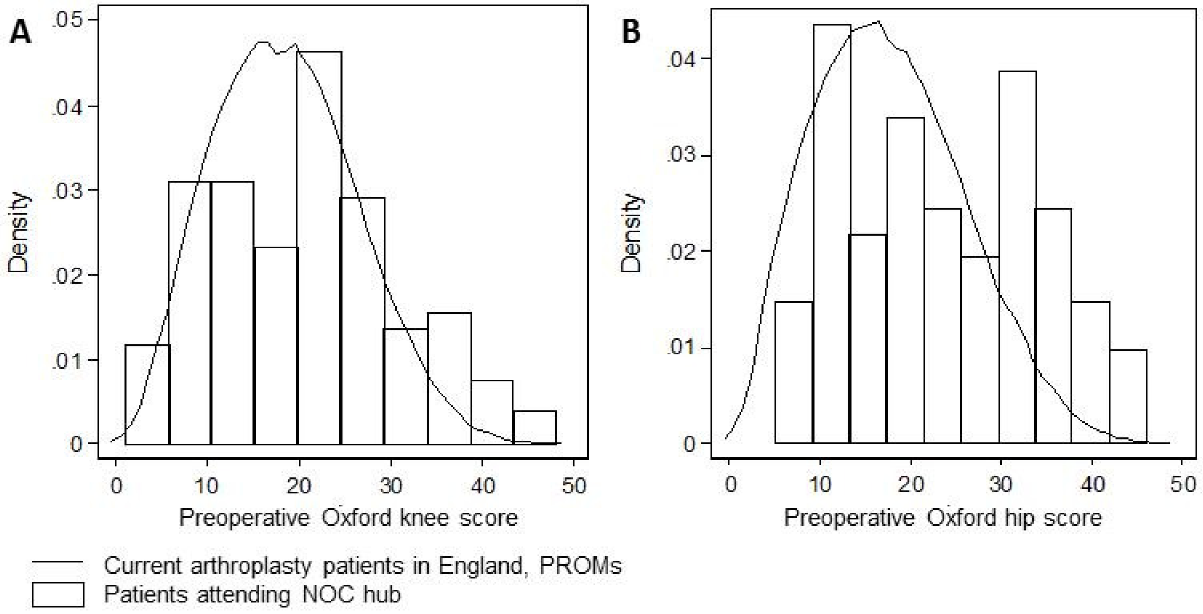

Additional regression results

For patients attending the hub with knee symptoms, adding age and sex into the model predicting the odds of hub attendees being referred for surgical assessment increased AIC, so the final model included only OKS (Table A4). Similarly, in the model of hip patients, adding age into the model worsened AIC, so the final model included only OHS and gender. Omitting gender from the model had negligible effect on the coefficient for OHS, which was 0.0397 (SE: 0.0211) when OHS was the only explanatory variable.

TABLE A4 Results of logistic regression predicting the odds of patients attending the hub being referred for surgical assessment

Variable	Mean coefficient (SE)	
	Knee patients (N=110)	Hip patients (N=101)
OKS/OHS	-0.048 (0.021)*	-0.0397 (0.0213)
Male	-	0.6965 (0.4804)
Constant	0.768 (0.459)	0.1428 (0.5376)
Pseudo-R^2	0.0389	0.0441

* p<0.05

In addition to the logistic regression model predicting the odds of patients being referred from the hub to surgical assessment, we also conducted a secondary analysis predicting the odds of undergoing arthroplasty in patients who had already been referred to surgical assessment by the hub, using the same methods. This analysis suggested that OKS/OHS, age and sex had no significant effect on the odds of referral from surgical assessment to surgery

For peer review only - http://bmjopen.bmj.com/site/about/guidelines.xhtml

(p>0.05) and that including any of these variables in the regression increased AIC compared with a model with only a constant term. This validates the assumption that the probability of patients referred directly to surgical assessment subsequently being referred for surgery is independent of OKS/OHS, age and sex and the assumption that introducing OKS/OHS thresholds at the hub would have no effect on the probability of a patient with particular preoperative characteristics being referred for arthroplasty following a surgical assessment visit. However, this analysis is likely to be underpowered, since only 12 knee patients and 10 hip patients underwent surgery after face-to-face consultations at the hub and surgical assessment.

References

1. Hospital Episode Statistics. Admitted patient care England 2014-15: Procedures and interventions. Secondary Admitted patient care England 2014-15: Procedures and interventions 2015. http://www.hscic.gov.uk/catalogue/PUB19124/hosp-epis-stat-admi-proc-2014-15-tab.xlsx.
2. National Joint Registry for England and Wales. OPCS codes relevant procedure is recorded in the NJR. Secondary OPCS codes relevant procedure is recorded in the NJR 2013. http://www.njrcentre.org.uk/njrcentre/Portals/0/Documents/England/Data%20collection%20forms/OPCS%20Procedure%20codes%20relevant%20to%20NJR.pdf.
3. National Joint Registry for England and Wales. 13th Annual Report. Secondary 13th Annual Report 2016. www.njrcentre.org.uk.
4. Dakin H, Eibich P, Beard D, Gray A, Price A, on behalf of the ACHE Study team. The use of patient-reported outcome measures to guide referral for hip and knee replacement: Part 2 – a cost-effectiveness analysis. Submitted to Bone and Joint Journal 2019
5. NHS Employers. Agenda for change pay bands and points from 1 April 2014. Secondary Agenda for change pay bands and points from 1 April 2014 2014. http://www.nhsemployers.org/~/media/Employers/Documents/Pay%20and%20reward/AfC%20pay%20bands%20and%20points%20poster%202014.pdf.
6. Curtis L. Unit Costs of Health and Social Care 2014. Secondary Unit Costs of Health and Social Care 2014 2014. http://www.pssru.ac.uk/project-pages/unit-costs/2014/.
7. Department of Health. NHS reference costs 2013 to 2014 - National schedule of reference costs: the main schedule. Secondary NHS reference costs 2013 to 2014 - National schedule of reference costs: the main schedule 2014. https://www.gov.uk/government/publications/nhs-reference-costs-2013-to-2014.

For peer review only - http://bmjopen.bmj.com/site/about/guidelines.xhtml

STROBE 2007 (v4) Statement—Checklist of items that should be included in reports of *cross-sectional studies*

Section/Topic	Item #	Recommendation	Section and page number where reported
**Title and abstract**	1	(*a*) Indicate the study's design with a commonly used term in the title or the abstract	Title pg 1
		(*b*) Provide in the abstract an informative and balanced summary of what was done and what was found	Title pg 1
**Introduction**			
Background/rationale	2	Explain the scientific background and rationale for the investigation being reported	Introduction pg 5
Objectives	3	State specific objectives, including any prespecified hypotheses	Introduction pg 5
**Methods**			
Study design	4	Present key elements of study design early in the paper	Strengths and limitations pg 5
Setting	5	Describe the setting, locations, and relevant dates, including periods of recruitment, exposure, follow-up, and data collection	Methods page 6
Participants	6	(*a*) Give the eligibility criteria, and the sources and methods of selection of participants	Methods page 6
Variables	7	Clearly define all outcomes, exposures, predictors, potential confounders, and effect modifiers. Give diagnostic criteria, if applicable	Methods page 6
Data sources/ measurement	8*	For each variable of interest, give sources of data and details of methods of assessment (measurement). Describe comparability of assessment methods if there is more than one group	Methods page 6
Bias	9	Describe any efforts to address potential sources of bias	*N/A*
Study size	10	Explain how the study size was arrived at	Methods page 6
Quantitative variables	11	Explain how quantitative variables were handled in the analyses. If applicable, describe which groupings were chosen and why	Methods page 6-7
Statistical methods	12	(*a*) Describe all statistical methods, including those used to control for confounding	Methods pages 6-7 and page 1 of online supplementary appendix
		(*b*) Describe any methods used to examine subgroups and interactions	N/A
		(*c*) Explain how missing data were addressed	Methods page7

For peer review only

		(*d*) If applicable, describe analytical methods taking account of sampling strategy	N/A
		(*e*) Describe any sensitivity analyses	N/A
**Results**			
Participants	13*	(a) Report numbers of individuals at each stage of study—eg numbers potentially eligible, examined for eligibility, confirmed eligible, included in the study, completing follow-up, and analysed	Figures 1-2 pages 22-23 and 25-26
		(b) Give reasons for non-participation at each stage	Figures 1-2 (pages 22-23 and 25-26) and Table A2 (page 3 of online supplementary appendix)
		(c) Consider use of a flow diagram	Figures 1-2 (pages 22-23 and 25-26)
Descriptive data	14*	(a) Give characteristics of study participants (eg demographic, clinical, social) and information on exposures and potential confounders	Table A3, Figure A1 (pages 3-5 of online supplementary appendix)
		(b) Indicate number of participants with missing data for each variable of interest	Figures 1-2 (pages 22-23 and 25-26) and Table A3 (pages 3-4 of online supplementary appendix)
Outcome data	15*	Report numbers of outcome events or summary measures	Figures 1-3 (pages 22-27)
Main results	16	(*a*) Give unadjusted estimates and, if applicable, confounder-adjusted estimates and their precision (eg, 95% confidence interval). Make clear which confounders were adjusted for and why they were included	Online supplementary appendix pages 5-6
		(*b*) Report category boundaries when continuous variables were categorized	N/A
		(*c*) If relevant, consider translating estimates of relative risk into absolute risk for a meaningful time period	N/A
Other analyses	17	Report other analyses done—eg analyses of subgroups and interactions, and sensitivity analyses	Methods pages 6-8 and online supplementary file pages 5-6
**Discussion**			
Key results	18	Summarise key results with reference to study objectives	Discussion, paragraph 1 (page 12)
Limitations	19	Discuss limitations of the study, taking into account sources of potential bias or imprecision. Discuss both direction and magnitude of any potential bias	Discussion, paragraphs 2-3 (pages 12-13

For peer review only

Interpretation	20	Give a cautious overall interpretation of results considering objectives, limitations, multiplicity of analyses, results from similar studies, and other relevant evidence	Discussion (pages 12-14
Generalisability	21	Discuss the generalisability (external validity) of the study results	Discussion, paragraph 5 (page 14)
**Other information**			
Funding	22	Give the source of funding and the role of the funders for the present study and, if applicable, for the original study on which the present article is based	Funding and role of study sponsors (page 16)

*Give information separately for cases and controls in case-control studies and, if applicable, for exposed and unexposed groups in cohort and cross-sectional studies.

Note: An Explanation and Elaboration article discusses each checklist item and gives methodological background and published examples of transparent reporting. The STROBE checklist is best used in conjunction with this article (freely available on the Web sites of PLoS Medicine at http://www.plosmedicine.org/, Annals of Internal Medicine at http://www.annals.org/, and Epidemiology at http://www.epidem.com/). Information on the STROBE Initiative is available at www.strobe-statement.org.

For peer review only

BMJ Open

Who gets referred for knee or hip replacement? A theoretical model of the potential impact of evidence-based referral thresholds using data from a retrospective review of clinic records from an English musculoskeletal referral hub

Journal:	*BMJ Open*
Manuscript ID	bmjopen-2019-028915.R2
Article Type:	Original research
Date Submitted by the Author:	16-Sep-2019
Complete List of Authors:	Dakin, Helen; Oxford University, Health Economics Research Centre, Nuffield Department of Population Health<br>Eibich, Peter; Max-Planck-Institut fur Demografische Forschung; Oxford University, Health Economics Research Centre, Nuffield Department of Population Health<br>Gray, Alastair; Oxford University, Health Economics Research Centre, Nuffield Department of Population Health<br>Smith, James; Oxford University, Nuffield Department of Orthopaedics, Rheumatology and Musculoskeletal Sciences; Macquarie University, Centre for Healthcare Resilience and Implementation Science, Australian Institute of Health Innovation<br>Barker, Karen L.; Oxford University, Nuffield Department of Orthopaedics, Rheumatology and Musculoskeletal Sciences<br>Beard, David; Oxford University, Nuffield Department of Orthopaedics, Rheumatology and Musculoskeletal Sciences<br>Price, Andrew; Oxford University, Nuffield Department of Orthopaedics, Rheumatology and Musculoskeletal Sciences
**Primary Subject Heading**:	Surgery
Secondary Subject Heading:	General practice / Family practice, Health economics, Health policy, Rheumatology
Keywords:	Arthroplasty, hip replacement, knee replacement, prioritisation, osteoarthritis

SCHOLARONE™
Manuscripts

Who gets referred for knee or hip replacement? A theoretical model of the potential impact of evidence-based referral thresholds using data from a retrospective review of clinic records from an English musculoskeletal referral hub

Helen Dakin,[1] Peter Eibich,[1,2] Alastair Gray,[1] James Smith,[3,4] Karen Barker,[3] David Beard,[3] Andrew Price[3] on behalf of the ACHE Study team

[1] Health Economics Research Centre, Nuffield Department of Population Health, University of Oxford, UK

[2] Max Planck Institute for Demographic Research, Rostock, Germany

[3] Nuffield Department of Orthopaedics, Rheumatology, and Musculoskeletal Science, NIHR Biomedical Research Unit, University of Oxford, Oxford, UK

[4] Centre for Healthcare Resilience and Implementation Science, Macquarie University, Australia

Contributors: Sujin Kang, Jonathan Cook, Ray Fitzpatrick, David Murray, Nigel Arden, Andy Carr, Stephanie Smith, Kristina Harris, Rob Middleton, Elizabeth Gibbons, Elena Benedetto, Jill Dawson, Adrian Sayers, Laura Miller, Elsa Marques, Rachael Gooberman-Hill, Ashley Blom, Andrew Judge, and Sion Glyn-Jones.

Short title for running header: Who gets referred for joint replacement?

Contact details for authors:

Helen Dakin [corresponding author]: Senior Researcher, Health Economics Research Centre, Nuffield Department of Population Health, University of Oxford, Old Road Campus, Headington, Oxford, OX3 7LF, UK. Email: helen.dakin@ndph.ox.ac.uk; Tel: 01865 289422. http://orcid.org/0000-0003-3255-748X

Peter Eibich: Senior Researcher, Health Economics Research Centre, Nuffield Department of Population Health, University of Oxford, Old Road Campus, Headington, Oxford, OX3 7LF, UK. Research Scientist, Max Planck Institute for Demographic Research, Konrad-Zuse-Str. 1, 18057 Rostock, Germany. Email: eibich@demogr.mpg.de. http://orcid.org/0000-0002-0689-0302

Alastair Gray: Professor of Health Economics, Health Economics Research Centre, Nuffield Department of Population Health, University of Oxford, Old Road Campus, Headington, Oxford, OX3 7LF, United Kingdom. Email: alastair.gray@ndph.ox.ac.uk. http://orcid.org/0000-0003-0239-7278

James Smith: Research Fellow, Nuffield Department of Orthopaedics, Rheumatology and Musculoskeletal Sciences, University of Oxford, Botnar Research Centre, Windmill Road,

For peer review only

Headington, Oxford, OX3 7LD, UK. Centre for Healthcare Resilience and Implementation Science, Australian Institute of Health Innovation, Macquarie University, NSW 2109, Australia. Email: jim.smith@mq.edu.au. https://orcid.org/0000-0002-0448-8774

Karen Barker: Clinical Director, Nuffield Orthopaedic Centre, Oxford University Hospitals Trust, Oxford, OX3 7HE, UK; Professor of Physiotherapy, Nuffield Department of Orthopaedics, Rheumatology and Musculoskeletal Sciences, University of Oxford, Botnar Research Centre, Windmill Road, Headington, Oxford, OX3 7LD, UK. Email: Karen.Barker@ouh.nhs.uk. http://orcid.org/0000-0001-9363-0383

David Beard: Professor of Musculoskeletal and Surgical Science, Nuffield Department of Orthopaedics, Rheumatology and Musculoskeletal Sciences, University of Oxford, Botnar Research Centre, Windmill Road, Headington, Oxford, OX3 7LD, UK. Email: david.beard@ndorms.ox.ac.uk. https://orcid.org/0000-0001-7884-6389

Andrew Price [guarantor]: Professor of Orthopaedic Surgery, Nuffield Department of Orthopaedics, Rheumatology and Musculoskeletal Sciences, University of Oxford, Botnar Research Centre, Windmill Road, Headington, Oxford, OX3 7LD, UK. Email: andrew.price@ndorms.ox.ac.uk. https://orcid.org/0000-0002-4258-5866

Institution where the work was carried out: Health Economics Research Centre and Nuffield Department of Orthopaedics, Rheumatology, and Musculoskeletal Science, University of Oxford

Word Count: 4453
Tables: 2
Figures: 3

For peer review only

Abstract

Objectives: To estimate the relationship between patient characteristics and referral decisions made by musculoskeletal hubs, and assess the possible impact of an evidence-based referral tool.

Design: Retrospective analysis of medical records and decision-tree model evaluating policy changes using local and national data.

Setting: One musculoskeletal interface clinic (hub) in England.

Participants: 922 adults aged ≥50 years referred by GPs with symptoms of knee or hip osteoarthritis.

Interventions: We assessed the current frequency and determinants of referrals from one hub and the change in referrals that would occur at this centre and nationally if evidence-based thresholds for referral (Oxford knee and hip scores, OKS/OHS) were introduced.

Main outcome measure: OKS/OHS, referrals for surgical assessment, referrals for arthroplasty, costs and quality adjusted life-years (QALYs).

Results: Of 110 patients with knee symptoms attending face-to-face hub consultations, 49 (45%) were referred for surgical assessment; mean OKS for these 49 patients was 18 (range: 1-41). Of 101 hip patients, 36 (36%) were referred for surgical assessment (mean OHS: 21, range: 5-44). No patients referred for surgical assessment were above previously-reported economic thresholds for OKS (43) or OHS (45). Setting thresholds of OKS≤31 and OHS≤35 might have resulted in an additional 22 knee referrals and 26 hip referrals in our cohort. Extrapolating hub results across England suggests a possible increase in referrals nationally, of around 13,000 additional knee replacements and 4,500 additional hip replacements each year.

Conclusions: Musculoskeletal hubs currently consider OKS/OHS and other factors when making decisions about referral to secondary care for joint replacement. Those referred typically have low OHS/OKS scores and introducing evidence-based OKS/OHS thresholds would prevent few inappropriate (high-functioning, low-pain) referrals. However, our findings suggest that some patients not currently referred could benefit from arthroplasty based on OKS/OHS. More research is required to explore other important patient characteristics currently influencing hub decisions.

Keywords

Arthroplasty; hip replacement; knee replacement; prioritisation; osteoarthritis.

For peer review only

Strengths and limitations of this study

- This is the first analysis reporting data on the characteristics of patients being referred to a UK hub with symptoms of hip or knee osteoarthritis; such data are needed to assess the impact of changing referral criteria on the number of knee/hip replacements and on costs and quality of life.
- We retrospectively reviewed clinical records for 922 men and women referred to a single musculoskeletal hub, although only 221 patients underwent arthroplasty.
- We used several assumptions to extrapolate our estimates of how the probability of referral varies with preoperative characteristics using a decision-tree and Markov model, which enabled us to estimate the potential impact of different referral policies locally and nationally.
- A prospective pilot study would be required to quantify the real-world impact of any policy change, while data from multiple hubs and information on other patient characteristics (such as body mass index) are required for a comprehensive understanding of current clinical practice.

For peer review only

Introduction

Many clinical commissioning groups (CCGs) in the UK have set referral criteria for joint replacement that exclude patients with Oxford knee and hip scores (OKS and OHS) above certain thresholds, which are often as low as 19[1] or 24[1-3] out of 48. Recent papers have shown that these low thresholds are inappropriate: arthroplasty has a ≥80% chance of producing a meaningful improvement in patients with OKS/OHS of 19 or 24[4] and would cost <£10,000 per quality adjusted life-year (QALY).[5] The recent ACHE study analysed >400,000 medical records and concluded that referral thresholds between 31 and 41 may be justified on clinical and cost-effectiveness grounds.[4 5]

Commissioners' decisions concerning thresholds may be influenced by the likely number of operations and their cost. In the UK, >94% of patients currently undergoing arthroplasty have OKS/OHS ≤31, demonstrating that nearly all operations currently conducted are cost-effective and have ≥70% chance of meaningful improvement.[4 5] However, there are very little data on how patient characteristics influence the assessment process in practice, or on patients who do not currently receive surgery, making it difficult to assess the impact that different referral criteria may have on patient numbers or costs. As in many other clinical areas, hubs (also known as triage clinics, musculoskeletal interface services or intermediate musculoskeletal assessment centres) are increasingly used as gatekeepers determining access to consultations with orthopaedic surgeons, assessing all patients being considered for surgery.[6-8]

We aimed to review the characteristics of patients attending hub consultations, estimate the proportion of patients referred for surgical assessment and surgery and explore how referral decisions vary with preoperative characteristics. To illustrate how such data could be used to inform policy, we estimated the potential impact of a change in referral criteria: namely basing referrals from the hub to surgical assessment on evidence-based OKS/OHS thresholds, rather than the hub's current referral criteria.

Methods

Outline

We collected data from one UK referral hub and used it to estimate a logistic regression model predicting the probability that patients will be referred from the hub to surgical assessment based on their OKS/OHS, age and sex. We then subsequently applied this regression model to nationally-collected preoperative outcomes data[9-14] to estimate how many patients are referred for surgical assessment nationally. We predicted how many of these patients would be referred through to secondary care if different OKS/OHS thresholds were introduced at the hub and potentially how many extra operations would be performed.

For peer review only - http://bmjopen.bmj.com/site/about/guidelines.xhtml

Hub data collection

Anonymised data were extracted retrospectively from medical records of patients with knee or hip symptoms who had been referred by GPs between July 2015 and July 2016 to the musculoskeletal hub at the Nuffield Orthopaedic Centre (NOC) in Oxford. This study was discussed and agreed through the Clinical Governance Group for hip and knee replacement as part of a wider review of outcomes in hip and knee arthroplasty (Oxford University Hospitals Research and Development Ref 11603). The primary statistical analysis used a prognostic model to estimate how the probability of patients attending face-to-face consultations at the hub being referred to surgical assessments in secondary care varied with OKS/OHS, age and sex. We therefore chose the sample size to provide data on ≥30 knee referrals and ≥30 hip referrals from the hub to secondary care, thereby providing ≥10 events per explanatory variable.[15]

Two medically-qualified surgical research fellows extracted the following: age; sex; OKS/OHS at hub attendance; attendance date; whether the patient was referred to secondary care; date of any subsequent surgical assessment visit; and date of any subsequent arthroplasty surgery. Additional information on imaging, referrals to other clinics, and other surgeries or diagnoses was recorded in free text fields. Data on OKS at the surgical assessment visit were also extracted, when available, from the secondary care records of patients with knee pain. Body mass index (BMI) was not extracted as the analysis focused on OKS/OHS and the available evidence on how capacity to benefit and cost-effectiveness vary with OKS/OHS does not consider BMI.[4 5] Patients who were on the waiting list for arthroplasty surgery at the time of data extraction (August 2016) and those for whom surgery was delayed due to comorbidities or high BMI after their attendance at the surgical assessment consultation were counted as having been referred for surgery.

Exclusion criteria comprised:

- Aged <50 years (for whom knee/hip pain is unlikely to be caused by osteoarthritis).
- Evidence from medical records that symptoms were due to a condition other than osteoarthritis.
- Previous arthroplasty on the same joint.
- Medical records inaccessible for research.
- Any attendance at the hub or surgical assessment unit before July 2015.

However, patients who were referred for x-rays, magnetic resonance imaging (MRI) or physiotherapy but did not attend face-to-face consultations at the hub or surgical assessment unit were included in the descriptive analysis.

For peer review only - http://bmjopen.bmj.com/site/about/guidelines.xhtml

Statistical modelling

We used logistic regression to estimate a prognostic model of the local hub data that predicted how OKS/OHS, age and sex affected the odds of being referred for surgical assessment following a face-to-face attendance at the hub. Age and OKS/OHS at hub attendance were analysed as continuous variables. Explanatory variables were selected based on the Akaike information criterion using forward stepwise regression, manually testing variables in a pre-specified sequence (online supplementary appendix). Analyses were conducted in Stata version 14 (StataCorp, College Station TX) on a complete case basis, excluding patients with missing data. We analysed binary variables using two-sample tests of proportions and analysed continuous variables using unpaired t-tests preceded by F-tests for equal variance. OKS/OHS in the hub sample was compared against population means[9-14] using one-sample t-tests.

Estimating the number of referrals and effect of introducing thresholds

A decision-tree model of the treatment pathway was developed based on hub data and the authors' clinical experience of running hubs and/or surgical clinics. The overall proportion of patients referred directly to surgical assessment and the proportion attending face-to-face hub consultations were calculated from the hub sample. We also estimated the proportion of patients who underwent arthroplasty after being referred from the hub to surgical assessment.

The model then extrapolated hub data to estimate the number of patients in England who are referred for hip or knee replacement (online supplementary appendix). Logistic regression results were used to predict the probability of being referred from the hub to surgical assessment for men and women aged 50, 60, 70, 80 and 90 with different OKS/OHS. For each patient group defined by age, sex and OKS/OHS, we then estimated the number of patients referred to hubs nationally by dividing national data on the number of joint replacements currently conducted for osteoarthritis in England[9-14] by the probability that patients in each group would undergo surgery.

We then conducted an exploratory analysis estimating how the number of referrals to surgical assessment and the number of arthroplasty procedures might change if different OKS/OHS thresholds between 18 and 45 were used to determine referral decisions during face-to-face hub consultations. Although in practice thresholds could be used at various stages in the referral pathway, our analysis focused on the impact of changing referral criteria during face-to-face hub consultations since OKS/OHS data were only routinely available for this patient group.

This analysis made the following assumptions:

For peer review only

For peer review only - http://bmjopen.bmj.com/site/about/guidelines.xhtml

1) Introducing OKS/OHS thresholds at the hub was assumed to have no effect on GP referrals, the hub triage or the probability of a patient with particular preoperative characteristics being referred for arthroplasty following a surgical assessment visit.
2) Since patient records provided no data on OKS/OHS for patients who did not attend the hub, we assumed that the probability of direct referral to surgical assessment and the probability of attending a face-to-face visit at the hub were independent of OKS/OHS, age and sex. Although this assumption may not hold in practice (as symptom severity is one of the main factors considered in the hub triage), it is unlikely to affect estimates of the impact of changing referral criteria solely at the hub.
3) We also assumed that the probability of patients referred to surgical assessment subsequently being referred for surgery was independent of OKS/OHS, age and sex, which was supported by a secondary regression analysis (online supplementary material).
4) We assumed that the referral probabilities estimated from the NOC hub sample are broadly representative of clinical practice across the UK.
5) We assumed, based on the experience of clinical co-authors, that 50% of patients who are currently not referred for surgical assessment after a face-to-face hub visit would still not be considered candidates for arthroplasty (and therefore not referred) regardless of whether thresholds were introduced. This group of patients includes patients who choose not to be referred, those who have not had a complete trial of non-operative treatment and others who are unsuitable for surgery due to comorbidities or other factors. The remaining 50% of patients were assumed to be referred only if their OKS/OHS was below the threshold.
6) Since there are no published data on how OKS/OHS changes over time in the absence of arthroplasty, we assumed for simplicity patients who do not undergo arthroplasty following their hub attendance would not be referred back to clinic for 10 years.
7) Each 40-minute hub attendance was estimated to cost £58 and surgical assessment £132 (online supplementary appendix, Table A1).
8) The analyses took the perspective of the NHS, focusing on costs related to knee/hip arthroplasty. Costs of GP consultations, hub triage, x-rays, imaging, physiotherapy, injections, weight loss programs, missed appointments and referrals to other clinics were excluded as there is no reason to expect the proportion of patients requiring these services to change following the introduction of OKS/OHS thresholds.
9) The reference year for costs was 2014.

The patient numbers calculated within the decision-tree were used to estimate the cost of the referral pathway. The total 10-year costs and total QALYs estimated in a related study[5] for different patient characteristics with and without arthroplasty were applied to the number of patients expected to undergo arthroplasty or have no arthroplasty in each scenario. These figures were used to estimate the net health benefit for each scenario[16 17] (assuming that the NHS is willing/able to pay £20,000 per QALY gained[18]) and the

For peer review only

incremental cost-effectiveness of different pairs of scenarios (online supplementary appendix).

We also estimated the number of additional referrals for surgical assessment that might have been observed within the population of individuals attending the NOC hub if a fixed OKS/OHS threshold were introduced. Based on assumption (5), this was equal to half the number of patients who had OKS/OHS at or below the proposed threshold but were not referred, plus the number who were referred with OKS/OHS below the proposed threshold: for example, if (hypothetically) 60 out of 100 patients were referred and 55 of those referred and 24 of those not referred had OKS<31, we would estimate that 67 (55+24/2) would be referred if a threshold of 31 were introduced.

Public and patient involvement

Patient representatives were involved in the grant application and design of the wider ACHE study.[4] The ACHE research question and study design (prior to funding) was informed by patient interest groups. The current analysis was based on pre-existing data from medical records so no patients were directly recruited in the course of the work. Throughout the ACHE study, a User Group, including patients and other stakeholders, informed progress and development of the work. Results of the analysis were discussed with user group including clinicians and patient representatives, to inform the presentation of results.

Results

Current referral pathway

From the 1,638 records reviewed, we identified 315 patients with knee osteoarthritis and 607 with hip osteoarthritis: 922 in total (Figures 1, 2 and A1, Tables A2-A3). Data on these patients were used to construct the treatment pathway shown in Figure 3.

During the time period for which data were collected, the musculoskeletal hub service was the only route for patients in Oxfordshire to access NHS elective orthopaedic surgery and also directed patients to physiotherapy in the absence of a direct referral physiotherapy service. When GPs refer patients to secondary care with knee/hip symptoms, the referral letter and x-rays are first reviewed by senior hub staff, who decide whether the patient should (a) be referred directly to surgical assessment, (b) attend the hub clinic, or (c) be managed in primary care (Figure 3). Triage is based on the symptoms described in the GP's referral letter (which will include BMI and may include OKS/OHS) and the conservative treatments already given (such as anti-inflammatory drugs, physiotherapy and weight loss strategies).

For peer review only - http://bmjopen.bmj.com/site/about/guidelines.xhtml

Of the osteoarthritis patients included in the analysis, 23% (71/315) of knee patients and 39% (236/607) of hip patients were referred directly to surgical assessment based on the hub triage. In the authors' experience, these patients tend to be those with severe symptoms, those who had already exhausted all conservative measures and those who had previously been referred for surgical assessments, but had chosen not to have surgery at that time. Among 27 patients referred directly to surgical assessment for whom OKS data were available, the mean OKS was 15 (range: 1-30); no such data were available for hip patients.

At the surgical assessment visit, patients discuss the risks and benefits of surgery with an orthopaedic surgeon and make an informed decision about whether or not to undergo arthroplasty or other surgery, taking into account their comorbidities, symptom severity and other factors. Those not undergoing arthroplasty may have interventional radiology, or other operations. Among the patients who were referred directly for surgical assessment and had outcomes data available, 56% (38/68) of knee patients and 69% (161/235) hip patients underwent or were awaiting knee/hip replacements at the time of data extraction.

Following triage, 36% (114/315) of knee patients and 44% (265/607) hip patients did not attend face-to-face consultations at either the hub or surgical assessment. Typically such patients comprise those who can be managed in primary care: for example those with mild symptoms and those who have not yet exhausted conservative treatment options, such as advice and information, activity and exercise and weight loss. Across the 379 hip and knee patients without face-to-face consultations, 270 (71%) had x-rays, MRI or ultrasound to identify the most appropriate care pathway. Eleven (3%) were referred for physiotherapy, while seven patients (2%), all with hip osteoarthritis, had injections. Other patients may not be seen face-to-face as they are unfit for surgery or have recent injuries likely to heal without further intervention. Eighteen patients opted for non-NHS care, missed/failed to book hub appointments, died before the hub attendance or were still awaiting a hub appointment at the time of data extraction (Figures 1 and 2).

The remaining 41% (130/315) of knee patients and 17% (106/607) of hip patients attended face-to-face assessments at the musculoskeletal hub, where extended-scope specialist physiotherapists or orthopaedic fellows assess patients to confirm diagnosis. Patients routinely complete OKS/OHS questionnaires to assess whether the level of symptoms warrants surgery and to guide discussions about the symptom profile. Among the patients attending the hub, the mean OKS was 21 (range: 1-48, Table A3, Figure A1), while mean OHS was 24 (range: 5-46); both values were significantly higher than the average for patients undergoing arthroplasty nationally (mean: 18; $p \leq 0.015$), although only OHS was significantly different from the average for patients undergoing arthroplasty in Oxfordshire (mean OKS: 20, $p=0.134$; mean OHS: 19 $p<0.0001$).[9-14]

For peer review only - http://bmjopen.bmj.com/site/about/guidelines.xhtml

Diagnostic imaging, landmark injections and injections for trochanteric bursitis may be done during the hub consultation. Patients with BMI ≥40 are referred for monitored weight loss programs that must be followed for 12 months before considering surgery. Staff and patients also discuss the risks and benefits of joint replacement (including recovery times, the need for support at home after hospital discharge and the potential need for revision surgery) and how these may be affected by patients' living arrangements and comorbidities. Following the hub visit, 45% (49/130) of knee patients and 36% (36/101) of hip patients were referred for surgical assessment. This includes patients who are considered candidates for arthroplasty or other procedures (e.g. arthroscopy, anterior cruciate ligament repair or interventional radiology).

Patients with higher OKS/OHS were significantly less likely to be referred for surgical assessment: each one-point increase in OKS reduced the odds of referral by 4.7% (p=0.019), while a one-point increase in OHS reduced the odds by 3.9% (p=0.062; Table A4). Those patients who are not referred to surgical assessment may be referred for physiotherapy, other non-surgical management or other outpatient clinics (e.g. rheumatology or sports injury clinics), or may choose not to be referred as they prefer not to undergo surgery at the current time.

Across the hub attendees who were referred for surgical assessment and had data on clinical outcomes, 30% (12/40) of knee patients and 37% (10/27) of hip patients underwent or were awaiting arthroplasty surgery.

Applying estimates of the probability of referral and subsequent arthroplasty from the hub dataset to national data on the distribution of patients undergoing knee/hip arthroplasty suggests that GPs in England refer around 417,000 patients with knee osteoarthritis and around 235,000 patients with hip osteoarthritis to secondary care each year (Figure 3). Of these, around 172,000 knee patients and 41,000 hip patients might attend a hub if the care pathway in Oxfordshire were followed nationally; such hub attendances would cost a total of £12 million.

Effect of introducing referral thresholds

Knees

We used the hub data to estimate the impact that using OKS to guide referral decisions during face-to-face hub consultations might have on patient numbers, costs and health outcomes. Table 1 shows results for thresholds between 18 and 43, although we focus here on the impact of an OKS threshold of 31, since the ACHE study reported that people with pre-operative OKS ≤31 have a ≥70% chance of achieving a seven-point increase in OKS following knee arthroplasty.[4]

For peer review only

Of the 110 knee patients attending the NOC hub, 94 (85%) had OKS ≤31, of whom 47 (50%) were referred for surgical assessment. Two patients were referred with OKS of 32 or 41. Assuming that 50% of the NOC patients with OKS ≤31 who are not currently referred might still choose not to be referred or might be considered clinically inappropriate for other reasons, a fixed referral threshold OKS of 31 would have resulted in an additional 22 referrals to surgical assessment: a 49% increase.

Applying the results of logistic regression to the whole population undergoing surgery nationally suggested that an OKS threshold of 31 might result in 43,000 additional surgical assessment visits and 13,000 additional knee replacement procedures in England each year (Table 1). Introducing this policy could cost the NHS an additional £36 million for each annual cohort of patients (of which £5.8 million would be due to additional surgical assessments), but would gain 30,000 QALYs. This policy would be highly cost-effective compared with current practice, costing just £1,195 per QALY gained. Introducing OKS thresholds between 32 and 43 would produce still greater health benefits and cost less than £20,000/QALY gained.

Hips

Similarly, the ACHE study found that people with preoperative OHS ≤35 have a ≥70% chance of achieving an eight-point increase in OHS following hip arthroplasty.[4] Within the NOC hub, 87% (88/101) of patients had OHS ≤35, of whom 35 were referred. One patient was referred with OHS of 44. Introducing a threshold of 35 might therefore result in 26 additional referrals: a 30% increase.

Extrapolating across England, we might expect 14,000 additional surgical assessments and 5,000 additional hip replacements each year if a threshold of 35 were introduced at the hub (Table 2). The impact of this policy is lower than for knee osteoarthritis as 90% of hip replacements are done in patients who were referred directly, and did not attend the hub. The policy could cost an additional £25 million (of which additional surgical assessments account for £2 million) and gain 16,000 QALYs, costing £1,560/QALY gained. Introducing thresholds between 36 and 45 would produce greater health benefits and cost <£20,000/QALY gained.

Discussion

This retrospective analysis characterised the patients being referred to and from a musculoskeletal hub, and showed that OKS/OHS and other factors were considered when deciding which patients to refer for surgical assessment. Since most patients attending hubs have relatively severe osteoarthritis symptoms and few patients with high OKS/OHS undergo arthroplasty (partly due to the low OKS/OHS thresholds used by some CCGs), introducing evidence-based OKS/OHS thresholds for arthroplasty is unlikely to prevent large

For peer review only - http://bmjopen.bmj.com/site/about/guidelines.xhtml

numbers of inappropriate referrals. However, since recent evidence demonstrates that arthroplasty is both cost-effective and highly likely to benefit people with OKS/OHS well above the thresholds currently used by some CCGs,[4 5] such policies are also likely to identify patients who are not currently referred, but for whom arthroplasty could be expected to be beneficial and cost-effective. Extrapolating from the hub data suggests that setting thresholds of OKS ≤31 and OHS ≤35 would result in many more patients being referred for surgical assessment and surgery, although the exact patient numbers are uncertain and rely on assumptions. More evidence is therefore urgently needed on the other factors currently influencing both the decision to refer from the hub to surgical assessment and the decision on whether arthroplasty is the appropriate treatment option.

To our knowledge, our study is the first to report the characteristics of patients attending a musculoskeletal hub. However, the analysis was based on a small sample from only one hub and included only eight hub attendees with OKS/OHS ≥40. The proportion of patients undergoing surgery after surgical assessment is uncertain as only 22 hub attendees underwent arthroplasty; however, varying the probability of undergoing surgery over its 95% confidence interval did not materially change our conclusions. Data were analysed one month after the end of the one-year period studied; some patients may therefore have gone on to have arthroplasty after physiotherapy or weight loss treatment after our data were extracted. Furthermore, the retrospective review of medical records may not have identified all patients meeting exclusion criteria – particularly for patients who did not attend either the hub or surgical assessment. Furthermore, no data were collected on BMI.

Estimates of the impact of different policies rely on additional assumptions and represent an approximate indication of potential patient numbers; prospective pilot studies would be required to assess the true impact in practice. In particular, we focused on the impact of changing referral guidelines at hubs, as no data were available on patients visiting GPs with osteoarthritis symptoms. In practice, any decision aid or referral guideline is also likely to affect GPs' referral decisions and the hub triage process, which could increase the number of additional operations resulting from any policy change. We assumed that decisions made by the patient and surgeon about whether to proceed to surgery after surgical assessment would be unaffected by the use of thresholds by the hub as OKS/OHS would have already been taken into account at the hub; the budget impact for patients referred via the hub could be lower than shown in Tables 1 and 2 if patients referred with high OKS/OHS were less likely to undergo surgery. We also assumed, arbitrarily, that 50% of people with OKS/OHS below the threshold who are not currently referred would choose not to be referred for surgery even if this were offered. The model also assumed that patients who do not undergo arthroplasty following their hub attendance would not have surgery for 10 years, since there are currently no data on how OKS/OHS change over time without arthroplasty.[5] In practice, many patients who would be eligible for surgery if the OKS/OHS threshold was raised would otherwise have had surgery later, after their condition had

For peer review only

deteriorated. If the OKS/OHS thresholds were raised, the number of operations and budget impact might decrease over time as these patients would have been treated earlier, before their disease progresses, and would not need primary arthroplasty in the future.

Although access to joint replacement may be restricted if funding for interventions is reduced, results suggest that increased numbers of knee/hip replacements could be justified on cost-effectiveness grounds. Our results suggest that the NHS is currently willing to pay no more £2,000 per QALY gained from arthroplasty, while NICE routinely approves treatments for patients with other conditions (e.g. rheumatoid arthritis) that cost £20,000[18] or even £40,000[19]/QALY. This suggests that it may be more efficient and more equitable to spend limited NHS funds on conducting more joint replacements, increasing the operation rate in the UK to a level similar to that of Austria or Germany,[20] rather than directing these resources to other conditions. However, the number of procedures is also limited by availability of surgeons, operating theatres and hospital beds, which may mean that it is not currently feasible to conduct all of the operations that could be justified on cost-effectiveness grounds. And, as noted earlier, OKS/OHS is clearly not the only factor taken into account in decision-making by hubs and surgeons. In particular, patient choice plays a substantial role and comorbidities and other factors are also taken into account.

Referral hubs have previously been shown to be an effective mechanism for referring patients to the most appropriate healthcare interventions/setting and patients are generally satisfied with this model of care.[8] One aim of introducing hubs was to identify patients who do not want surgery or who have mild symptoms and direct them to other effective interventions if surgery is not appropriate. However, the process and criteria for arthroplasty referrals still vary greatly between CCGs.[1] Explicit guidelines and decision aids could help reduce geographical inequity and ensure that all patients likely to benefit have fair access to cost-effective treatments. Although our analysis is based on retrospective data on a small sample from only one hub, it provides initial estimates of what the potential patient numbers and costs might be if a more explicit threshold-based approach was adopted nationally. While arthroplasty rates and patient characteristics in Oxfordshire are similar to the national average,[21 22] referral guidelines and pathways vary substantially between CCGs, suggesting that a larger study covering multiple hubs is necessary to provide a more comprehensive picture of current clinical practice. Ideally this would also collect more detailed patient information (e.g. BMI or comorbidities).

In conclusion, our study demonstrates that musculoskeletal hubs currently take account of OKS/OHS and a variety of other factors when making decisions about referral to surgical assessment and arthroplasty. Introducing evidence-based thresholds for hip/knee replacement based on OKS/OHS, such as the ACHE tool, is likely to prevent very few inappropriate referrals, but identify many more patients who are not currently referred but

For peer review only

for whom arthroplasty is likely to be beneficial and cost-effective. Our results can be used to estimate the impact of other policies, including those where thresholds vary by age or other patient characteristics. However, our estimates of potential patient numbers are approximations relying on numerous assumptions; a multi-centre pilot study would be required to evaluate the actual impact of a policy change on routine clinical practice.

For peer review only

For peer review only - http://bmjopen.bmj.com/site/about/guidelines.xhtml

Declarations

Competing interest statement

All authors have completed the ICMJE uniform disclosure form at www.icmje.org/coi_disclosure.pdf and declare: a grant from NIHR for the submitted work; HD reports personal fees from Halyard Health, outside the submitted work; DB reports grants from Zimmer Biomet, outside the submitted work; AP reports personal fees from Zimmer Biomet and DePuy outside the submitted work; all declare that there are no other relationships or activities that could appear to have influenced the submitted work. The other authors declare that there are no relevant conflicts of interests or funding besides the NIHR grant that supports the current work.

Details of contributors

AP is the guarantor for this study. AP, DB, HD, AG and KB conceived, designed and conducted the ACHE study. HD, AP and JS conceived and designed the study, which was conducted by HD and JS. HD, AG and PE conceived and designed the economic evaluation. HD conducted the statistical and economic analysis under the supervision of AG. AP, DB and KB helped interpret the results. HD drafted the manuscript. All authors edited the manuscript for important intellectual content and approved the final version.

Copyright/licence

The Corresponding Author has the right to grant on behalf of all authors and does grant on behalf of all authors, an exclusive licence (or non-exclusive for government employees) on a worldwide basis to the BMJ Publishing Group Ltd and its Licensees to permit this article (if accepted) to be published in BMJ Open and any other BMJPGL products to exploit all subsidiary rights, as set out in our licence http://authors.bmj.com/submitting-your-paper/copyright-and-authors-rights/ and the Corresponding Author accepts and understands that any supply made under these terms is made by BMJPGL to the Corresponding Author. Where the Corresponding Author wishes to make the article available on an open access basis (and intends to pay the relevant open access fee), the terms of such open access shall be governed by a Creative Commons licence – details of these licences and which Creative Commons licence will apply to this article are set out in our licence referred to above.

Ethics statement

Trust management approval from the Oxford University Hospitals NHS Trust was obtained for retrospective analysis of anonymised data from medical records concerning outcomes in hip and knee arthroplasty (Oxford University Hospitals Research and Development Reference 11603). The plan to study referral patterns through the musculoskeletal hub, under the terms of the research and development agreement noted above, was discussed and agreed through the Clinical Governance Group for hip and knee replacement. The

For peer review only

analysis was conducted to improve service understanding and used retrospective data on patients who had received normal clinical management. All data were anonymised by clinical staff.

Funding and role of study sponsors

The ACHE study and economic evaluation was funded by the NIHR Health Technology Assessment Programme (project number 11/63/01) and will be published in full in *Health Technology Assessment*. Further information available at: https://www.journalslibrary.nihr.ac.uk/programmes/hta/116301. This report presents independent research commissioned by the National Institute for Health Research (NIHR). AG was supported by the NIHR Biomedical Research Centre, Oxford. The views and opinions expressed by authors in this publication are those of the authors and do not necessarily reflect those of the NHS, the NIHR, MRC, CCF, NETSCC, the Health Technology Assessment Programme, NHS, NHS Digital or the Department of Health. The funder had no involvement in: the collection, analysis, and interpretation of data; in the writing of the report; and in the decision to submit the article for publication. The authors had full access to the data (including statistical reports and tables), can take responsibility for the integrity of the data and the accuracy of the data analysis and are responsible for submitting this paper.

Transparency declaration

The manuscript's guarantor (AP) affirms that the manuscript is an honest, accurate, and transparent account of the study being reported; that no important aspects of the study have been omitted; and that any discrepancies from the study as planned (and, if relevant, registered) have been explained.

Data sharing

The decision tree and Markov models used in this paper are available from the corresponding author on request. PROMs/HES data are available from NHS Digital (http://content.digital.nhs.uk/dars).

Acknowledgements

We would like to thank the ACHE patient and public representatives, user group and steering group for their role in the project: Anne Clarkson Webb, Anthony Johnson, Chad Lion Cachet, Fiona Watt, Fraser Old, Gill Dean, Gillian Kempster, Jannie Kramer, Jennifer Bostock, Jiyang Li, Jo Hewanicka, John Nolan, Kate Jackson, Laura Ingle, Mary Snow, Matthew Cheetham, Patricia Mary Rubery, Sharon Barrington, Sujin Kang, Tim Wilton, Vida Field, Sunny Deo, Malvin Drakely, Jon Campion, George Peat, Peter Kay, Jon Waite and Sue Woollacott. We would like to extend thanks to Reza Mafi, and Sarah Dorman who helped with data cleaning. We would also like to thank the reviewers for their insightful comments on the manuscript and Emiko Sykes for proofreading the manuscript. Data on NHS PROMs

For peer review only - http://bmjopen.bmj.com/site/about/guidelines.xhtml

linked to HES APC data were reused with the permission of NHS Digital, copyright 2015, with all rights reserved.

References

1. The Royal College of Surgeons of England. Is access to surgery a postcode lottery? 2014. https://www.rcseng.ac.uk/news-and-events/media-centre/press-releases/many-ccgs-are-ignoring-clinical-evidence-in-their-surgical-commissioning-policies/ (accessed 8 November 2016).
2. Harrogate and Rural District Clinical Commissioning Group. Clinical thresholds: Hip and knee arthroplasty for osteoarthritis (only). 2014. http://www.harrogateandruraldistrictccg.nhs.uk/data/uploads/rss2/hip-and-knee-arthroplasty.pdf (accessed 8 November 2016).
3. Scarborough and Ryedale Clinical Commissioning Group. Hip replacement pathway. 2015. http://www.scarboroughryedaleccg.nhs.uk/data/uploads/rss2/orthopaedics/hip-replacement-march-2015.pdf (accessed 8 November 2016).
4. Anonymised for peer review. The use of patient-reported outcome measures to guide referral for hip and knee replacement: Part 1 – the development of an evidence based model linking pre-operative score to the probability of gaining benefit from surgery. Submitted to Bone and Joint Journal 2019
5. Anonymised for peer review, on behalf of the ACHE Study team. The use of patient-reported outcome measures to guide referral for hip and knee replacement: Part 2 – a cost-effectiveness analysis. Submitted to Bone and Joint Journal 2019
6. Podmore B, Hutchings A, Durand MA, et al. Comorbidities and the referral pathway to access joint replacement surgery: an exploratory qualitative study. BMC health services research 2018;**18**(1):754 doi: 10.1186/s12913-018-3565-0.
7. Button K, Morgan F, Hodgson H, Weightman AL, Jones S. Musculoskeletal care pathways for adults with hip and knee pain at the interface between primary and secondary care: protocol for a systematic review. Syst Rev 2016;**5**(1):128 doi: 10.1186/s13643-016-0301-x.
8. Candy E, Haworth-Booth S, Knight-Davis M. Review of the Effectiveness of a Consultant Physiotherapy-Led Musculoskeletal Interface Team: A Welsh Experience. Musculoskeletal Care 2016;**14**(3):185-91 doi: 10.1002/msc.1122.
9. Health and Social Care Information Centre. Finalised Patient Reported Outcome Measures (PROMs) in England, April 2014-March 2015 (annual report). 2016. http://content.digital.nhs.uk/catalogue/PUB21189/final-proms-eng-apr14-mar15-fin-report.pdf (accessed 2 November 2016).
10. Health and Social Care Information Centre. Finalised Patient Reported Outcome Measures (PROMs) in England, April 2013 to March 2014. 2015. http://www.hscic.gov.uk/catalogue/PUB17876/final-proms-eng-apr13-mar14-fin-report-v1.pdf (accessed 6 April 2016).

For peer review only - http://bmjopen.bmj.com/site/about/guidelines.xhtml

11. Health and Social Care Information Centre. Finalised Patient Reported Outcome Measures (PROMs) in England, April 2012 to March 2013 (annual report). 2014. http://content.digital.nhs.uk/catalogue/PUB14574/final-proms-eng-apr12-mar13-fin-report-v1.pdf (accessed 2 November 2016).
12. Health and Social Care Information Centre. Finalised Patient Reported Outcome Measures (PROMs) in England, April 2011 to March 2012 (annual report). 2013. http://content.digital.nhs.uk/catalogue/PUB11359/final-proms-eng-apr11-mar12-fin-report-v2.pdf (accessed 2 November 2016).
13. Health and Social Care Information Centre. Finalised Patient Reported Outcome Measures (PROMs) in England, April 2010 to March 2011 (annual report). 2012. http://content.digital.nhs.uk/catalogue/PUB07049/fina-prom-eng-apr-10-mar-11-pre-post-rep1.pdf (accessed 10 November 2016).
14. Health and Social Care Information Centre. Finalised Patient Reported Outcome Measures (PROMs) in England, April 2009 to March 2010 (annual report). 2011. http://content.digital.nhs.uk/catalogue/PUB02429/proms-eng-apr-09-mar-10-oper-data-expe-meth.pdf (accessed 10 November 2016).
15. Moons KG, Royston P, Vergouwe Y, Grobbee DE, Altman DG. Prognosis and prognostic research: what, why, and how? BMJ 2009;**338**:b375 doi: 10.1136/bmj.b375.
16. Coyle D, Buxton MJ, O'Brien BJ. Stratified cost-effectiveness analysis: a framework for establishing efficient limited use criteria. Health Econ 2003;**12**(5):421-7 doi: 10.1002/hec.788.
17. Espinoza MA, Manca A, Claxton K, Sculpher MJ. The value of heterogeneity for cost-effectiveness subgroup analysis: conceptual framework and application. Med Decis Making 2014;**34**(8):951-64 doi: 10.1177/0272989X14538705.
18. National Institute for Health and Clinical Excellence. Social value judgements: Principles for the development of NICE guidance. Second Edition. 2008. https://www.nice.org.uk/Media/Default/About/what-we-do/Research-and-development/Social-Value-Judgements-principles-for-the-development-of-NICE-guidance.pdf (accessed 17th November 2010).
19. Dakin H, Devlin N, Feng Y, Rice N, O'Neill P, Parkin D. The Influence of Cost-Effectiveness and Other Factors on Nice Decisions. Health Econ 2015;**24**(10):1256-71 doi: 10.1002/hec.3086.
20. OECD. Hip and knee replacement. Health at a Glance 2015: OECD, 2015:112–13.
21. Right Care. The NHS Atlas of variation in health care: reducing unwarranted variation to increase value and improve quality. 2010. http://fingertips.phe.org.uk/documents/Atlas_2011_MSK.pdf (accessed 18 July 2017).
22. Health and Social Care Information Centre. Finalised Patient Reported Outcome Measures in England - April 2014 to March 2015: Score Comparison Tool. 2016. http://content.digital.nhs.uk/catalogue/PUB21189 (accessed 11 November 2016).

For peer review only

23. Hospital Episode Statistics. Admitted patient care England 2014-15: Procedures and interventions. 2015. http://www.hscic.gov.uk/catalogue/PUB19124/hosp-epis-stat-admi-proc-2014-15-tab.xlsx (accessed 6 April 2016).

For peer review only

Table 1 Estimates of the potential impact of different OKS thresholds on patient numbers, costs and QALYs among the 172,192 patients with knee osteoarthritis attending the hub in England each year. The results presented exclude patients who did not attend face-to-face consultations at the hub; based on our analysis, 31% (24,063/76,617) of knee replacements are conducted on patients who attended the hub.

	Current practice	Maximum OKS at which patients can be referred for surgical assessment					
		18	24	31‡	35	41§	43¥
Number of attendances at the surgical outpatient visit	80,211	65,286	99,982	123,635	129,256	131,810	131,983
Number of arthroplasty procedures conducted (% change*)	24,063	19,586 (-6%)	29,995 (+8%)	37,090 (+17%)	38,777 (+19%)	39,543 (+20%)	39,595 (+20%)
Total cost over 10 years (thousands)	£1,098,213	£1,081,313	£1,108,836	£1,134,134	£1,142,073	£1,146,346	£1,146,678
Total QALYs over 10 years	564,744	566,658	585,674	594,802	596,910	597,825	597,856
Net health benefit (QALYs)†	509,834	512,593	530,232	538,096	539,806	540,508	540,522

* Percentage change in the total number of arthroplasty procedures following a change to referral patterns at the hub. Equal to the difference in the number of procedures between the scenario in question and 'current practice', divided by the 76,617 knee replacements conducted in England each year.[23]

† Net health benefit = QALYs – Cost/£20,000 and indicates the QALYs for each scenario, minus the health benefits that would be foregone by spending money on knee arthroplasty candidates, rather than other conditions.

‡ Threshold at which 70% of patients are predicted to achieve a 7-point improvement in OKS [4].

§ ACHE absolute threshold, above which patients cannot achieve a 7-point improvement in OKS [4].

¥ ACHE economic threshold, above which arthroplasty is not cost-effective (i.e. costs >£20,000/QALY gained) [5].

For peer review only

Table 2 Estimates of the potential impact of different OHS thresholds on patient numbers, costs and QALYs among the 41,121 patients with hip osteoarthritis attending the hub in England each year. The results presented exclude patients who did not attend face-to-face consultations at the hub; based on our analysis, 9% (6,590/69,313) of hip replacements are conducted on patients who attended the hub.

	Current practice	Maximum OHS at which patients can be referred for surgical assessment					
		18	24	30	35‡	40§	45¥
Number of attendances at the surgical outpatient visit	17,793	18,036	25,420	29,871	31,561	32,108	32,216
Number of arthroplasty procedures conducted (% change*)	6,590	6,680 (+0%)	9,415 (+4%)	11,063 (+6%)	11,689 (+7%)	11,892 (+8%)	11,932 (+8%)
Total cost over 10 years (thousands)	£168,402	£168,202	£182,080	£190,519	£193,753	£194,816	£195,030
Total QALYs over 10 years	123,674	128,990	135,863	138,888	139,929	140,248	140,288
Net health benefit (QALYs)†	115,254	120,580	126,759	129,362	130,242	130,508	130,537

* Percentage change in the total number of arthroplasty procedures following a change to referral patterns at the hub. Equal to the difference in the number of procedures between the scenario in question and 'current practice', divided by the 69,313 hip replacements conducted in England each year.[23]

† Net health benefit = QALYs – Cost/£20,000 and indicates the QALYs for each scenario, minus the health benefits that would be foregone by spending money on hip arthroplasty candidates, rather than other conditions.

‡ Threshold at which 70% of patients are predicted to achieve an 8-point improvement in OHS [4].

§ ACHE absolute threshold, above which patients cannot achieve an 8-point improvement in OHS [4].

¥ ACHE economic threshold, above which arthroplasty is not cost-effective (i.e. costs >£20,000/QALY gained) [5].

For peer review only

Figure 1 Patient flow diagram for patients referred with knee symptoms

* See Table A2 in online supplementary appendix for a list of the conditions other than osteoarthritis for which patients aged ≥50 years were excluded from the analysis.

For peer review only

Figure 2 Patient flow diagram for patients referred with hip symptoms

* See Table A2 in online supplementary appendix for a list of the conditions other than osteoarthritis for which patients aged ≥50 years were excluded from the analysis.

For peer review only

Figure 3 Number of patients predicted to be referred with (A) knee and (B) hip osteoarthritis symptoms in England and mean Oxford knee/hip score (OKS/OHS) for the groups for which data are available.

For peer review only

For peer review only - http://bmjopen.bmj.com/site/about/guidelines.xhtml

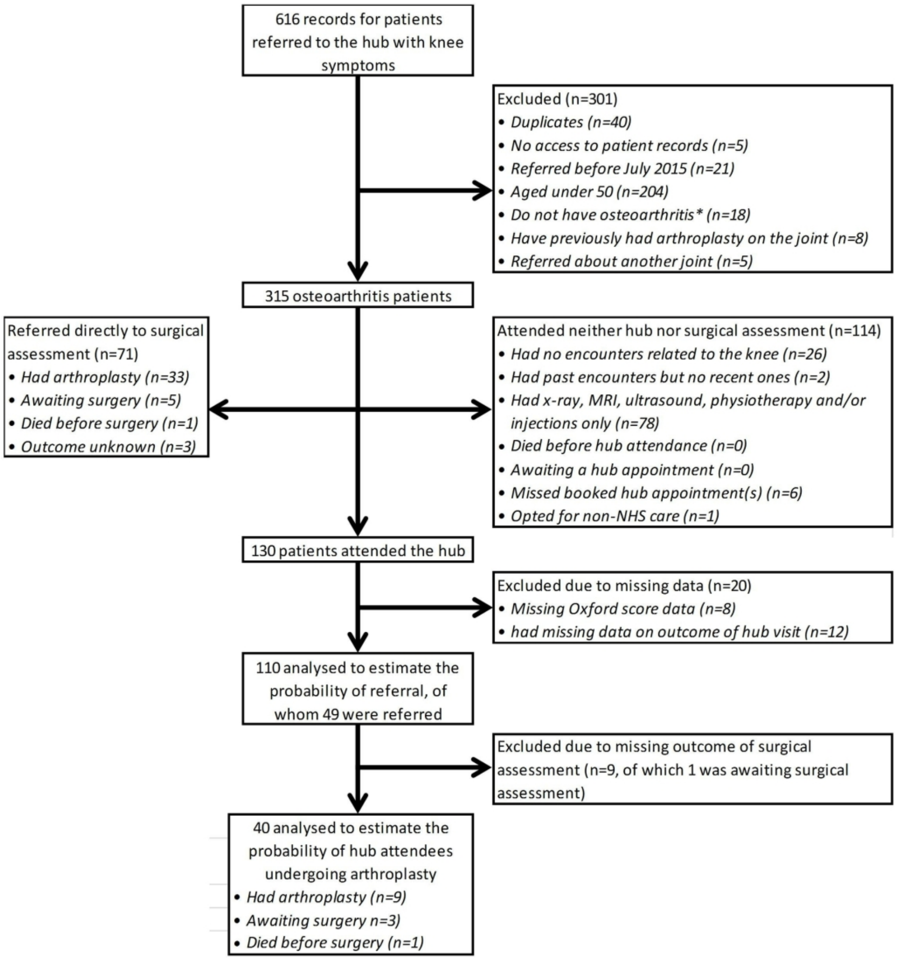

Figure 1 Patient flow diagram for patients referred with knee symptoms

* See Table A2 in online supplementary appendix for a list of the conditions other than osteoarthritis for which patients aged ≥50 years were excluded from the analysis.

190x253mm (300 x 300 DPI)

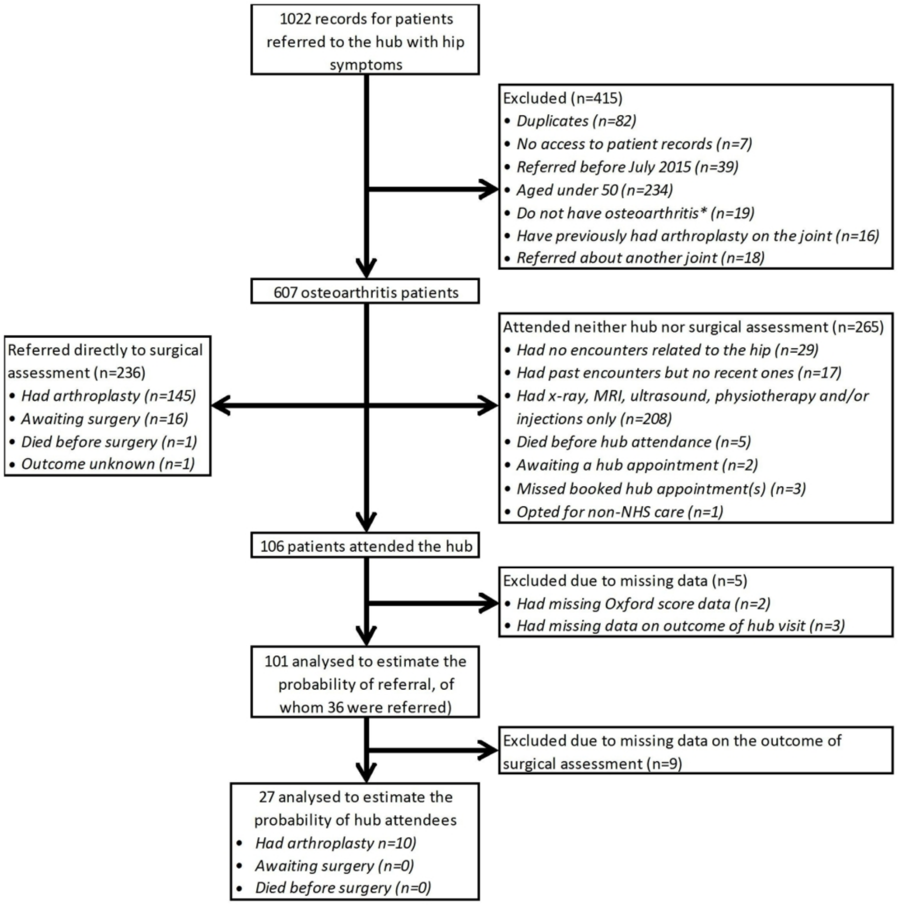

Figure 2 Patient flow diagram for patients referred with hip symptoms

* See Table A2 in online supplementary appendix for a list of the conditions other than osteoarthritis for which patients aged ≥50 years were excluded from the analysis.

190x253mm (300 x 300 DPI)

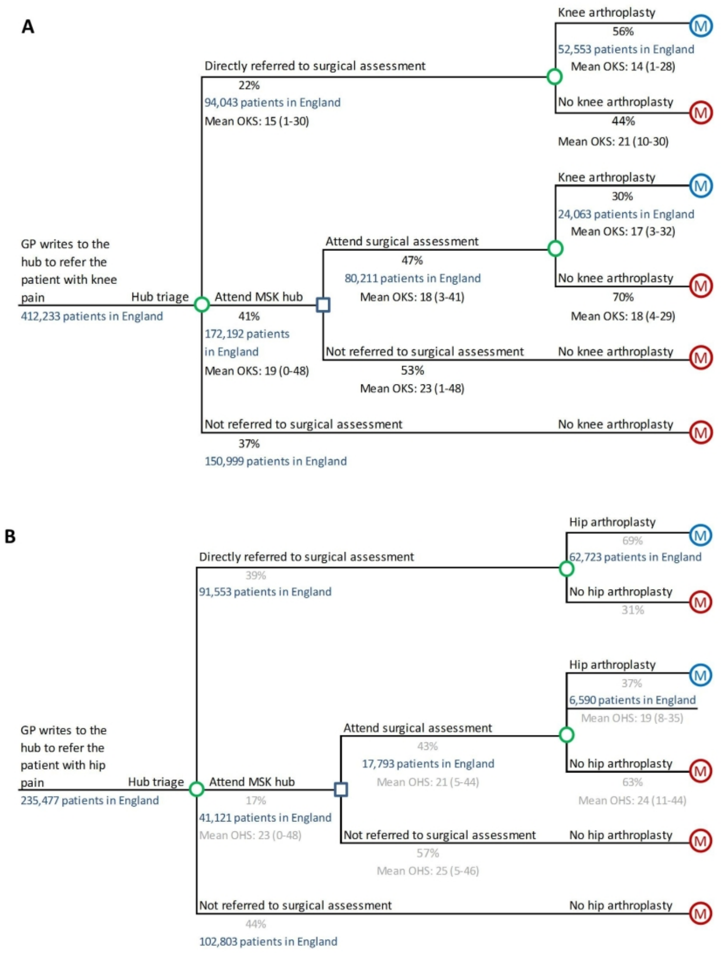

Figure 3 Number of patients predicted to be referred with (A) knee and (B) hip osteoarthritis symptoms in England and mean Oxford knee/hip score (OKS/OHS) for the groups for which data are available.

190x274mm (284 x 284 DPI)

Online supplementary file

Methods for stepwise regression models

We selected variables for inclusion in the logistic regression model in a pre-specified sequence. We first estimated a model only including the constant term and evaluated whether adding OKS/OHS to the model reduced Akaike information criterion (AIC); if AIC was reduced, the model used in the next step controlled for OKS/OHS and otherwise remained with just a constant term. Similarly, the next step assessed whether adding age into the model reduced AIC and in the final step we assessed whether adding sex into the model reduced AIC. We specified the order in which variables were considered for the model (OKS/OHS, then age, then sex) in advance of data analysis: it was hypothesised that OKS/OHS would be the most important factor determining referrals, followed by age (since this is a proxy for comorbidities) and finally sex (since we had no prior hypotheses about how sex would affect referrals). Age and OKS/OHS were assumed to have linear effects on the log-odds of being referred for surgical assessment as there were insufficient data to evaluate nonlinear functions.

Methods for estimating patient numbers

Hub data were extrapolated to estimate patient numbers across England by applying the probability of referral estimated on the hub data to national data on the characteristics of patients undergoing arthroplasty and the total number of operations.

We considered 490 groups of patients, defined by gender, age (using age bands <55, 55-64, 65-74, 75-84 and ≥85) and individual preoperative OKS/OHS between 0 and 48. An extract of individual patient data from the national patient reported outcome measures (PROMs) dataset linked to Hospital Episode Statistics (HES) covering operations conducted between 2009 and 2015 was used to estimate the proportion of arthroplasty patients in different groups defined by age and gender. The same dataset was used to obtain the proportion of patients with each preoperative OKS/OHS.

The number of operations in each of the 490 patients groups was calculated by multiplying the proportion of patients in different age and sex groups by the proportion with different OKS/OHS and by the number of primary knee/hip replacements conducted solely for osteoarthritis in England in 2014-15. The total number of primary knee/hip replacements conducted for osteoarthritis (76,617 knee replacements and 69,313 hip replacements) was calculated by multiplying the total number of finished consultant episodes in HES[1] with the OPCS codes beginning with O or W that the National joint Registry (NJR) used to identify primary hip/knee replacement[2] (79,726 knee replacements and 77,880 hip replacements) by the proportion of primary arthroplasty procedures that are conducted solely for osteoarthritis (96% for knees and 89% for hips).[3]

For peer review only - http://bmjopen.bmj.com/site/about/guidelines.xhtml

We then calculated the number of patients in each group who are likely to have been referred by GPs with knee/hip symptoms by dividing the number of operations in each group by the probability that each group of patients will undergo surgery.

Unit costs

The unit costs used in the current analysis are shown in Table A1. The costs and QALYs accrued in the 10-year period after arthroplasty (or a decision not to perform arthroplasty) have been reported previously.[4]

Table A1 Unit costs

Healthcare resource	Cost per consultation	Reference and details
Attendance at musculoskeletal hub	£58	Based on 40 minutes' of patient-related activity for a grade 8a physiotherapist. Based on activity at the NOC hub, each hub attendance lasts 30 minutes, plus an additional 10 minutes' administration per patient and will, on average, be conducted by a grade 8a physiotherapist who spends around 80% of their time on patient-related activity. The salary for a band 8a, point 36 physiotherapist was based on Agenda for Change salaries[5] and converted to an hourly rate by applying the same methodology as was applied by PSSRU for a hospital physiotherapist,[6] assuming that working hours and the percentages for salary on costs, qualifications and overheads would be the same for all hospital physiotherapists.
Outpatient consultation with an orthopaedic surgeon	£132	National average unit cost for first consultant-led non-admitted face-to-face attendance, WF01B, CL sheet of the National Schedule of Reference Costs Year 2013-14 NHS trusts and NHS foundation trusts[7]

For peer review only

For peer review only - http://bmjopen.bmj.com/site/about/guidelines.xhtml

Additional results

Table A2 Conditions other than osteoarthritis for which patients aged ≥50 years were excluded from the analysis

	Number of patients	
	**Knee audit**	**Hip audit**
Total patients aged ≥50 excluded due to conditions other than osteoarthritis	18	19
Rheumatoid arthritis	5	9
Bone or joint infections	4	2
Requiring limb reconstruction	1	2
Injury	2	1
No mechanical symptoms	1	1
Fracture	0	3
Arthritis secondary to fracture	2	0
Symptoms caused by previous spinal surgery	0	1
Gout	1	0
Chondroid lesion	1	0
Quadriceps rupture	1	0

Table A3 Characteristics of the patients included in the hub audit

	**Number of patients**	**Mean age (range)**	**% Female (n/N)**	**Mean OKS/OHS (range; N)**
Knee patients				
All osteoarthritis patients	315	66 (50, 93)	52% (165/315)	-
All patients referred directly to surgical assessment	71	68 (51, 86)	61% (43/71)	15 (1, 30; N=27)
Patients not referred for arthroplasty following direct referral to surgical assessment	*30*	*63 (51, 86)*	*53% (16/30)*	*21 (10, 30; N=5)*
Patients referred for arthroplasty following direct referral to surgical assessment	*38*	*72 (55, 86)*	*63% (24/38)*	*14 (1, 28; N=22)*
Patients attending neither hub nor surgical assessment	114	66 (50, 86)	49% (56/114)	-
All patients attending the hub	130	65 (50, 93)	51% (66/130)	19 (0, 48; N=130)
Patients not referred to surgical assessment following hub attendance	*61*	*64 (50, 88)*	*44% (27/61)*	*23 (1, 48; N=61)*
Patients referred to surgical assessment following hub attendance	*49*	*65 (51, 88)*	*49% (24/49)*	*18 (3, 41; N=49)*

For peer review only - http://bmjopen.bmj.com/site/about/guidelines.xhtml

	Number of patients	Mean age (range)	% Female (n/N)	Mean OKS/OHS (range; N)
Patients referred for arthroplasty following hub attendance and surgical assessment	*12*	*66 (55, 88)*	*33% (4/12)*	*17 (3, 32; N=12)*
Patients not referred for arthroplasty following hub attendance and surgical assessment	*28*	*65 (51, 82)*	*57% (16/28)*	*18 (4, 29; N=28)*
Hip patients				
All osteoarthritis patients	607	70 (50, 95)	67% (407/607)	-
All patients referred directly to surgical assessment	236	72 (50, 95)	67% (158/236)	-
Patients not referred for arthroplasty following direct referral to surgical assessment	*74*	*71 (51, 95)*	*80% (59/74)*	-
Patients referred for arthroplasty following direct referral to surgical assessment	*161*	*72 (50, 92)*	*61% (98/161)*	-
Patients attending neither hub nor surgical assessment	265	70 (50, 93)	64% (170/265)	-
All patients attending the hub	106	67 (50, 89)	75% (79/106)	23 (0, 48; N=106)
Patients not referred to surgical assessment following hub attendance	*65*	*67 (51, 89)*	*80% (52/65)*	*25 (5, 46; N=65)*
Patients referred to surgical assessment following hub attendance	*36*	*67 (50, 85)*	*67% (24/36)*	*21 (5, 44; N=36)*
Patients referred for arthroplasty following hub attendance and surgical assessment	*10*	*72 (56, 85)*	*50% (5/10)*	*19 (8, 35; N=10)*
Patients not referred for arthroplasty following hub attendance and surgical assessment	*17*	*67 (51, 84)*	*65% (11/17)*	*24 (11, 44; N=17)*

For peer review only - http://bmjopen.bmj.com/site/about/guidelines.xhtml

FIGURE A1 Distribution of (**A**) OKS and (**B**) OHS for patients attending the hub with knee/hip pain compared with the distribution of patients undergoing knee arthroplasty in England in PROMs/HES (2009-2015)

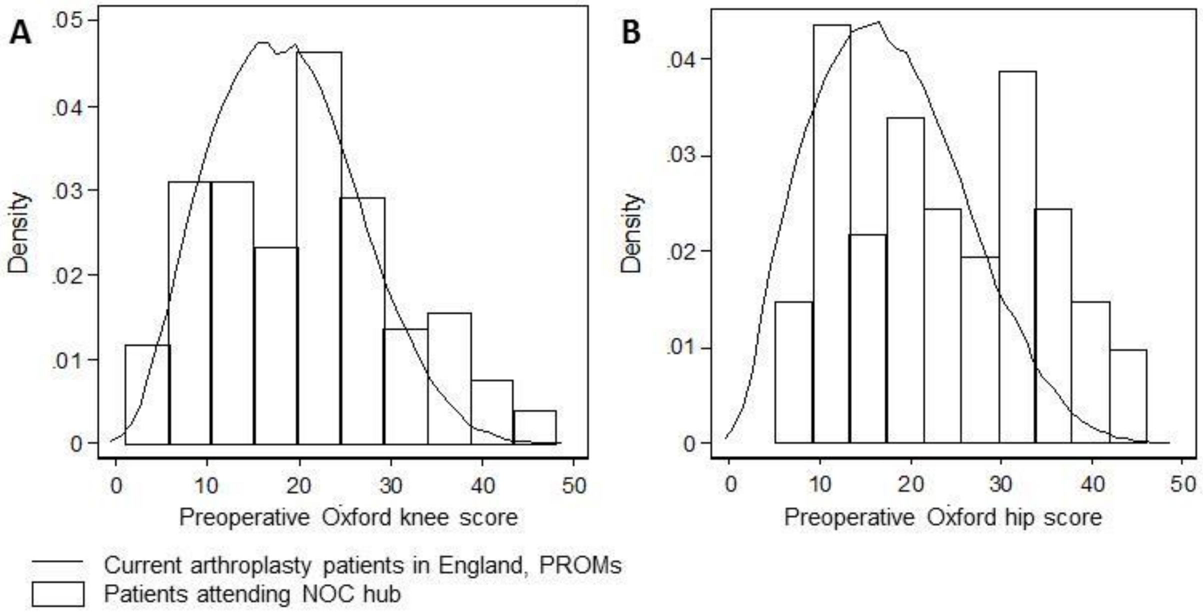

Additional regression results

For patients attending the hub with knee symptoms, adding age and sex into the model predicting the odds of hub attendees being referred for surgical assessment increased AIC, so the final model included only OKS (Table A4). Similarly, in the model of hip patients, adding age into the model worsened AIC, so the final model included only OHS and gender. Omitting gender from the model had negligible effect on the coefficient for OHS, which was 0.0397 (SE: 0.0211) when OHS was the only explanatory variable.

TABLE A4 Results of logistic regression predicting the odds of patients attending the hub being referred for surgical assessment

Variable	Mean coefficient (SE)	
	**Knee patients (N=110)**	**Hip patients (N=101)**
OKS/OHS	-0.048 (0.021)*	-0.0397 (0.0213)
Male	-	0.6965 (0.4804)
Constant	0.768 (0.459)	0.1428 (0.5376)
Pseudo-R^2	0.0389	0.0441

* p<0.05

In addition to the logistic regression model predicting the odds of patients being referred from the hub to surgical assessment, we also conducted a secondary analysis predicting the odds of undergoing arthroplasty in patients who had already been referred to surgical assessment by the hub, using the same methods. This analysis suggested that OKS/OHS, age and sex had no significant effect on the odds of referral from surgical assessment to surgery

For peer review only - http://bmjopen.bmj.com/site/about/guidelines.xhtml

(p>0.05) and that including any of these variables in the regression increased AIC compared with a model with only a constant term. This validates the assumption that the probability of patients referred directly to surgical assessment subsequently being referred for surgery is independent of OKS/OHS, age and sex and the assumption that introducing OKS/OHS thresholds at the hub would have no effect on the probability of a patient with particular preoperative characteristics being referred for arthroplasty following a surgical assessment visit. However, this analysis is likely to be underpowered, since only 12 knee patients and 10 hip patients underwent surgery after face-to-face consultations at the hub and surgical assessment.

References

1. Hospital Episode Statistics. Admitted patient care England 2014-15: Procedures and interventions. 2015. http://www.hscic.gov.uk/catalogue/PUB19124/hosp-epis-stat-admi-proc-2014-15-tab.xlsx (accessed 6 April 2016).
2. National Joint Registry for England and Wales. OPCS codes relevant procedures recorded in the NJR. 2013. http://www.njrcentre.org.uk (accessed 29 September 2016).
3. National Joint Registry for England and Wales. 13th Annual Report. 2016. www.njrcentre.org.uk (accessed 10 October 2016).
4. Dakin H, Eibich P, Beard D, Gray A, Price A, on behalf of the ACHE Study team. The use of patient-reported outcome measures to guide referral for hip and knee replacement: Part 2 – a cost-effectiveness analysis. Submitted to Bone and Joint Journal 2019
5. NHS Employers. Agenda for change pay bands and points from 1 April 2014. 2014. http://www.nhsemployers.org/~/media/Employers/Documents/Pay%20and%20reward/AfC%20pay%20bands%20and%20points%20poster%202014.pdf (accessed 19 October 2016).
6. Curtis L. Unit Costs of Health and Social Care 2014. 2014. http://www.pssru.ac.uk/project-pages/unit-costs/2014/ (accessed 22 June 2015).
7. Department of Health. NHS reference costs 2013 to 2014 - National schedule of reference costs: the main schedule. 2014. https://www.gov.uk/government/publications/nhs-reference-costs-2013-to-2014 (accessed 22 June 2015).

For peer review only

STROBE 2007 (v4) Statement—Checklist of items that should be included in reports of *cross-sectional studies*

Section/Topic	Item #	Recommendation	Section and page number where reported
**Title and abstract**	1	(*a*) Indicate the study's design with a commonly used term in the title or the abstract	Title pg 1
		(*b*) Provide in the abstract an informative and balanced summary of what was done and what was found	Title pg 1
**Introduction**			
Background/rationale	2	Explain the scientific background and rationale for the investigation being reported	Introduction pg 5
Objectives	3	State specific objectives, including any prespecified hypotheses	Introduction pg 5
**Methods**			
Study design	4	Present key elements of study design early in the paper	Strengths and limitations pg 5
Setting	5	Describe the setting, locations, and relevant dates, including periods of recruitment, exposure, follow-up, and data collection	Methods page 6
Participants	6	(*a*) Give the eligibility criteria, and the sources and methods of selection of participants	Methods page 6
Variables	7	Clearly define all outcomes, exposures, predictors, potential confounders, and effect modifiers. Give diagnostic criteria, if applicable	Methods page 6
Data sources/ measurement	8*	For each variable of interest, give sources of data and details of methods of assessment (measurement). Describe comparability of assessment methods if there is more than one group	Methods page 6
Bias	9	Describe any efforts to address potential sources of bias	*N/A*
Study size	10	Explain how the study size was arrived at	Methods page 6
Quantitative variables	11	Explain how quantitative variables were handled in the analyses. If applicable, describe which groupings were chosen and why	Methods page 6-7
Statistical methods	12	(*a*) Describe all statistical methods, including those used to control for confounding	Methods pages 6-7 and page 1 of online supplementary appendix
		(*b*) Describe any methods used to examine subgroups and interactions	N/A
		(*c*) Explain how missing data were addressed	Methods page7

For peer review only

		(*d*) If applicable, describe analytical methods taking account of sampling strategy	N/A
		(*e*) Describe any sensitivity analyses	N/A
**Results**			
Participants	13*	(a) Report numbers of individuals at each stage of study—eg numbers potentially eligible, examined for eligibility, confirmed eligible, included in the study, completing follow-up, and analysed	Figures 1-2 pages 22-23 and 25-26
		(b) Give reasons for non-participation at each stage	Figures 1-2 (pages 22-23 and 25-26) and Table A2 (page 3 of online supplementary appendix)
		(c) Consider use of a flow diagram	Figures 1-2 (pages 22-23 and 25-26)
Descriptive data	14*	(a) Give characteristics of study participants (eg demographic, clinical, social) and information on exposures and potential confounders	Table A3, Figure A1 (pages 3-5 of online supplementary appendix)
		(b) Indicate number of participants with missing data for each variable of interest	Figures 1-2 (pages 22-23 and 25-26) and Table A3 (pages 3-4 of online supplementary appendix)
Outcome data	15*	Report numbers of outcome events or summary measures	Figures 1-3 (pages 22-27)
Main results	16	(*a*) Give unadjusted estimates and, if applicable, confounder-adjusted estimates and their precision (eg, 95% confidence interval). Make clear which confounders were adjusted for and why they were included	Online supplementary appendix pages 5-6
		(*b*) Report category boundaries when continuous variables were categorized	N/A
		(*c*) If relevant, consider translating estimates of relative risk into absolute risk for a meaningful time period	N/A
Other analyses	17	Report other analyses done—eg analyses of subgroups and interactions, and sensitivity analyses	Methods pages 6-8 and online supplementary file pages 5-6
**Discussion**			
Key results	18	Summarise key results with reference to study objectives	Discussion, paragraph 1 (page 12)
Limitations	19	Discuss limitations of the study, taking into account sources of potential bias or imprecision. Discuss both direction and magnitude of any potential bias	Discussion, paragraphs 2-3 (pages 12-13

Interpretation	20	Give a cautious overall interpretation of results considering objectives, limitations, multiplicity of analyses, results from similar studies, and other relevant evidence	Discussion (pages 12-14
Generalisability	21	Discuss the generalisability (external validity) of the study results	Discussion, paragraph 5 (page 14)
**Other information**			
Funding	22	Give the source of funding and the role of the funders for the present study and, if applicable, for the original study on which the present article is based	Funding and role of study sponsors (page 16)

*Give information separately for cases and controls in case-control studies and, if applicable, for exposed and unexposed groups in cohort and cross-sectional studies.

Note: An Explanation and Elaboration article discusses each checklist item and gives methodological background and published examples of transparent reporting. The STROBE checklist is best used in conjunction with this article (freely available on the Web sites of PLoS Medicine at http://www.plosmedicine.org/, Annals of Internal Medicine at http://www.annals.org/, and Epidemiology at http://www.epidem.com/). Information on the STROBE Initiative is available at www.strobe-statement.org.

For peer review only
